# Panunoids A – D, four new prenylhydroquinone derivatives isolated from the fungus *Panus rudis*

**DOI:** 10.1007/s13659-025-00562-3

**Published:** 2026-01-09

**Authors:** Yun Liu, Jian-Qiang Zhao, Yan-Long Yang, Han-Bing Yuan, Yan-Ming Wang, Jun Yuan

**Affiliations:** 1 Jiangsu Key Laboratory of Regional Specific Resource Pharmaceutical Transformation, Huaiyin Institute of Technology, Huai’an, 223003 China; 2 State Key Laboratory of Applied Organic Chemistry, College of Chemistry and Chemical Engineering, Lanzhou University, Lanzhou, 730000 China; 3 National & Local Joint Engineering Research Center for Mineral Salt Deep Utilization, Huaiyin Institute of Technology, Huai’an, 223003 China; 4 Key Laboratory for Palygorskite Science and Applied Technology of Jiangsu Province, Huaiyin Institute of Technology, Huai’an, 223003 China

**Keywords:** *Panus rudis*, Solid-state fermentation, Benzothiazole derivatives, Cytotoxicity

## Abstract

A chemical constituent study on the fermented rice substrate of basidiomycetous fungus *Panus rudis* led to the isolation of four previously undescribed prenylhydroquinone derivatives compounds (**1**–**4**) and eight known compounds (**5**–**12**). Among them, compound **3** featured a rare benzothiazole derivative with hydroxy substituted 3-methyl-1-butenyl substitution on the benzene ring, and the absolute configurations of **7** and **12** were elucidated as unreported ones. Their structures were identified by the interpretation of 1D and 2D NMR spectroscopy, HRESIMS data, X-ray single-crystal diffraction, and comparison of calculated and experimental ECD spectra. The plausible biosynthetic pathways for **1**–**7** are proposed. Cytotoxicity evaluation was conducted on **1** and **3**–**12** against two cancer cell lines (A-549 and HepG2). The results demonstrated that **1**, **3**–**6**, **8**, **9**, and **12** exhibited weak cytotoxicity against both two cell lines. Among them, **8** and **12** showed dose-dependent inhibitory effects, and their IC_50_ values at 72 h were obtained.

**Graphical Abstract:**

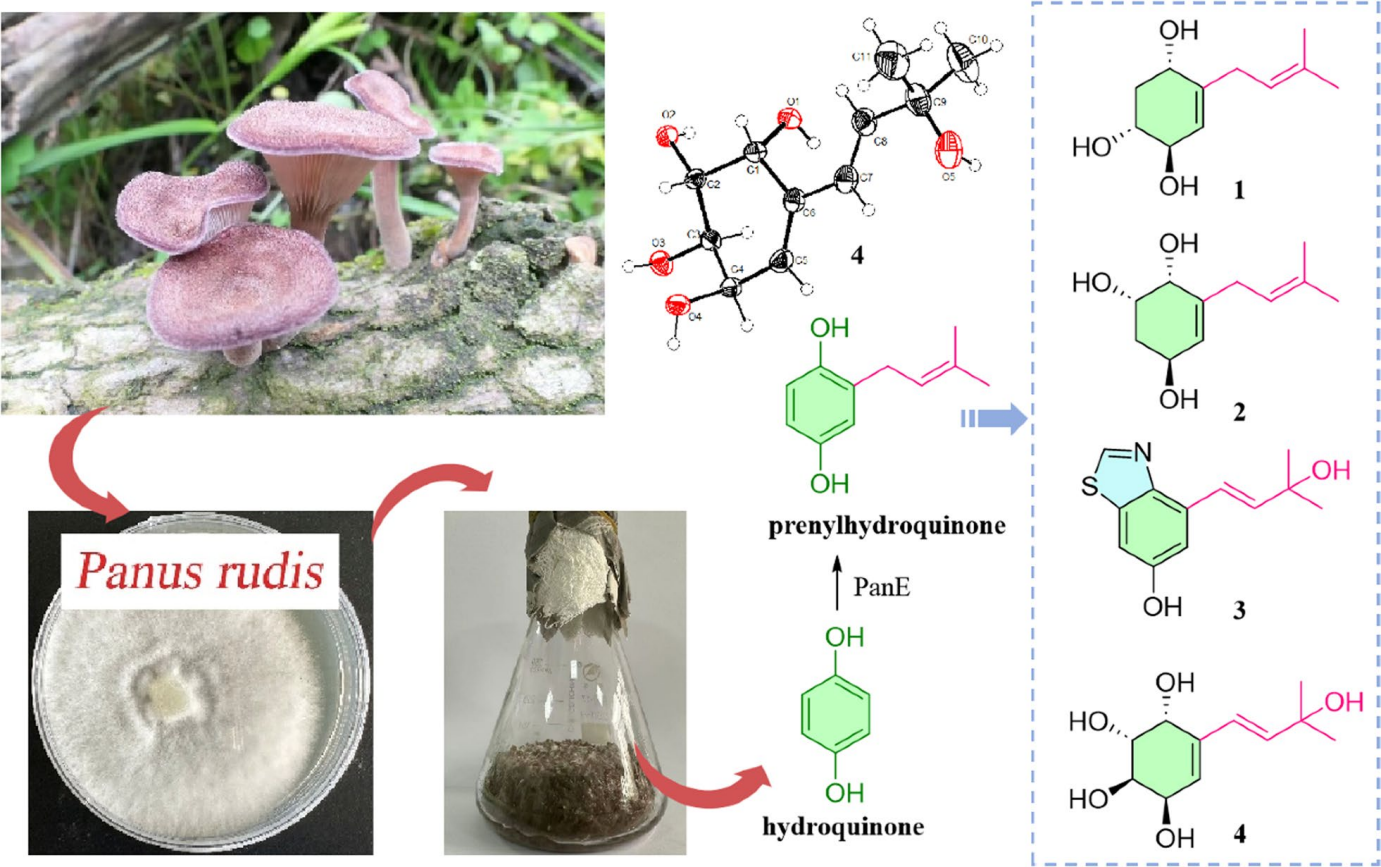

**Supplementary Information:**

The online version contains supplementary material available at 10.1007/s13659-025-00562-3.

## Introduction

*Panus rudis* (*Polyporales*, *Panaceae*) shows remarkable economic and ecological value by secretion of lignin-modifying enzymes (e.g., laccase) [1–3]. And it is a lignicolous fungus belonging to Basidiomycota, which comprises most mushroom-type fungi and is recognized as an ingenious producer of highly functionalized natural products and a rich source of biosynthetic enzymes [4]. Panepoxydone, biosynthesized from prenylhydroquinone through successive hydroxylation, epoxidation, and reduction reactions, was first isolated from *P. rudis*, which exhibited multiple biological activities, such as inhibitory activity against NF-*κ*B as well as antitumor activity [5]. Afterwards, a series of panepoxydone analogues were obtained through liquid fermentation or precursor-directed biosynthesis by *P. rudis* [6–8]. Hexacyclinol, a dimeric prenylhydroquinone isolated from this genus, had been highly controversial. Its accurate structure was ultimately established through the calculation of the^13^C-NMR chemical shifts. Subsequently, Porco et al. completed the total synthesis of the reassigned (+)-hexacyclinol and confirmed its absolute configuration via X-ray crystallography [9–11]. In recent years, structurally diverse dimers and their respective monomeric intermediates had even been proven to be produced by *P. rudis*, using 5-FAM-maleimide combined with UHPLC–MS/MS–FBMN workflow. And dimers with four types of complexes and densely functionalized cores were identified from this fungus [12]. Moreover, abundant secondary metabolites also have been reported from the fungi of *Panus* [13–15]. Recently, four racemic cyclohexene dimers and two non-racemic cyclohexene monomers were isolated from *P. similis* [16]. Among them, (3R*,4S*)-2,2-Dimethyl-3,4,6-trihydroxychromane exhibited selective cytotoxicity towards NCI-H187 cells (IC_50_ = 9.1 *μ*M). However, most *Panus* fungi had been investigated for their fermentation broths, but the metabolic potential of *P. rudis* within solid-state fermentation systems remains to be further explored. Solid-state fermentation, as a time-honored bioprocessing technology, plays an indispensable role in the production of characteristic fermented foods which not only confers distinctive flavors and textures to food products but also biosynthesizes functional secondary metabolites [17–20]. And rice as a solid-state fermentation medium shows the great potential of discovering more natural metabolites with diverse structures or significant bioactivities [21–23].

**Fig. 1:**
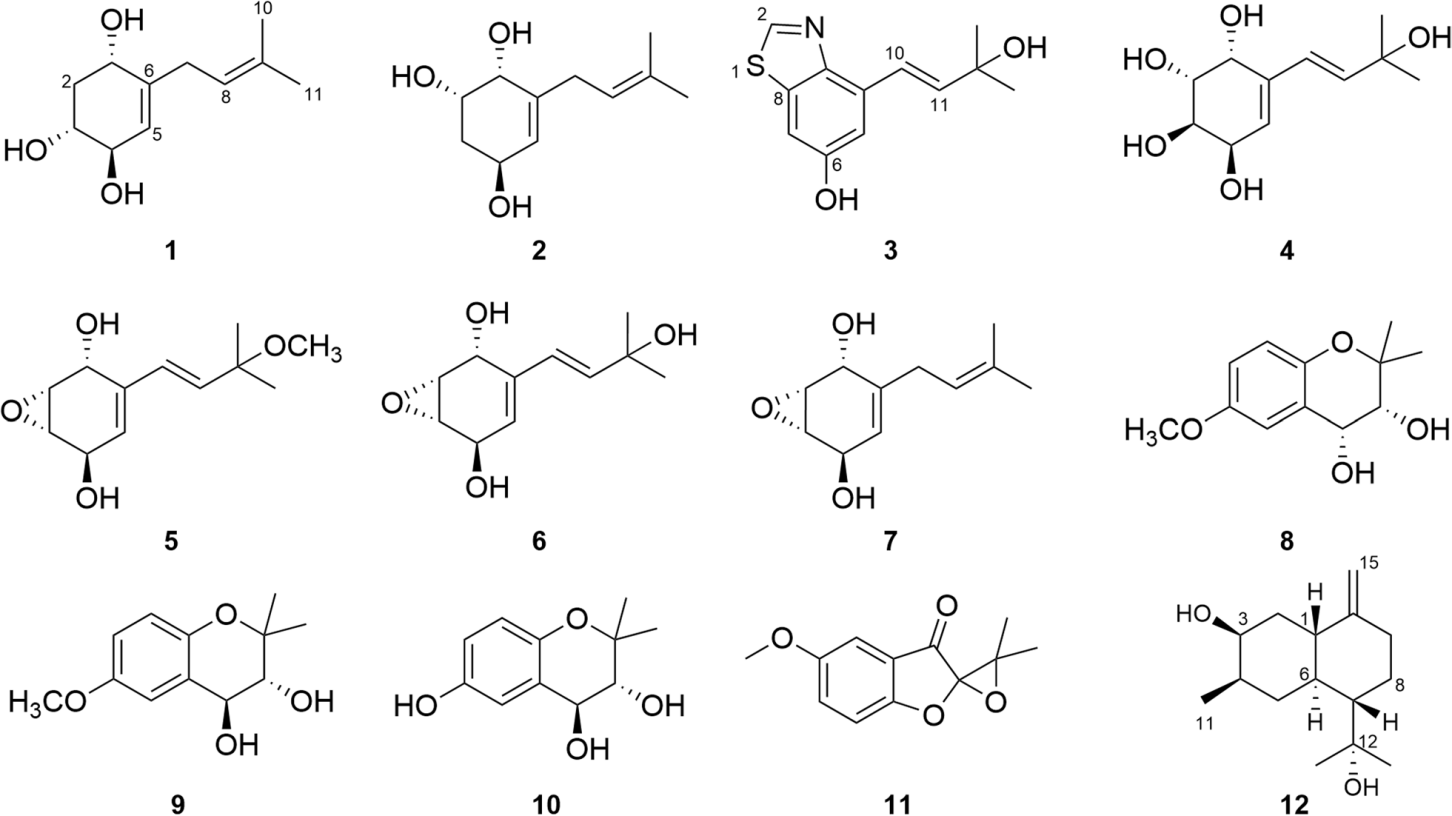
The structures of compounds **1**–**12**

This study investigating the rice-based solid-state fermentation metabolites of *P. rudis* by employing multifaceted chromatographic separation coupled with spectroscopic characterization techniques resulted in the isolation of a series of prenylhydroquinone derivatives (Fig. 1), and the plausible biosynthetic pathway of them was inferred. In order to screen compounds with potential for biological activities, the cytotoxicity of the isolates was also tested.

## Results and discussion

**Fig. 2:**
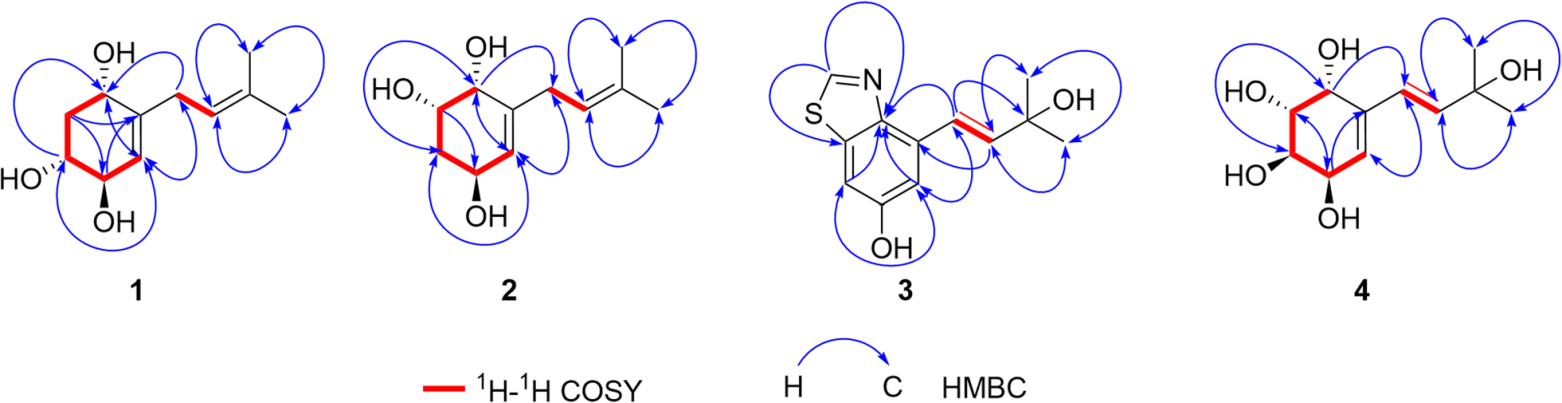
Key ^1^H-^1^H COSY and HMBC correlations of compounds **1**–**4**

Compound **1** was obtained as a white amorphous powder, and its molecular formula was determined as C_11_H_18_O_3_ by means of HR-ESI–MS (*m*/*z* 221.1151 [M + Na]^+^, calcd for C_11_H_18_O_3_Na^+^, *m*/*z* 221.1154). The ^1^H NMR spectrum data (Table 1) indicated the presence of two olefinic methines at *δ*_H_ 5.30 (m, 1H) and 5.19 (m, 1H), three oxygenated methines at *δ*_H_ 4.19 (m, 1H), 3.98 (m, 1H) and 3.49 (ddd, *J* = 11.8, 7.5, 3.4 Hz, 1H), two methylene at *δ*_H_ 2.81 (m, 2H), 2.23 (ddd, *J* = 12.3, 5.7, 3.5 Hz, 1H) and 1.60 (overlapped, 1H), and two methyl groups at *δ*_H_ 1.73 (s, 3H) and 1.63 (s, 3H). ^13^C NMR (Table 1) and HSQC spectrum revealed the signals of 11 carbons, including two double bonds (*δ*_C_ 143.5, 134.4, 126.0 and 122.6), three oxygenated methines (*δ*_C_ 74.3, 73.2 and 69.3), two methylenes (*δ*_C_ 40.5 and 31.7), and two methyls (*δ*_C_ 17.9 and 26.1). Two structural fragments (C-1/C-2/C-3/C-4/C-5 and C-7/C-8) can be readily deduced on the basis of ^1^H–^1^H COSY spectrum (Fig. 2). The HMBC correlations (Fig. 2) between the two methyl groups and the olefinic methine indicate the presence of an isopentenyl moiety. According to the HMBC cross-peaks from H-5 to C-3/C-1, from H-2 to C-4/C-6, and from H-1 to C-5, combined with ^1^H–^1^H COSY correlations (H-1/H-2/H-3/H-4/H-5), a cyclohexene fragment with hydroxyl groups can be constructed. The connectivity of the above two fragments through C-6 and C-7 was established based on the HMBC correlations from H-7 to C-1/C-5 and from H-5 to C-7. Based on the aforementioned NMR data, the planar structure of **1** was characterized (Fig. 1).

**Table 1:** ^1^H and ^13^C NMR Data of Compounds **1**–**4**

No.	**1**^a^		**2**^a^		**4**			**3**^a^	
	*δ*_C_, type	*δ*_H_, mult (*J* in Hz)	*δ*_C_, type	*δ*_H_, mult (*J* in Hz)	*δ*_C_^b^, type	*δ*_H_^b^, mult (*J* in Hz)	*δ*_H_^c^, mult (*J* in Hz)	*δ*_C_, type	*δ*_H_, mult (*J* in Hz)
1	69.3, CH	4.19, m	69.8, CH	3.90, d (3.7)	66.3, CH	4.17, dd (5.3, 3.8)	4.42, d (4.0),		
2	40.5, CH_2_	2.23, ddd (12.3, 5.7, 3.5), 1.60, overlapped	68.1, CH	3.96, dt (10.7, 3.5)	68.4, CH	3.60, dt (9.1, 3.9)	3.77, dd (10.5, 4.0)	152.3, CH	8.92, s
3	73.2, CH	3.49, ddd (11.8, 7.5, 3.4)	35.6, CH_2_	2.04, m, 1.67, overlapped	69.0, CH	3.67, dt (9.2, 4.4)	3.85, dd (10.5, 4.4)		
4	74.3, CH	3.98, m	66.0, CH	4.27, m	66.6, CH	4.08, dd (8.7, 4.4)	4.27, (4.8)	134.0, C	
5	126.0, CH	5.30, m	127.0, CH	5.54, d (4.4)	125.7, CH	5.60, d (4.8)	5.8, d (5.1)	113.2, CH	7.15, d (2.3)
6	143.5, C		141.6, C		137.3, C			157.6, C	
7	31.7, CH_2_	2.81, m	33.8, CH_2_	2.85, m	128.5, CH	6.02, s	6.20, d (16.1)	106.9, CH	7.25, d (2.3)
8	122.6, CH	5.19, m	122.5, CH	5.19, m	139.5, CH	6.02, s	6.12, d (16.1)	137.1, C	
9	134.4, C		134.7, C		69.2, C			146.3, C	
10	17.9, CH_3_	1.63, s	17.9, CH_3_	1.65, s	30.2, CH_3_	1.18, s	1.31 (s, 3H),	123.7, CH	7.30, d (16.2)
11	26.1, CH_3_	1.73, s	26.0, CH_3_	1.73, s	30.1, CH_3_	1.18, s	1.30 (s, 3H)	141.9, CH	6.70, d (16.2)
12								71.8, C	
13								30.0, CH_3_	1.44, s
14								30.0, CH_3_	1.44, s

^a^Recorded ^1^H (400 MHz) and ^13^C NMR (101 MHz) in CD_3_OD. ^b^Recorded ^1^H (400 MHz) and ^13^C NMR (101 MHz) in DMSO-*d*_6_. ^c^Recorded ^1^H (600 MHz) in CD_3_OD

**Fig. 3:**
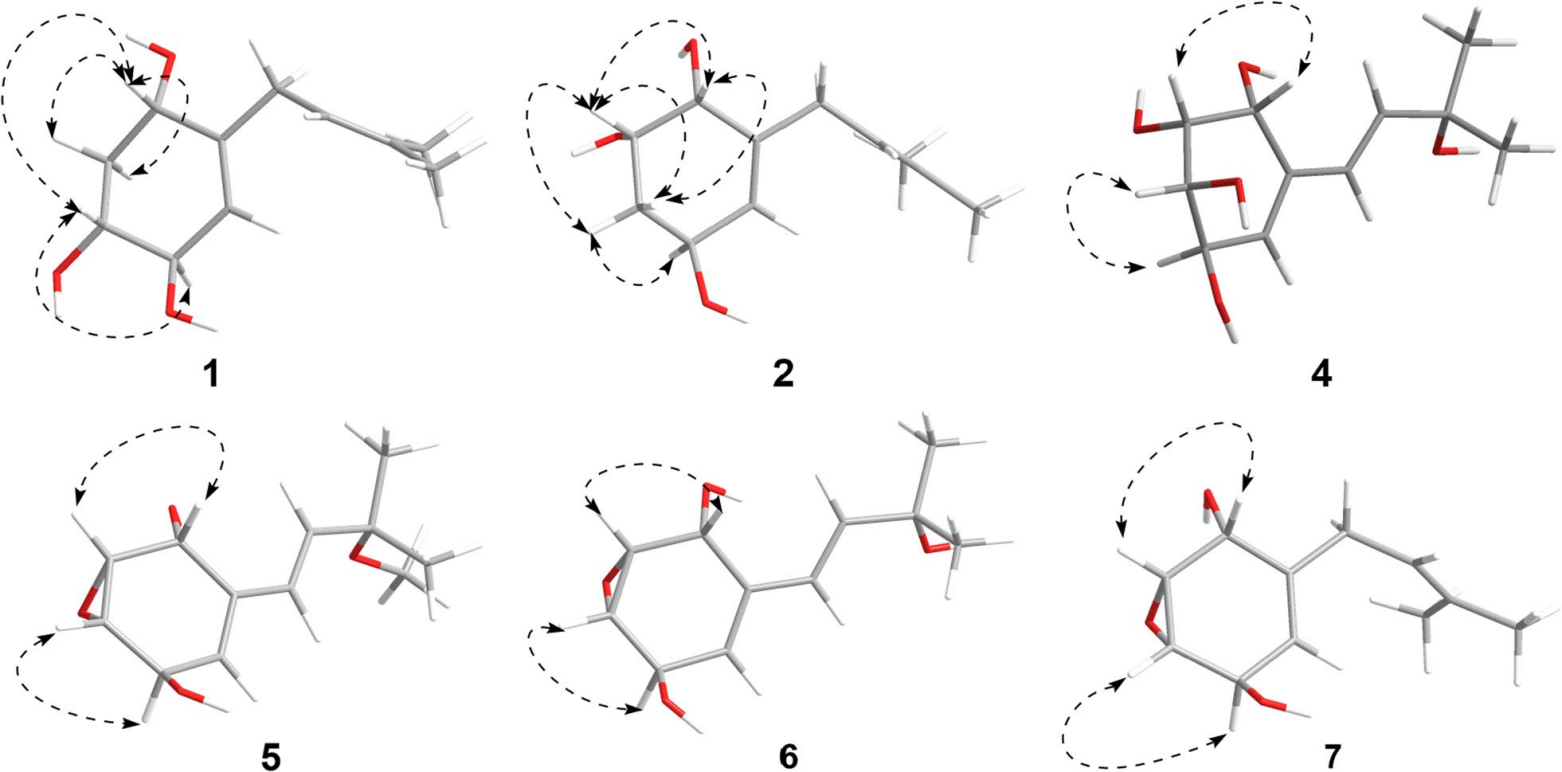
Key NOESY correlations of compounds **1**, **2**, and **5**–**7** and key ROESY correlations of compound **4**

**Fig. 4:**
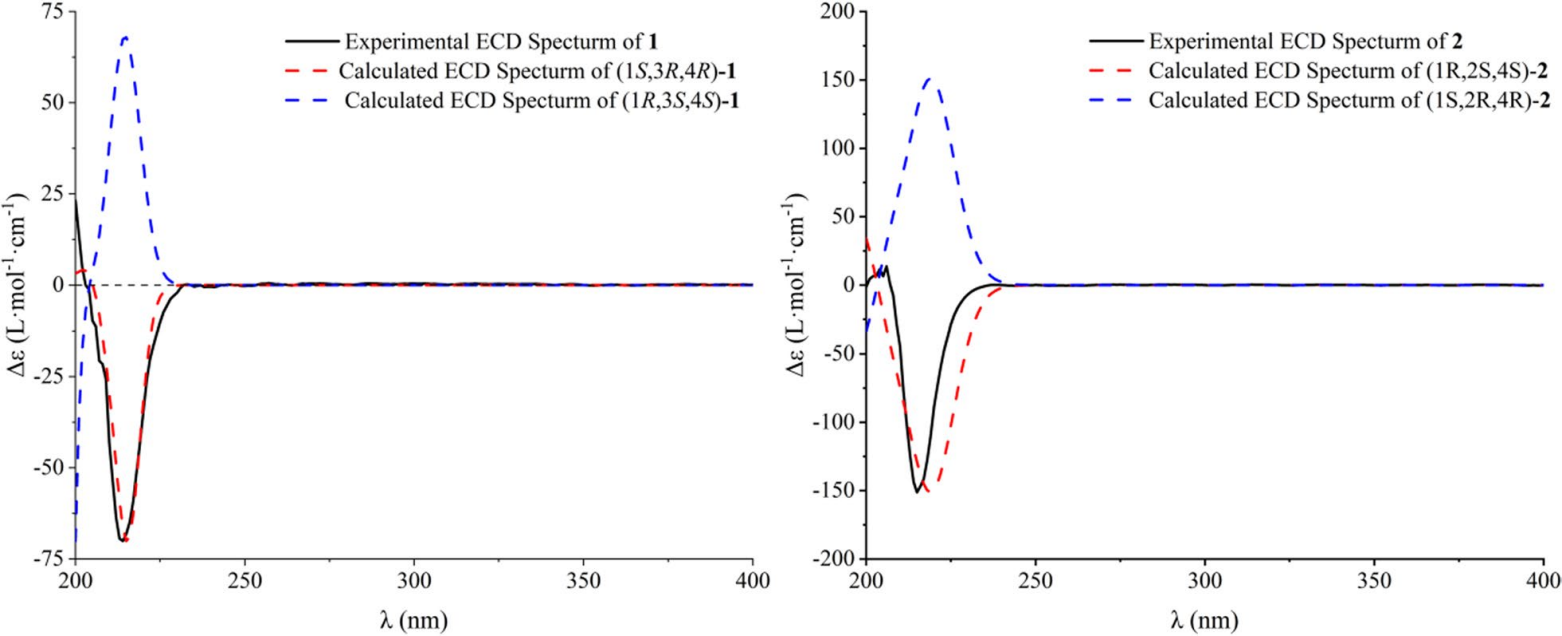
Experimental and calculated ECD spectra of compounds **1** and **2**

The NOESY correlations (Fig. 3) of H-1 with H-3, which indicated that H-1 and H-3 exhibit a *cis*-configuration. By further interpreting the multiplicities of H-3 (*J*_3,2a_ = 11.8, *J*_3,2e_ = 3.4, *J*_3,4_ = 7.5), the anti-orientation of H-3/H-4 was suggested by a large *J*_3,4_ (7.5 Hz). Thus, the relative configuration of **1** can be determined as shown in Fig. 3. Its absolute configuration was speculated to be 1*S*,3*R*,4*R* upon comparing its experimental ECD curve (Fig. 4) with the calculated ones, and **1** was named as panunoid A.

Compound **2** was obtained as a white amorphous powder, and its molecular formula was determined as C_11_H_18_O_3_ the same with **1**, by means of HR-ESI–MS (*m*/*z* 199.1326 [M + H]^+^, calcd for C_11_H_19_O_3_^+^, *m*/*z* 199.1334). The ^1^H and ^13^C NMR data of **2** (Table 1) closely resembled those of **1**. The differences in carbon chemical shifts were observed at the hydroxyl-substituted cyclohexene moieties, indicating that they could be isomers differing in the positions or configurations of the hydroxyl groups. The planar structure of **2** was further confirmed through 2D NMR spectra (Fig. 2).

The NOESY correlations (H-1/H-2) and the small *J*_1,2_ (3.5) of compound **2** suggested the *cis*-orientation of H-1/H-2. By comparing with **1**, no correlation between H-2 and H-4 was observed, it can be presumed that H-2 and H-4 are arranged in a trans configuration. Thus, the relative configuration of **2** could be speculated as shown in Fig. 3. The possible absolute configuration was supposed to be 1*R*,2*S*,4*S* upon comparing its experimental ECD curve (Fig. 4) with the calculated ones and **2** (Fig. 1) was named as panunoid B.

Compound **3** was obtained as a brown oil, and its molecular formula was determined as C_12_H_13_NO_2_S by means of HR-ESI–MS (*m*/*z* 234.0596 [M–H]^–^, calcd for C_12_H_12_NO_2_S^–^, *m*/*z* 234.0589; *m*/*z* 218.0644 [M–H_2_O + H]^+^, calcd for C_12_H_12_NOS^+^, *m*/*z* 218.0640). The ^1^H NMR spectrum data (Table 1) indicated the presence of two coupled protons on the double bond at *δ*_H_ 7.30 (d, *J* = 16.2 Hz, 1H) and 6.70 (d, *J* = 16.2 Hz, 1H), along with two coupled aromatic protons [*δ*_H_ 7.25 (d, *J* = 2.3 Hz, 1H) and 7.15 (d, *J* = 2.3 Hz, 1H)] at the meta-position on the benzene ring. Additionally, a deshielded aromatic proton signal was observed at *δ*_H_ 8.93 (s, 1H), which may be attributed to an aromatic heterocyclic group. Moreover, two methyl groups in the same chemical environment at *δ*_H_ 1.44 (s, 6H) were observed in ^1^H NMR spectrum. The ^13^C NMR (Table 1) and HSQC spectrum data revealed the signals of 12 carbons, including an oxygenated quaternary carbon at *δ*_C_ 71.8, a double bond at *δ*_C_ 141.9 and 123.7, two methyl carbons resonating at *δ*_C_ 30.0, along with other seven aromatic carbons distributed between *δ*_C_ 157.6 and 106.9.

Based on the HMBC correlations from H-2 to C-8/C-9, H-5 to C-7/C-9, and from H-7 to C-9/C-5 combined with its molecular formula, the partial structure of 6-hydroxybenzothiazole could be obtained, which was also reinforced by comparing with the NMR data reported in the literature [24]. Furthermore, the correlations from H-10 to C-5/C-9/C-12, H-5 to C-10, H-11 to C-4, and H-13 to C-11 indicated that a hydroxy substituted 3-methyl-1-butenyl was connected with C-4. In summary, the structure of **3** can be obtained as drawn in Fig. 1 and named as panunoid C.

Compound **4** was obtained as a colorless crystal. The ^1^H NMR spectra were recorded both in CD_3_OD and DMSO-*d*_6_ (Table 1), and the presence of hydroxyl signals resulted in more complex coupling patterns for hydrogen signals recorded in DMSO-*d*_6_ than those recorded in CD_3_OD. The ^1^H NMR spectrum recorded in CD_3_OD showed the presence of an olefinic methine at 5.60 (d, *J* = 4.8 Hz, 1H), two trans-coupled hydrogens on the double at *δ*_H_ 6.20 (d, *J* = 16.1 Hz, 1H) and 6.12 (d, *J* = 16.1 Hz, 1H), four oxygenated methines at *δ*_H_ 4.42 (d, *J* = 4.0 Hz, 1H), 4.27 (d, *J* = 4.8 Hz, 1H), 3.85 (dd, *J* = 10.5, 4.4 Hz, 1H) and 3.77 (dd, *J* = 10.5, 4.0 Hz, 1H), and two methyls at *δ*_H_ 1.31 (s, 3H) and 1.30 (s, 3H). ^13^C NMR (Table 1) and HSQC spectra revealed the signals of 11 carbons, including two double bonds at *δ*_C_ 137.3, 139.5, 128.5 and 125.7, four oxygenated methine carbons at *δ*_C_ 69.0, 68.4, 66.6 and 66.3, an oxygenated quaternary carbon at *δ*_C_ 69.2, and two methyls at *δ*_C_ 30.2 and 30.1, respectively.

**Fig. 5:**
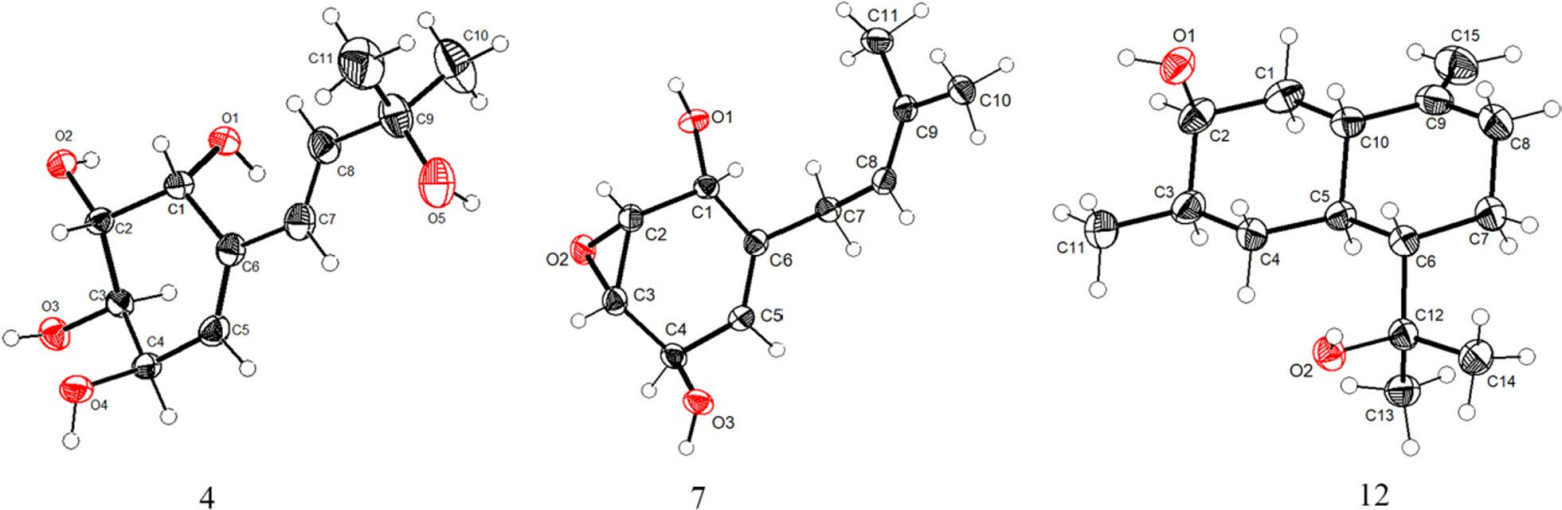
ORTEP drawing of **4**, **7**, and **12** obtained by X-ray analysis

The ^1^H–^1^H COSY spectrum (Fig. 2) determined two partial structures of C-1/C-2/C-3/C-4/C-5 and C-7/C-8, which showed similarity to those of **1**. Then the planar structure of **4** was established as shown in Fig. 1 through HMBC correlations from H-10/H-11 to C-8, H-8 to C-10/C-11, H-7 to C-5, H-5 to C-7, and H-1 to C-7/C-3. The NOESY cross-peaks of H-1/H-2 and H-3/H-4 (Fig. 3) together with the small *J*_1,2_ (4.0) and *J*_3,4_ (4.4), and the big *J*_2,3_ (10.5) revealed that H-1 and H-2 were on the same side of the ring, so were H-3 and H-4. Furthermore, the absolute configuration of **4** (Deposition No. CCDC-2417279) was determined by single-crystal X-ray diffraction (Cu K*α*) with a Flack parameter 0.03(4) (Fig. 5) and named as panunoid D.

Molecular formula of compound **7** was determined as C_11_H_16_O_3_ by means of HR-ESI–MS (*m*/*z* 197.1174 [M + H]^+^, calcd for C_11_H_17_O_3_^+^, *m*/*z* 197.1178). The ^1^H NMR and ^13^C NMR data see Table 2. The planar structure, as determined by 1D and 2D NMR spectroscopy, was consistent with that of 7-desoxyphenepoxydol [25]. The NOESY spectrum (Fig. 3) revealed correlations of H-1/H-2 and H-3/H-4, with the cross-peak intensity of H-1/H-2 being significantly stronger than that of H-3/H-4. Analogous characteristics of intensity of cross-peaks (H-1/H-2 and H-3/H-4) were also observed in the ^1^H–^1^H COSY spectrum. This observation suggested a *cis*-configuration between H-1 and H-2. Combining with the above-mentioned information and the absence of correlation between H-1 and H-4 suggested that the epoxide and hydroxyl group of C-1 were syn-oriented and the epoxide and hydroxyl group of C-4 were anti-oriented. By single-crystal X-ray diffraction (Cu K*α*) with a Flack parameter 0.04(13) (Fig. 5), its absolute configuration was determined as 1*R*,2*R*,3*S*,4*R*.

**Table 2:** ^1^H and ^13^C NMR Data of Compounds **5**–**7**

No.	**5**		**6**		**7**	
	*δ*_C_, type	*δ*_H_, mult (*J* in Hz)	*δ*_C_, type	*δ*_H_, mult (*J* in Hz)	*δ*_C_, type	*δ*_H_, mult (*J* in Hz)
1	65.4, CH	4.62, dt (3.2, 1.5)	65.4, CH	4.61, dt (3.2, 1.5)	66.9, CH	4.36, dt (3.1, 1.7)
2	56.2, CH	3.50, t (3.5)	56.2, CH	3.50, t (3.6)	56.2, CH	3.42, t (3.5)
3	57.3, CH	3.38, ddd (3.7, 2.1, 1.4)	57.3, CH	3.38, ddd (3.7, 2.2, 1.5)	57.0, CH	3.32 ddd (3.9, 2.2, 1.5)
4	64.0, CH	4.36, d (4.7)	64.0, CH	4.36, d (4.6)	64.2, CH	4.25, d (4.5)
5	126.7, CH	5.71, m	126.1, CH	5.68, m	122.6, CH	5.37, m
6	136.5, C		136.6, C		139.5, C	
7	130.0, CH	6.12, d (16.4)	126.6, CH	6.15, d (16.1)	32.2, CH_2_	2.83, m
8	138.1, CH	6.07, d (16.4)	141.0, CH	6.24, d (16.1)	122.3, CH	5.17, m
9	76.8, C		71.5, C		134.8	
10	26.5, CH_3_	1.29, s	30.0, CH_3_	1.30, s	17.9, CH_3_	1.62, s
11	26.0, CH_3_	1.28, s	30.0, CH_3_	1.30, s	26.1, CH_3_	1.72, s
	50.9, OCH_3_	3.16, s				

Recorded ^1^H (400 MHz) and ^13^C NMR (101 MHz) in CD_3_OD

The molecular formula of **5** and **6** was determined as C_12_H_18_O_4_ and C_11_H_16_O_4_ by means of HR-ESI–MS (*m*/*z* 249.1119 [M + Na]^+^, calcd for C_12_H_18_NaO_4_^+^, *m*/*z* 249.1097 and *m*/*z* 235.1056 [M + Na]^+^, calcd for C_11_H_16_NaO_4_^+^, *m*/*z* 235.0941), respectively. The ^1^H NMR and ^13^C NMR data (Table 2) of **5** were consistent with panepoxydiol [6]. The NMR data (Table 2) of **6** were similar to those of **5**, except that the methoxy group (C-9) was replaced by a hydroxyl group, thus the planar structure of **6** was the same with (*E*)-3-(3-hydroxy-3-methylbut-1-en-1-yl)-7-oxabicyclo[4.1.0]hept-3-ene-2,5-diol [6, 26]. Lentinoid B was previously reported with the same planar structure of **4** but the same spectra data with **6** [27]. The structural revision of the lentinoid B into compound **6** was determined based on its chemical shift of C-2 (*δ*_C_ 57.1) and C-3 (*δ*_C_ 56.0) being upfield-shifted compared to compound **4** (C-2 *δ*_C_ 68.4, C-3 *δ*_C_ 69.0) and resembling that of other epoxide analogues. The correlations and the features of cross-peak intensity in NOESY and COSY spectra for both **5** and **6** were consistent with those of **7**. The peak shapes and coupling constants in the ^1^H NMR spectrum (Table 2) of **5**, **6** and **7** are identical. Consistency between spectroscopic data and biosynthetic logic suggested that compounds **5**, **6**, and **7** shared an identical stereochemical configuration (Fig. 1).

The other known compounds were identified as (3*R*,4*R*)-3,4-dihydroxy-6-methoxy-2,2-dimethylchroman (**8**) [28], (3*R*,4*S*)-3,4-dihydroxy-6-methoxy-2,2-dimethylchroman (**9**) [29], (3*R*,4*S*)-3,4,6-dihydroxy-2,2-dimethyl-chroman (**10**) [30], 5-Methoxy-3′,3′-dimethyl-3*H*-spiro[benzofuran-2,2′-oxiran]-3-one (**11**) [15] by comparison of NMR spectroscopic data with published literature values. Among them, **11** might be a racemic mixture. (4*S*)-3*α*,12-Dihydroxycadin-10(15)-ene previously obtained by biotransformation of cadina-4,10(15)-dien-3-one, a substrate added exogenously in the culture medium, by the fungi *Mucor plumbeus* ATCC 4740 [31] shared the same planar structure with **12**. In this work, its absolute configuration was determined as 1*S*,3*S*,4*R*,6*S*,7*S* by single-crystal X-ray diffraction (Cu K*α*) with a Flack parameter 0.04(4) (Fig. 5).

**Fig. 6:**
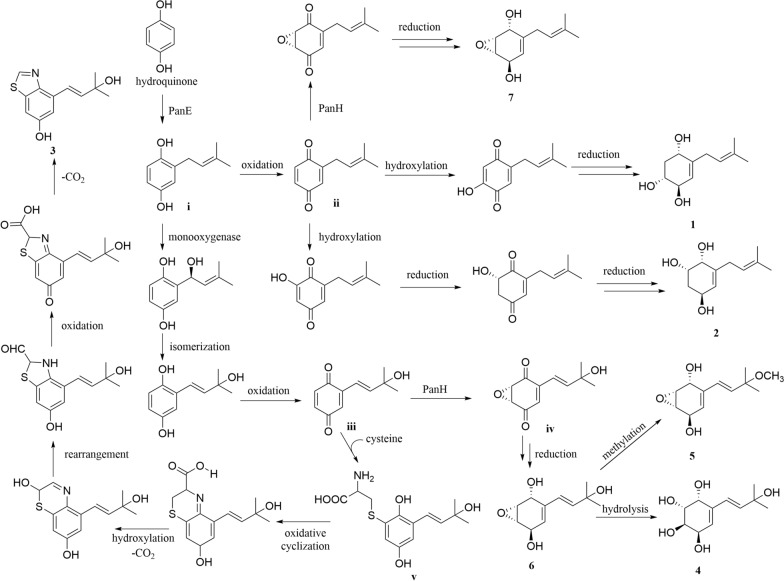
The plausible biosynthetic pathway for compounds **1**–**7**

The plausible biosynthetic pathway for compounds **1**–**7** (Fig. 6) was proposed starting from hydroquinone [5]. The premise of the whole process is to modify hydroquinone by PanE to obtain intermediate **i**. Briefly, compounds **1** and **2** were derived from intermediate **i** by oxidation, hydroxylation, and reduction, respectively [32]. The intermediate **ii** was catalytically epoxidated by PanH and subsequently reduced to obtain compound **7**. What is more, intermediate **i** was modified by monooxygenase, isomerized and oxidized to produce intermediate **iii**. The condensation of cysteine with intermediate **iii** afforded intermediate **v**. Compound **3** was obtained from intermediate **v** through a series of steps [33, 34]. Intermediate **iv** was obtained by catalytic epoxidation of intermediate **iii** via PanH. Intermediate **iv** was reduced to give compound **6**. In addition, compounds **5** and **4** were obtained from compound **6** through methoxylation and hydrolysis, respectively.

**Fig. 7:**
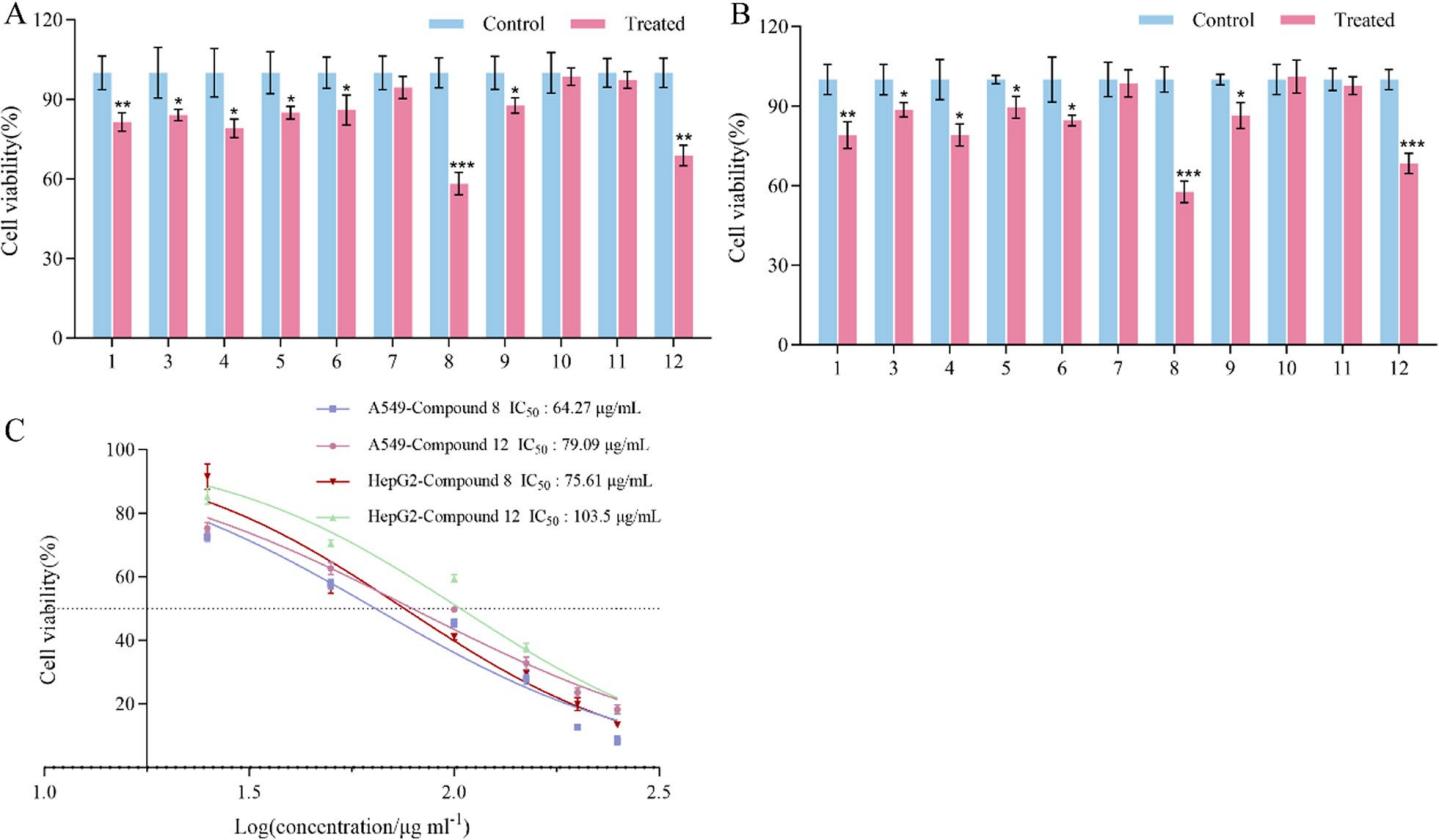
Effect on cell viability of A-549 and HepG2 in vitro. **A**. Cell viability of A-549 after 72 h of drug treatment (concentration: 50 *μ*g/mL); **B**. Cell viability of HepG2 after 72 h of drug treatment (concentration: 50 *μ*g/mL); and **C**. IC_50_ values after 72 h of compound treatment (concentrations: 250, 200, 150, 100, 50, and 25 *μ*g/mL). Note: *P < 0.05, **P < 0.01, ***P < 0.001

All compounds were subjected to cytotoxicity testing, except for **2**, which had a small amount. The in vitro activity results revealed that **1**, **3**–**6**, **8**, **9**, and **12** exhibited cytotoxic effects against A-549 and HepG2 cell lines (Fig. 7). Among them, **8** and **12** have stronger cell viability inhibitory effects on A-549 and HepG2 cells, and show a dose-dependent effect (Fig. 7). Compound **8** has an IC_50_ value of 64.27 *μ*g/mL against A-549 cells and 75.61 *μ*g/mL against HepG2 cells. Compound **12** has an IC_50_ value of 79.09 *μ*g/mL against A549 and 103.5 *μ*g/mL against HepG2.

## Experimental

### General

A KRÜSS P8000 (KRÜSS GmbH, Hamburg, Germany) automatic polarimeter were used to measure specific rotations. A MOS-500 (Konica Minolta Sensing, Inc., Osaka, Japan) spectrometer was used to measure CD spectra. HR-ESI–MS was determined using Acquity UPLC I-class plus tandem Xevo G2-XS Qtof (Waters Co., Milford, MA, USA). Single crystal X-ray structure was obtained on Bruker D8 Venture (Bruker AXS GmbH, Karlsruhe, Germany). 1D and 2D NMR spectra were measured on Bruker Avance-400 and Avance-600 (Bruker BioSpin GmbH, Germany) spectrometers with CD_3_OD and DMSO-*d*_6_ as solvents and TMS as internal standard. Column chromatography (CC) was carried out over silica gel (200–300 mesh) (Qingdao Haiyang Chemical Co., Qingdao, China). Pre-coated silica gel plates (Qingdao Haiyang Chemical Co., Qingdao, China) were used for thin-layer chromatography (TLC). Detection was done under UV light (254 and 365 nm) and by spraying the plates with 10% sulfuric acid ethanol solution followed by heating. An Agilent series 1260 (Agilent Technologies, Santa Clara, CA, USA) was used for analysis HPLC. Semi-preparative HPLC was done on a Hanbon series NP7005C (Hanbon Sci & Tech, Jiangsu, China), the column used was HB-C18FP-100-9 (9 *μ*m, 100Å, 20 × 250 mm, Hanbon Sci & Tech, Jiangsu, China). A CHIRALPAK^®^ AD-H column (5 *μ*m, 4.6 × 250 mm, Daicel Corporation, Japan) was employed for chiral compound separation.

### Fungi culture

*P. rudis* strain CGMCC 5.34 was purchased from China General Microbiological Culture Collection Center, and stored in Jiangsu Key Laboratory of Regional Specific Resource Pharmaceutical Transformation. The strain was cultured on YMG solid medium and incubated at 28 °C for mycelia production. Subsequently, the mycelia were aseptically transferred into conical flasks containing YMG liquid medium. These were incubated as seed cultures for 7 days on a rotary shaker at 130 rpm (28 °C). For large-scale cultivation, thirty 500 mL conical flasks were prepared, each containing 100 g rice supplemented with 100 mL purified water. Each flask was inoculated with 10 mL of seed medium and incubated statically at 28 °C for 40 days. The fermented rice substrate was subjected to methanol for extraction (3 × 20 L × 3 days). After combining the extracts, the organic solvent was removed via rotary evaporation under vacuum, yielding 2.4 L of concentrated aqueous solution. This solution was extracted three times with ethyl acetate, and the combined organic phases were concentrated to dryness under vacuum, ultimately obtaining 12.76 g of crude extract.

### Extraction and isolation

The crude extract (12.76 g) was submitted to silica gel (200–300 mesh) column chromatography (CC), eluting with petroleum ether–EtOAc gradient system (100:0, 50:1, 30:1, 20:1, 10:1, 8:1, 5:1, 1:1, v/v), and then eluting with CH_2_Cl_2_–MeOH gradient system (20:1, 15:1, 10:1, 9:1, 8:2, 7:3, 6:4, 1:1, 0:100, v/v), to yield nine fractions (L1–L9).

L4 (3.3 g) was applied to silica gel (200–300 mesh) CC, and eluting with petroleum ether–EtOAc gradient system (100:0, 30:1, 20:1, 10:1, 8:1, 5:1, 1:1, v/v) and CH_2_Cl_2_–MeOH (20:1, 15:1, 10:1, 9:1, 8:2, 7:3, 6:4, 1:1, 0:100, v/v) to yield nine fractions (L41–L49). L46 (183 mg) was further purified through semi-preparative HPLC (10–70% MeCN, 60 min, flow rate 15 mL/min) to yield **11** (*t*_*R*_ of 31.35 min, 6.1 mg). L47 and L48 were combined and named as L47. The fraction L47 (267 mg) underwent purification via semi-preparative HPLC (15–75% MeCN, 60 min, flow rate 15 mL/min), which yielded nine sequentially eluted fractions (L471–L479) based on retention time. L476 (175 mg) was further purified through semi-preparative HPLC (45% MeCN, flow rate 15 mL/min) to yield **12** (*t*_*R*_ of 17.14 min, 35.5 mg).

L5 (4.2 g) was purified by semi-preparative HPLC (10–37% MeCN, 80 min, flow rate 15 mL/min) to yield **3** (*t*_*R*_ of 50.42 min, 28.6 mg), **6** (*t*_*R*_ of 6.86 min, 33.8 mg), **7** (*t*_*R*_ of 30.93 min, 358.6 mg), L52, L53, L58 and L510. L52 and L53 were combined and named as L52. L52 (40 mg) was further purified through semi-preparative HPLC (10% MeOH, flow rate 15 mL/min) to yield **5** (*t*_*R*_ of 9.71 min, 5.4 mg). L58 (41.2 mg) was further purified by semi-preparative HPLC (37% MeOH, flow rate 15 mL/min) to yield **8** (*t*_*R*_ of 33.75 min, 8.2 mg). L510 (139 mg) was further purified through semi-preparative HPLC (40% MeOH, flow rate 15 mL/min) to yield **9** (*t*_*R*_ of 31.69 min, 85.9 mg).

L6 (1.8 g) was further separated by semi-preparative HPLC (5–75% MeOH, 60 min, flow rate 15 mL/min) to yield **1** (*t*_*R*_ of 36.47 min, 54 mg), **4** (*t*_*R*_ of 14.55 min, 552.1 mg), **10** (*t*_*R*_ of 32.62 min, 28 mg) and L68. L68 (13 mg) was further purified by preparative HPLC with a DAICEL AD column (n-Hexane: EtOH = 9: 1, flow rate 1 mL/min) to yield **2** (*t*_*R*_ of 9.32 min, 4 mg).

### Compound characterization

Panunoid A (**1**). White amorphous powder. $\left[ \alpha \right]_{{\text{D}}}^{20}$ − 131.8 (*c* 0.13, MeOH); ^1^H and ^13^C NMR spectroscopic data see Table 1; HR-ESI–MS *m*/*z* 221.1151 [M + Na]^+^ (calculated for C_11_H_18_O_3_Na^+^, *m*/*z* 221.1154).

panunoid B (**2**). White amorphous powder. $\left[ \alpha \right]_{{\text{D}}}^{20}$ − 227.2 (*c* 0.14, MeOH); ^1^H and ^13^C NMR spectroscopic data see Table 1; HR-ESI–MS *m*/*z* 199.1326 [M + H]^+^ (calculated for C_11_H_19_O_3_^+^, *m*/*z* 199.1334).

panunoid C (**3**). Brown oil. $\left[ \alpha \right]_{{\text{D}}}^{20}$ − 108.8 (*c* 0.12, MeOH); ^1^H and ^13^C NMR spectroscopic data see Table 1; HR-ESI–MS *m*/*z* 234.0596 [M–H]^–^ (calculated for C_12_H_12_NO_2_S^–^, *m*/*z* 234.0589), 218.0644 [M–H_2_O + H]^+^ (calculated for C_12_H_12_NOS^+^, *m*/*z* 218.0640).

panunoid D (**4**). Colorless crystal. $\left[ \alpha \right]_{{\text{D}}}^{20}$ − 255.7 (*c* 0.11, MeOH); ^1^H and ^13^C NMR spectroscopic data see Table 1. Crystal data for **4**: Empirical formula C_11_H_18_O_5_; formula weight = 230.25; Temperature/K = 193.00; monoclinic space group C2; unit cell dimensions *a* = 38.2965(9) Å, *b* = 6.6639(2) Å, *c* = 9.2619(2) Å, *α* = *γ* = 90°, *β* = 98.7670(10)°; *V* = 2336.06(10) Å^3^; *Z* = 8; *ρ*_calc_ = 1.309 g/cm^3^; *μ* = 0.863 mm^−1^; *F*(000) = 992.0; crystal size 0.15 × 0.14 × 0.12 mm^3^; radiation CuK*α* (*λ* = 1.54178); 2*θ* range for data collection 9.662 to 136.784°; index ranges − 46 ≤ *h* ≤ 46, − 8 ≤ *k* ≤ 7, − 11 ≤ *l* ≤ 11; reflections collected 38,710; independent reflections 4263 [R_int_ = 0.0365, R_sigma_ = 0.0196]; data/restraints/parameters 4263/1/303; goodness-of-fit on F^2^ = 1.042; final R indexes [I ≥ 2*σ* (*I*)]: R_1_ = 0.0283, *w*R_2_ = 0.0723; final R indexes [all data]: R_1_ = 0.0290, *w*R_2_ = 0.0730; largest diff. peak/hole 0.17/-0.20 e Å^−3^; Flack parameter 0.03(4). Crystallographic data for compound **4** have been deposited at the Cambridge Structural Database with the number of CCDC-2417279.

7-desoxyphenepoxydol (**7**). White crystal. ^1^H and ^13^C NMR spectroscopic data see Table 2. HR-ESI–MS *m*/*z* 197.1174 [M + H] ^+^ (calculated for C_11_H_17_O_3_^+^, *m*/*z* 197.1178). Crystal data for **7**: Empirical formula C_11_H_16_O_3_; formula weight = 196.24; Temperature/K = 100.00(11); tetragonal space group P4_3_; unit cell dimensions *a* = 14.6405(2) Å, *b* = 14.6405(2) Å, *c* = 4.81930(10) Å, *α* = *β* = *γ* = 90°; *V* = 1032.99(4) Å^3^; *Z* = 4; *ρ*_calc_ = 1.262 g/cm^3^; *μ* = 0.740 mm^−1^; *F*(000) = 424.0; crystal size 0.35 × 0.06 × 0.05 mm^3^; radiation CuK*α* (*λ* = 1.54184); 2*θ* range for data collection 6.036 to 147.882°; index ranges − 16 ≤ *h* ≤ 18, − 14 ≤ *k* ≤ 18, − 5 ≤ *l* ≤ 5; reflections collected 7787; independent reflections 2024 [R_int_ = 0.0352, R_sigma_ = 0.0270]; data/restraints/parameters 2024/1/131; goodness-of-fit on F^2^ = 1.100; final R indexes [I ≥ 2*σ* (*I*)]: R_1_ = 0.0384, *w*R_2_ = 0.1022; final R indexes [all data]: R_1_ = 0.0393, *w*R_2_ = 0.1026; largest diff. peak/hole 0.14/-0.20 e Å^−3^; Flack parameter 0.04(13). Crystallographic data for compound **7** have been deposited at the Cambridge Structural Database with the number of CCDC-2496230.

(1*S*,3*S*,4*R*,6*S*,7*S*)-3,12-Dihydroxycadin-10(15)-ene (**12**). Colorless crystal. ^1^H NMR (400 MHz, CD_3_OD): *δ* 4.60 (d, *J* = 1.8 Hz, 1H, H-15), 4.50 (brs, 1H, H-15), 3.82 (q, *J* = 2.9 Hz, 1H, H-3), 1.18 (s, 3H, H-13), 1.12 (s, 3H, H-14), 0.95 (d, *J* = 6.9 Hz, 3H, H-11). ^13^C NMR (101 MHz, CD_3_OD): *δ* 154.5 (C-10), 104.0 (C-15), 74.7 (C-12), 71.1 (C-3), 54.0 (C-7), 48.1 (C-1), 40.1 (C-6), 38.1 (C-2), 38.0 (C-9), 37.6 (C-4), 35.8 (C-5), 32.4 (C-8), 31.5 (C-14), 24.8 (C-13), 19.4 (C-11). Crystal data for **12**: Empirical formula C_120_H_208_O_16_; formula weight = 1906.85; Temperature/K = 149.99(10); triclinic space group P1; unit cell dimensions *a* = 13.31910(10) Å, *b* = 14.7838(2) Å, *c* = 17.4435(2) Å, *α* = 114.1670(10)°, *β* = 92.5210(10)°, *γ* = 105.8640(10)°; *V* = 2966.42(6) Å^3^; *Z* = 1; ρ_calc_ = 1.067 g/cm^3^; *μ* = 0.532 mm^− 1^; *F*(000) = 1056.0; crystal size 0.2 × 0.13 × 0.12 mm^3^; radiation CuK*α* (*λ* = 1.54184); 2*θ* range for data collection 5.642 to 147.832°; index ranges − 16 ≤ *h* ≤ 16, − 17 ≤ *k* ≤ 18, − 21 ≤ *l* ≤ 21; reflections collected 74,242; independent reflections 22,440 [R_int_ = 0.0331, R_sigma_ = 0.0318]; data/restraints/parameters 22,440/3/1263; goodness-of-fit on F^2^ = 1.042; final R indexes [I ≥ 2*σ* (I)]: R_1_ = 0.0340, *w*R_2_ = 0.0857; final R indexes [all data]: R_1_ = 0.0382, *w*R_2_ = 0.0875; largest diff. peak/hole 0.31/-0.21 e Å^−3^; Flack parameter 0.04(4). Crystallographic data for compound **12** have been deposited at the Cambridge Structural Database with the number of CCDC-2498699.

### Cytotoxicity assay

Two cancer cell lines, human lung cancer A-549 cells and human liver cancer HepG2 cells were used in the cytotoxic assay. Cells were digested with 0.25% trypsin and collected, followed by centrifugation at 1000 rpm for 4 min. After discarding the supernatant, 4 mL of fresh medium was added to resuspend the cell pellet. A 20 *μ*L aliquot of cell suspension was mixed with 20 *μ*L trypan blue solution for cell counting, with cell viability required to exceed 90%. The cell density was adjusted to 2000 cells/100 *μ*L. A 96-well plate was loaded with 100 *μ*L cell suspension per well and incubated overnight at 37°C with 5% CO_2_ to allow cell adhesion. Test compounds at specified concentrations were added according to experimental requirements, with blank wells (cell-free wells) established as background controls. After 24, 48, and 72 h of treatment respectively, the culture medium was removed and replaced with 100 *μ*L fresh medium containing 0.5% FBS per well. Subsequently, 10 *μ*L CCK-8 solution was added to each well, followed by 1-h incubation at 37 °C (avoiding bubble formation that may interfere with OD readings). The optical density was measured at 450 nm using a microplate reader, and calculate the cell viability using Eq. (1). The IC_50_ values of compounds were calculated by Graphpad Prism 10.1.2 (GraphPad Software Inc., San Diego, CA, USA). All determinations were carried out in triplicate.

1 $$\begin{array}{*{20}c} {Cell \, viability \, \left( {\text{\%}} \right) = \left( {{\text{OD}}_{2} - {\text{OD}}_{0} } \right)/\left( {{\text{OD}}_{1} { } - {\text{ OD}}_{0} } \right) \times 100} \\ \end{array}$$

where OD_0_ is the noise group, OD_1_ is the blank control group, and OD_2_ is the test group.

### ECD calculation

The calculated structures were optimized with the Gaussian 09 package [35]. The B3LYP functional was used to all calculations in combination with the D3BJ dispersion correction. Time-dependent density functional theory (TD-DFT) were used to calculated the energy levels of the excited singlets. ECD spectrum were taken with multiwfn 3.8 software. The spectral data of different conformations were weighted to generate conformational average spectra.

## Conclusions

In summary, this study focused on the rice fermentation products of *P. rudis*. A series of secondary metabolites were systematically characterized by a combination of multiple separation and characterization methods. Among them, four new compounds were found and the absolute configurations of **7** and **12** were determined to be unreported. At the same time, a reasonable biosynthetic pathway for **1**–**7** was inferred. From the results, it can be seen that compared with other substrates, solid-state fermentation using rice as the matrix tends to produce metabolites with carbonyl groups reduced to hydroxyl groups. In addition, compounds with potential cytotoxicity were screened. The results showed that **1**, **3**–**6**, **8**, **9**, and **12** exhibited cytotoxicity on A-549 and HepG2 cells, and **8** and **12** exhibited dose-dependent inhibitory effects on the two cell lines. This study not only expands the chemical profile of *P. rudis* solid-state fermentation products, but also reveals that the integration of the fungal enzymatic machinery with nutrient-rich substrates could provide direction for discovering metabolites with both structural diversity and bioactivity.

## Supplementary Information

Additional file 1.

## Data Availability

Data will be made available on request.
