# Supplementary material for: Panunoids A – D, four new prenylhydroquinone derivatives isolated from the fungus Panus rudis
Source: Nat Prod Bioprospect. 2026 Jan 9;16(1):9. doi: 10.1007/s13659-025-00562-3 (PMC12783394; doi:10.1007/s13659-025-00562-3)
Supplement: Supplementary file 1 — Additional file 1. [file 13659_2025_562_MOESM1_ESM.docx]

**Contents**

**Figure S1.** ^1^H NMR spectrum (400MHz, CD_3_OD) of **1**

**Figure S2.** ^13^C NMR spectrum (101MHz, CD_3_OD) of **1**

**Figure S3.** ^1^H-^1^H COSY spectrum of **1**

**Figure S4.** HSQC spectrum of **1**

**Figure S5.** HMBC spectrum of **1**

**Figure S6.** NOESY spectrum of **1**

**Figure S7.** ROESY spectrum of **1**

**Figure S8.** HR-ESI-MS spectrum of **1**

**Figure S9.** ^1^H NMR spectrum (400MHz, CD_3_OD) of **2**

**Figure S10.** ^13^C NMR spectrum (101MHz, CD_3_OD) of **2**

**Figure S11.** ^1^H-^1^H COSY spectrum of **2**

**Figure S12.** HSQC spectrum of **2**

**Figure S13.** HMBC spectrum of **2**

**Figure S14.** NOESY spectrum of **2**

**Figure S15.** HR-ESI-MS spectrum of **2**

**Figure S16.** ^1^H NMR spectrum (400MHz, CD_3_OD) of **3**

**Figure S17.** ^13^C NMR spectrum (101MHz, CD_3_OD) of **3**

**Figure S18.** ^1^H-^1^H COSY spectrum of **3**

**Figure S19.** HSQC spectrum of **3**

**Figure S20.** HMBC spectrum of **3**

**Figure S21.** HR-ESI-MS spectrum of **3**

**Figure S22.** HR-ESI-MS spectrum of **3**

**Figure S23.** ^1^H NMR spectrum (400MHz, DMSO) of **4**

**Figure S24.** ^1^H NMR spectrum (600MHz, CD3OD) of **4**

**Figure S25.** ^13^C NMR spectrum (101MHz, DMSO) of **4**

**Figure S26.** ^1^H-^1^H COSY spectrum of **4**

**Figure S27.** HSQC spectrum of **4**

**Figure S28.** HMBC spectrum of **4**

**Figure S29.** ROESY spectrum of **4**

**Figure S30.** ^1^H NMR spectrum (400MHz, CD_3_OD) of **5**

**Figure S31.** ^13^C NMR spectrum (101MHz, CD_3_OD) of **5**

**Figure S32.** ^1^H-^1^H COSY spectrum of **5**

**Figure S33.** NOESY spectrum of **5**

**Figure S34.** HR-ESI-MS spectrum of **5**

**Figure S35.** ^1^H NMR spectrum (400MHz, CD_3_OD) of **6**

**Figure S36.** ^13^C NMR spectrum (101MHz, CD_3_OD) of **6**

**Figure S37.** ^1^H-^1^H COSY spectrum of **6**

**Figure S38.** HSQC spectrum of **6**

**Figure S39.** HMBC spectrum of **6**

**Figure S40.** NOESY spectrum of **6**

**Figure S41.** ROESY spectrum of **6**

**Figure S42.** HR-ESI-MS spectrum of **6**

**Figure S43.** HR-ESI-MS spectrum of **6**

**Figure S44.** ^1^H NMR spectrum (400MHz, CD_3_OD) of **7**

**Figure S45.** ^13^C NMR spectrum (101MHz, CD_3_OD) of **7**

**Figure S46.** ^1^H-^1^H COSY spectrum of **7**

**Figure S47.** HSQC spectrum of **7**

**Figure S48.** HMBC spectrum of **7**

**Figure S49.** NOESY spectrum of **7**

**Figure S50.** ROESY spectrum of **7**

**Figure S51.** HR-ESI-MS spectrum of **7**

**Figure S52.** ^1^H NMR spectrum (400MHz, CD_3_OD) of **8**

**Figure S53.** ^13^C NMR spectrum (101MHz, CD_3_OD) of **8**

**Figure S54.** ^1^H NMR spectrum (400MHz, CD_3_OD) of **9**

**Figure S55.** ^13^C NMR spectrum (101MHz, CD_3_OD) of **9**

**Figure S56.** ^1^H NMR spectrum (400MHz, CD_3_OD) of **10**

**Figure S57.** ^13^C NMR spectrum (101MHz, CD_3_OD) of **10**

**Figure S58.** ^1^H NMR spectrum (400MHz, CD_3_OD) of **11**

**Figure S59.** ^13^C NMR spectrum (101MHz, CD_3_OD) of **11**

**Figure S60.** ^1^H NMR spectrum (400MHz, CD_3_OD) of **12**

**Figure S61.** ^13^C NMR spectrum (101MHz, CD_3_OD) of **12**


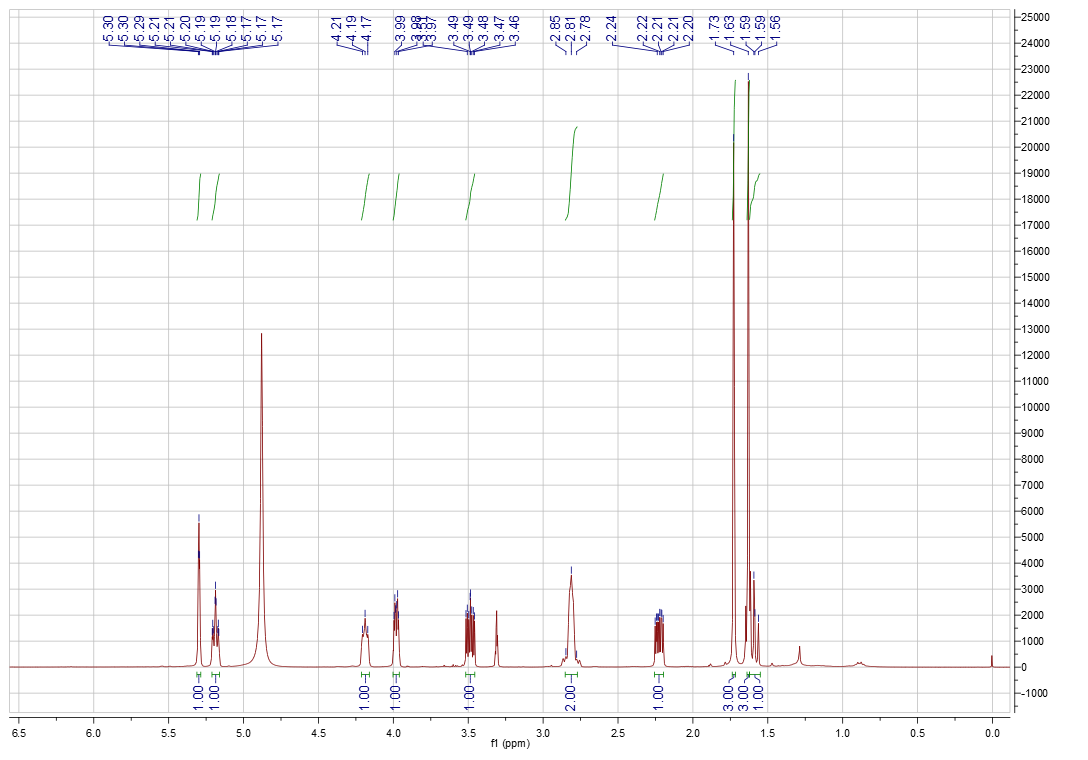


**Figure S1.** ^1^H NMR spectrum (400MHz, CD_3_OD) of **1**


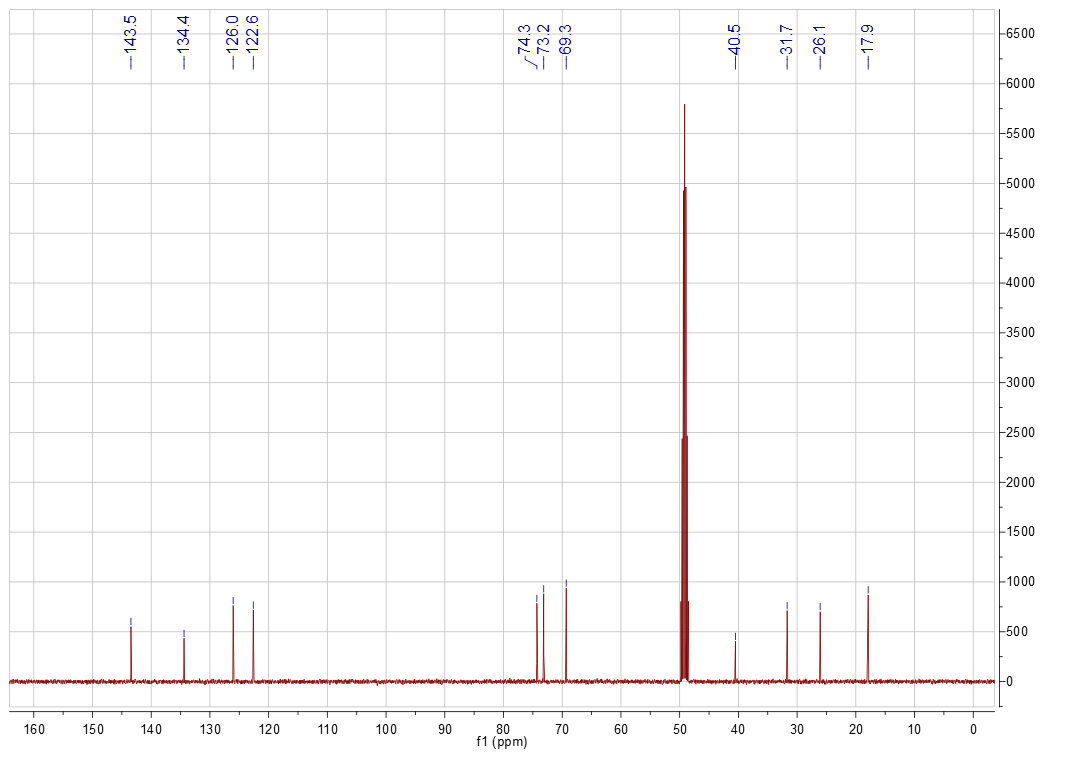


**Figure S2.** ^13^C NMR spectrum (101MHz, CD_3_OD) of **1**

**
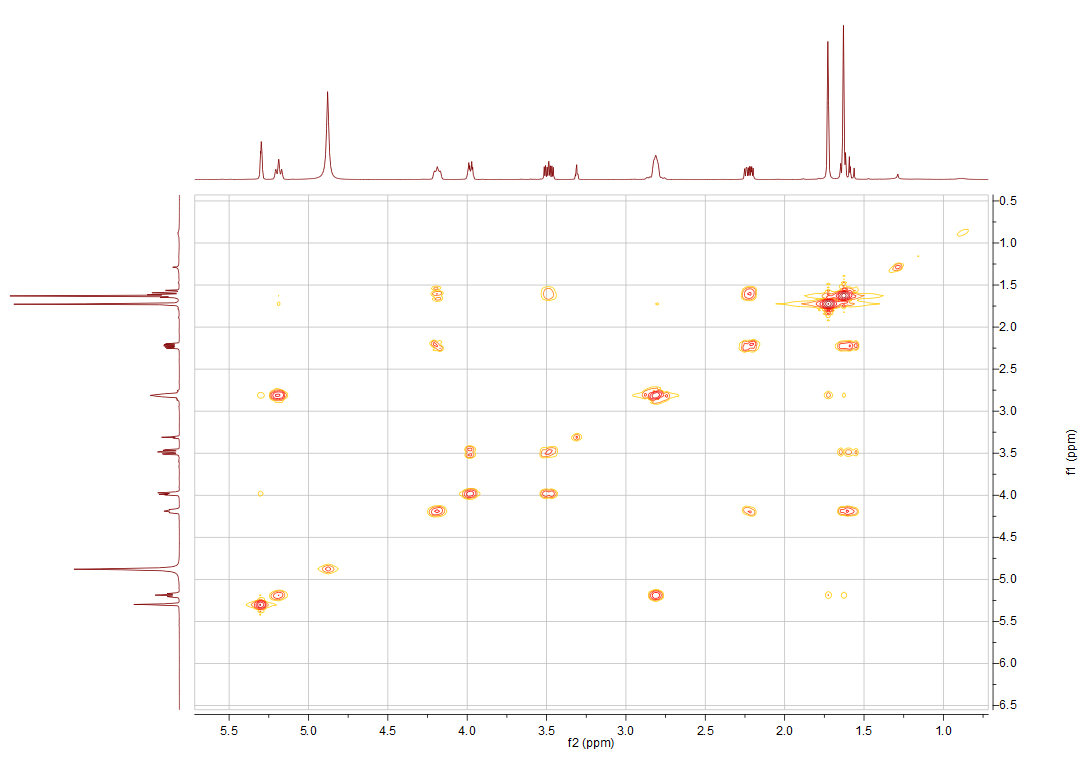
**

**Figure S3.** ^1^H-^1^H COSY spectrum of **1**


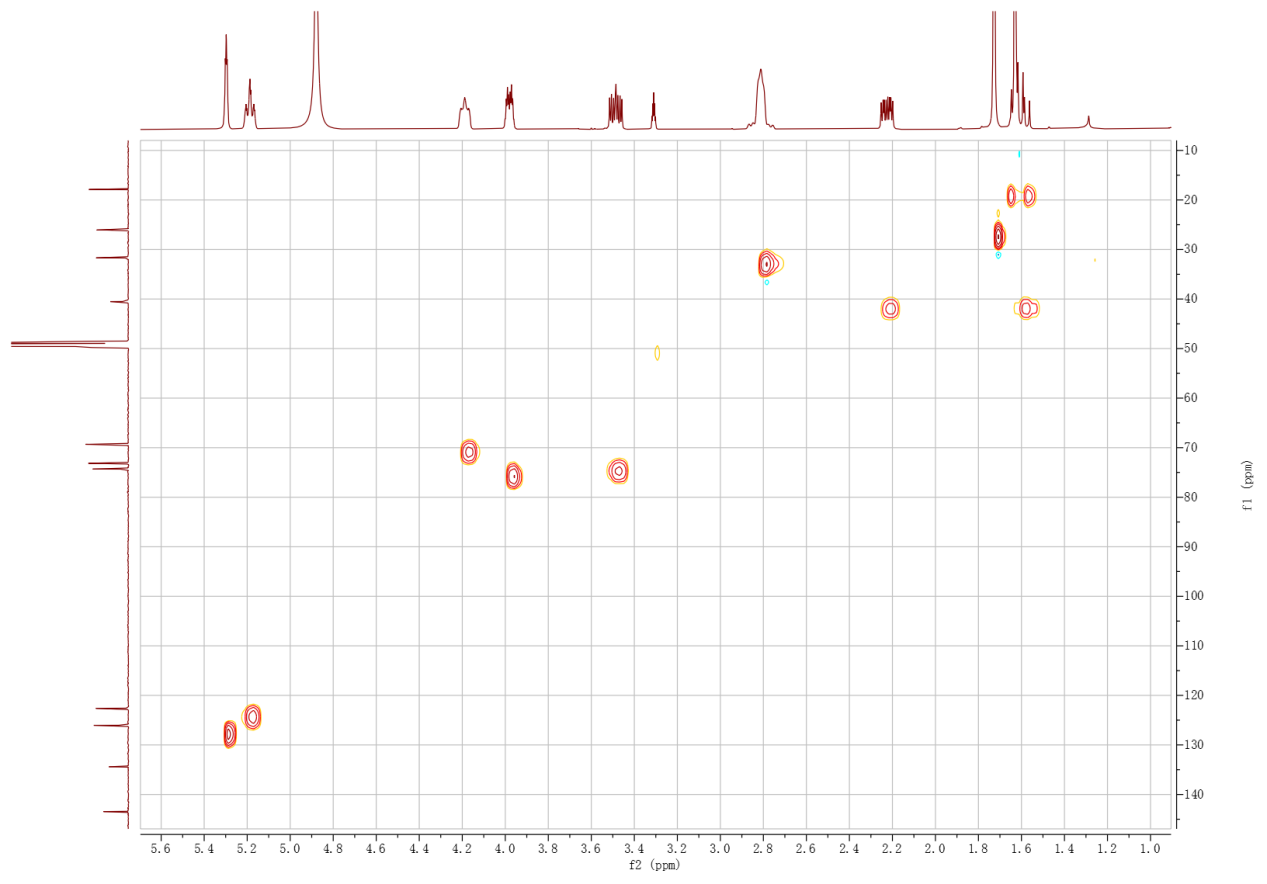


**Figure S4.** HSQC spectrum of **1**


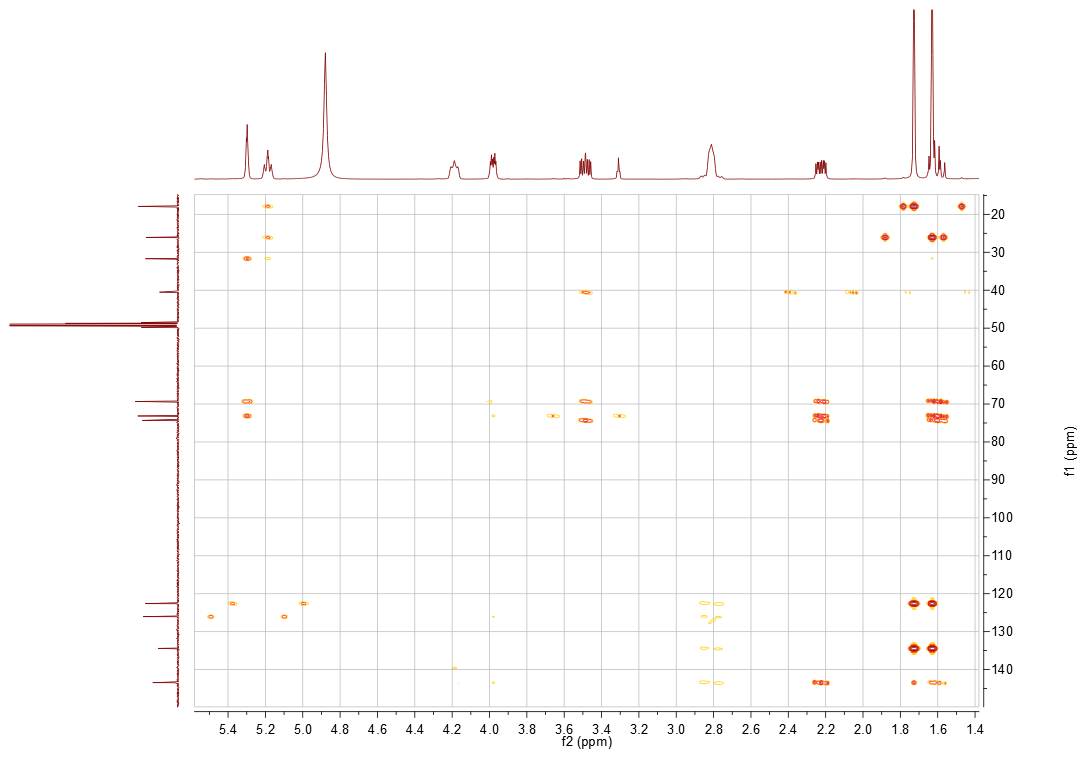


**Figure S5.** HMBC spectrum of **1**


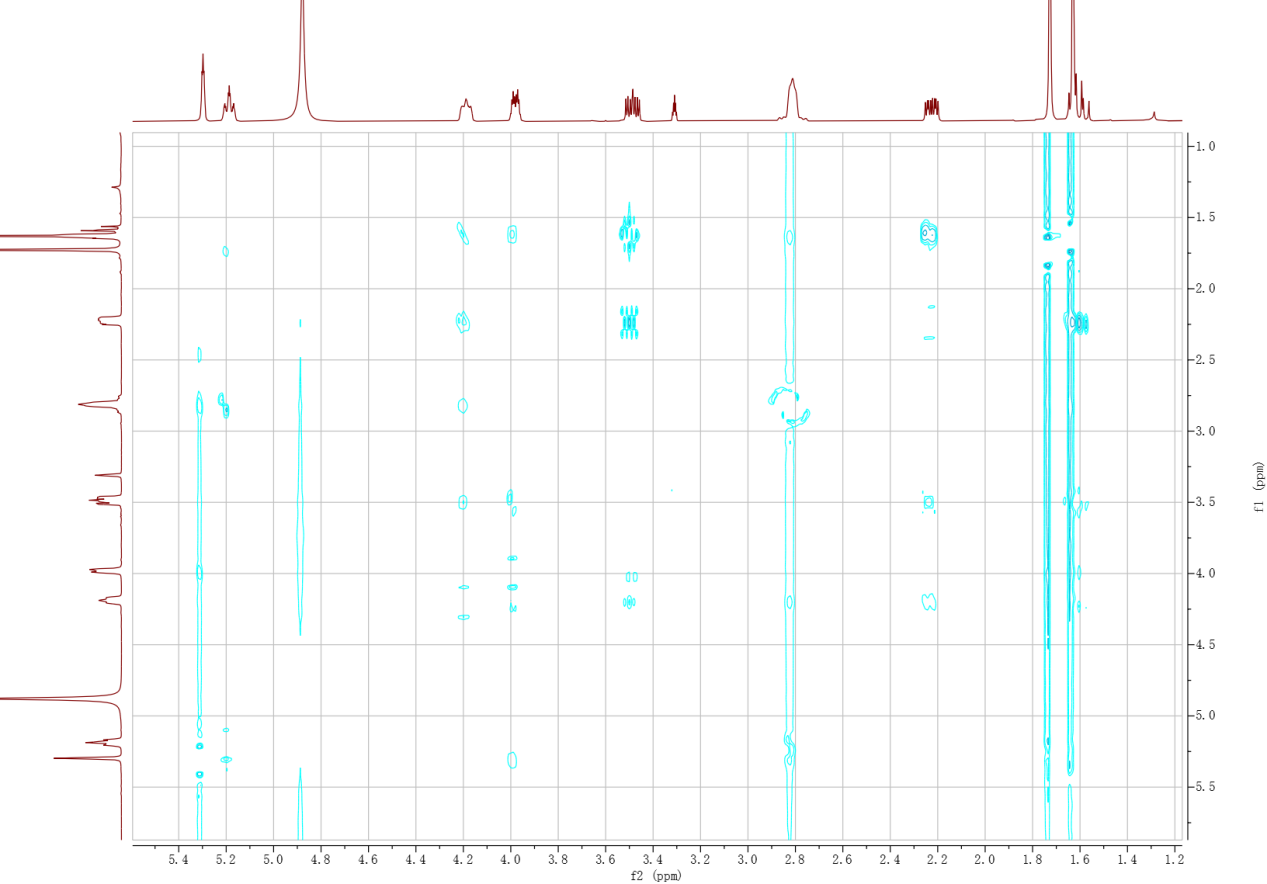


**Figure S6.** NOESY spectrum of **1**


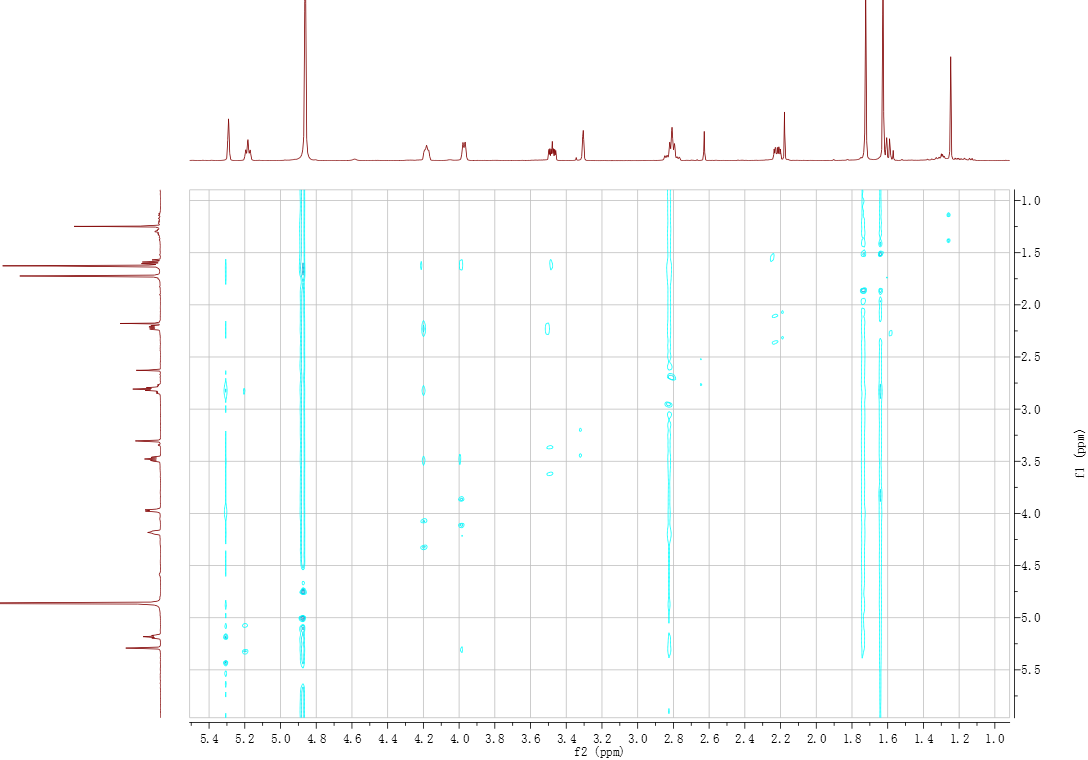


**Figure S7.** ROESY spectrum of **1**

**
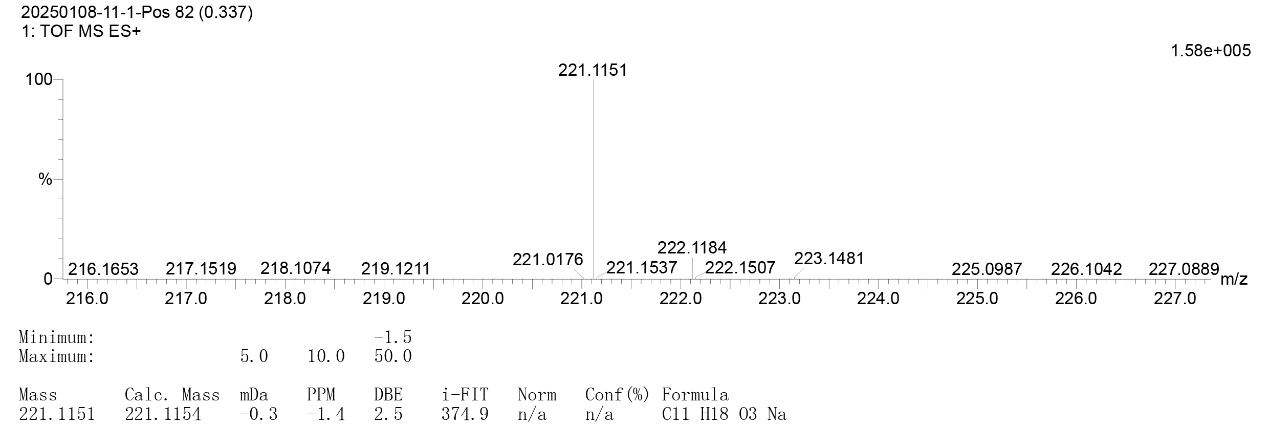
**

**Figure S8.** HR-ESI-MS spectrum of **1**


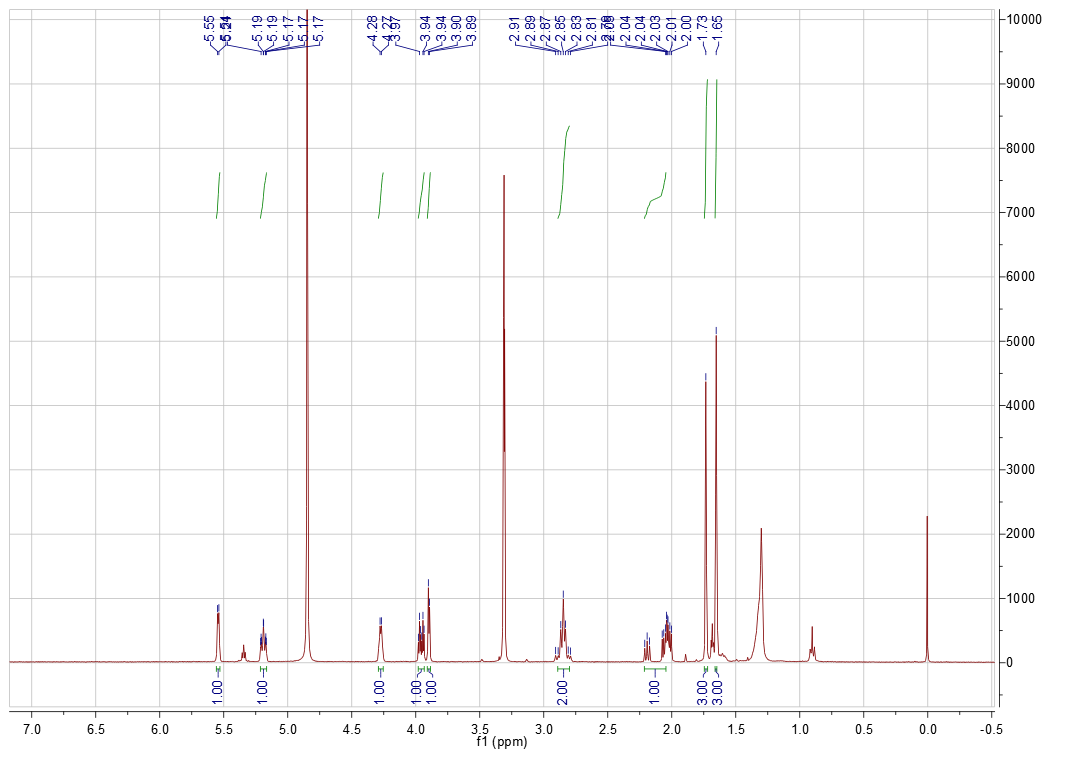


**Figure S9.** ^1^H NMR spectrum (400MHz, CD_3_OD) of **2**


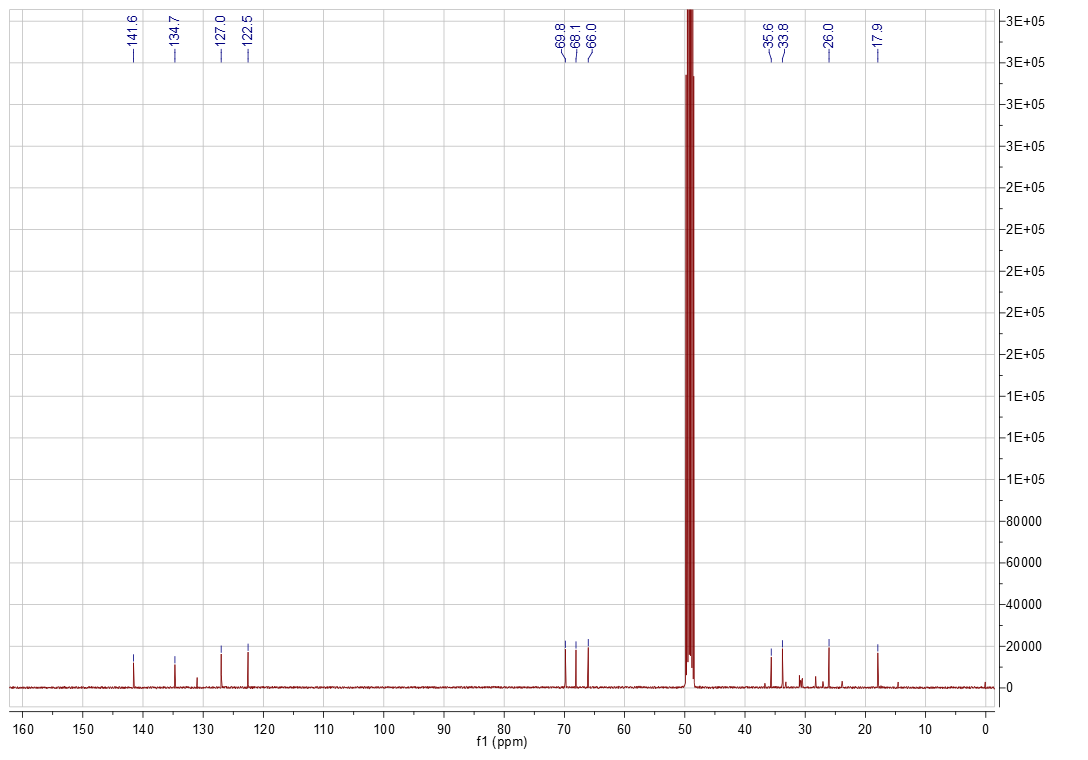


**Figure S10.** ^13^C NMR spectrum (101MHz, CD_3_OD) of **2**


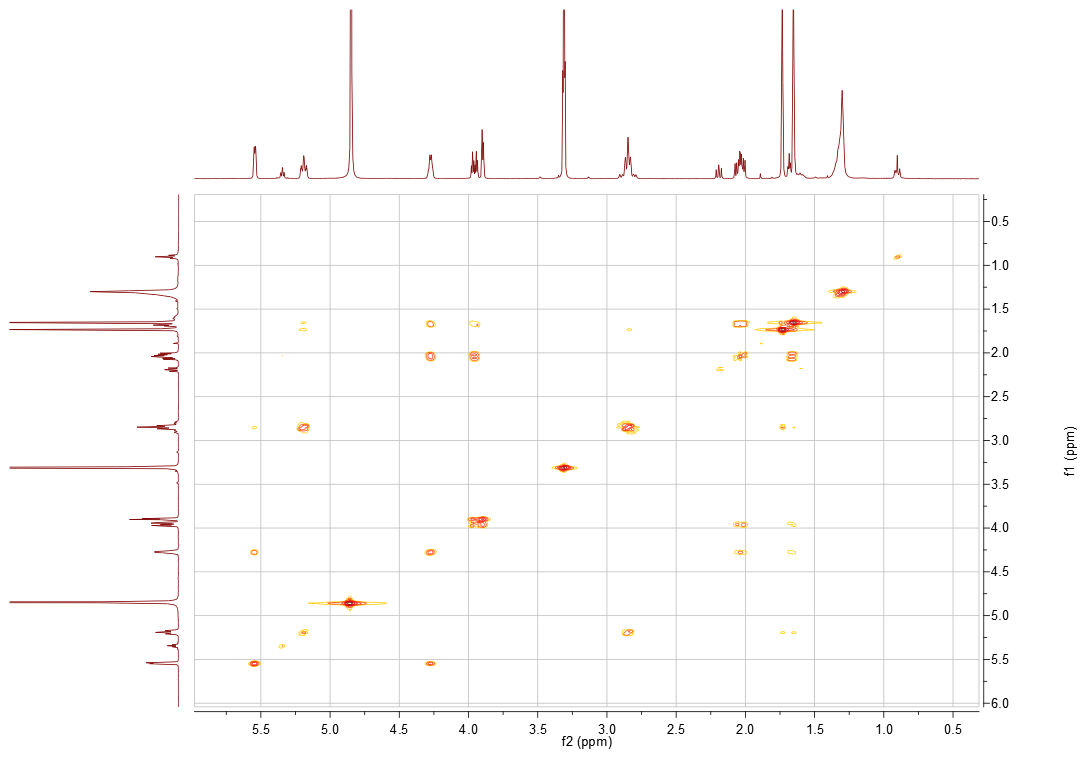


**Figure S11.** ^1^H-^1^H COSY spectrum of **2**


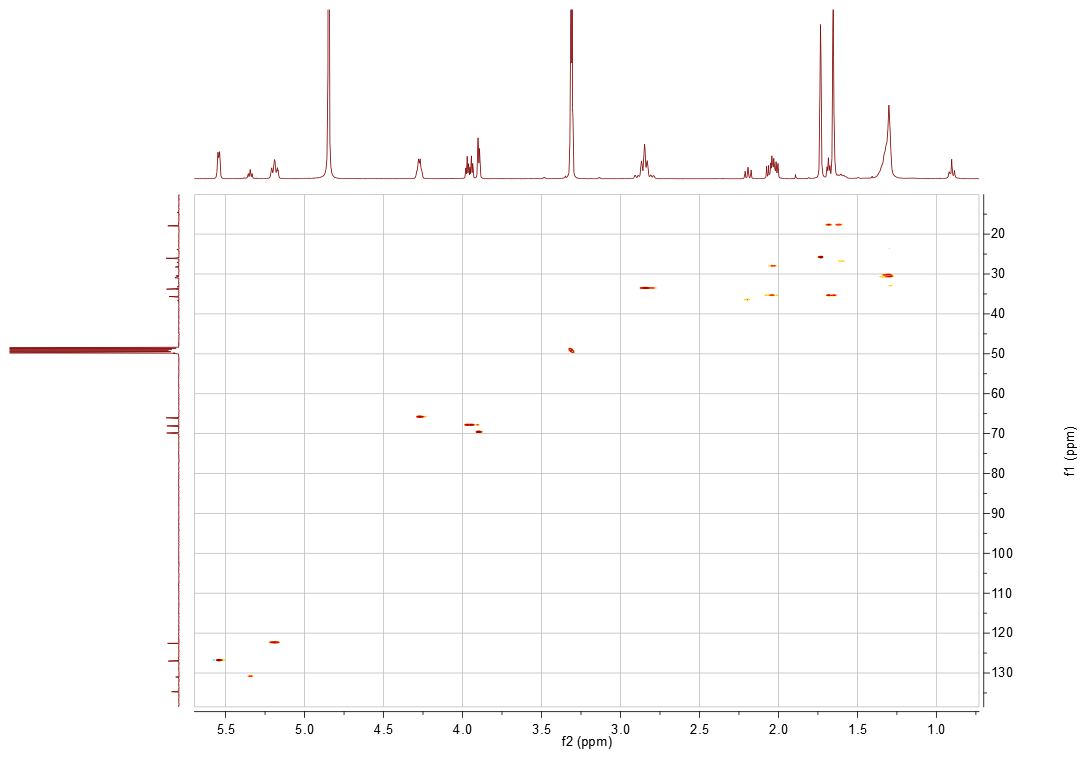


**Figure S12.** HSQC spectrum of **2**

**
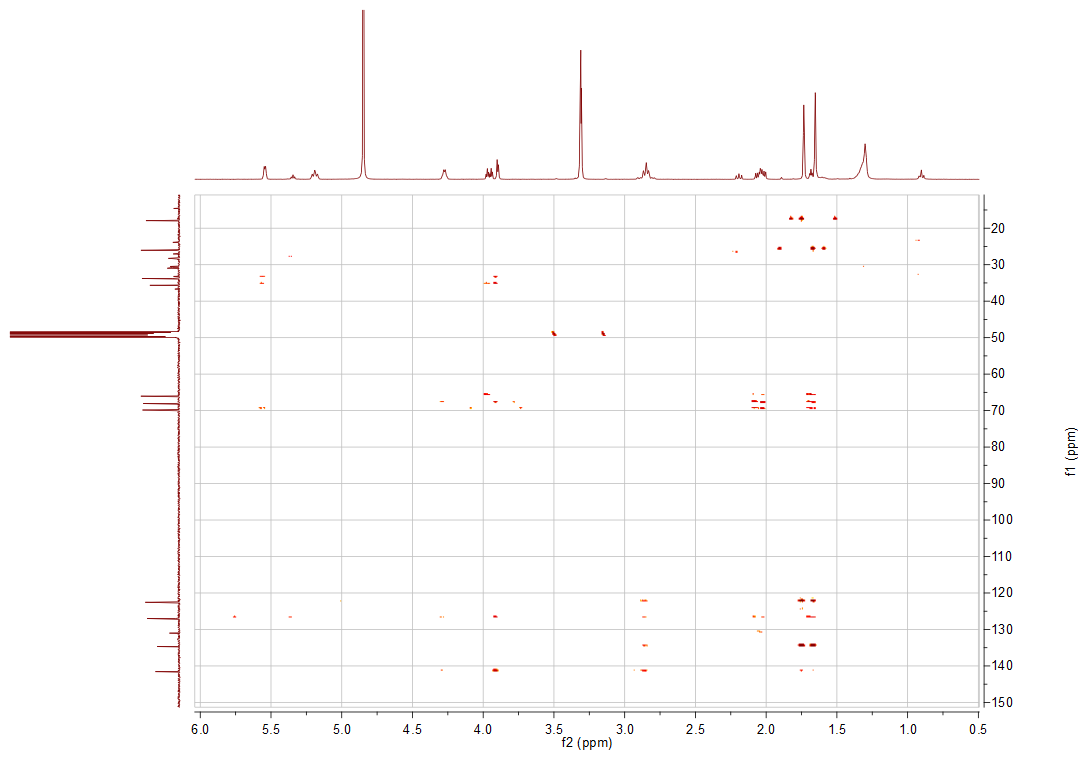
**

**Figure S13.** HMBC spectrum of **2**

**
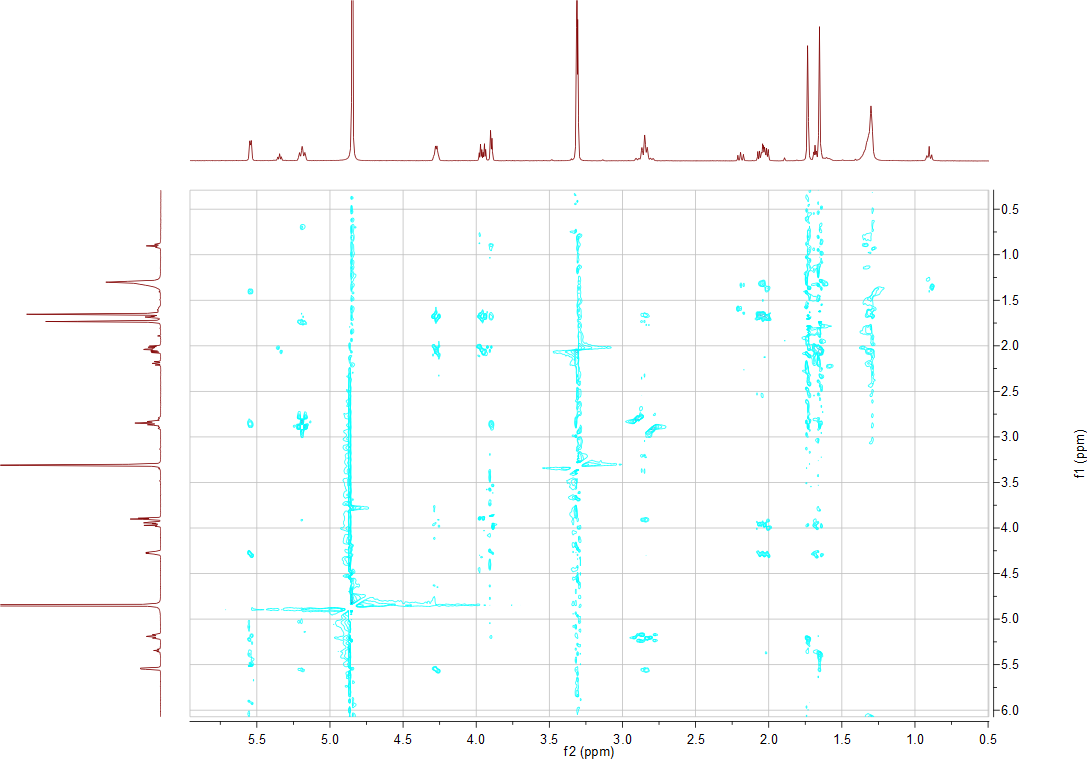
**

**Figure S14.** NOESY spectrum of **2**

**
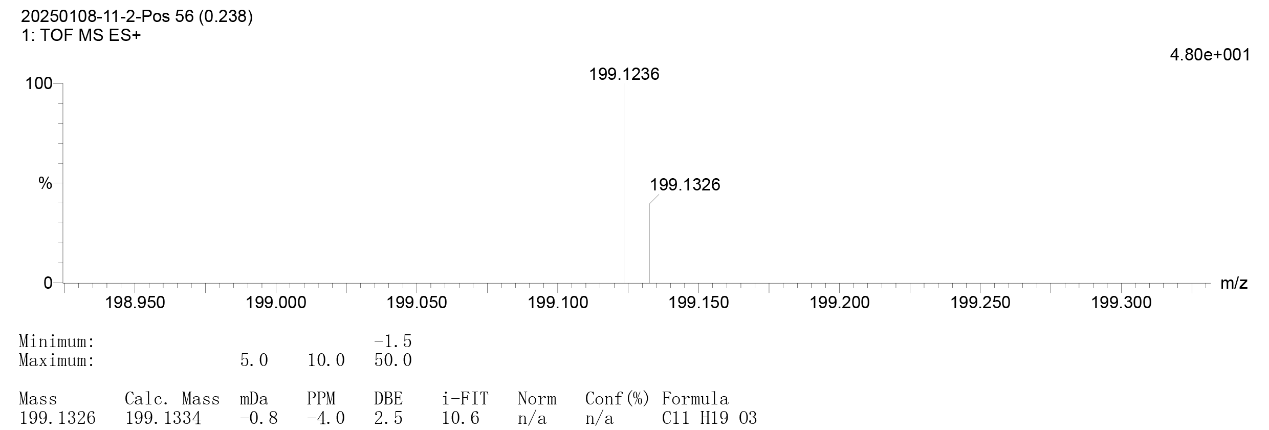
**

**Figure S15.** HR-ESI-MS spectrum of **2**


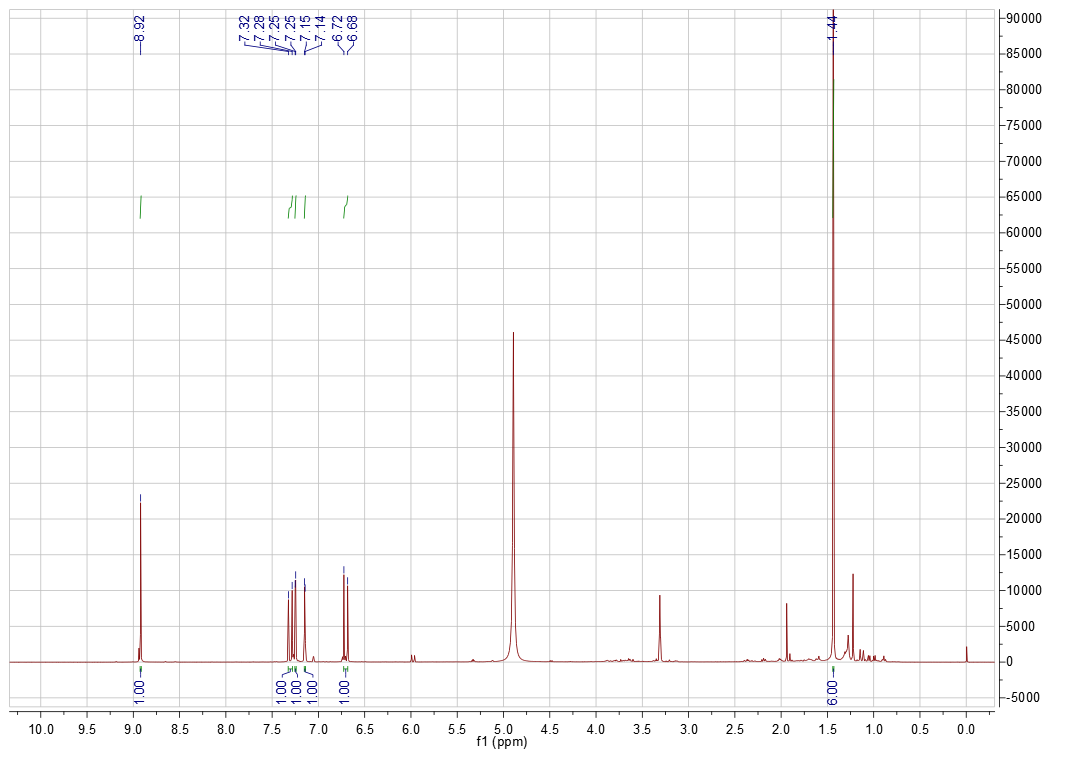


**Figure S16.** ^1^H NMR spectrum (400MHz, CD_3_OD) of **3**


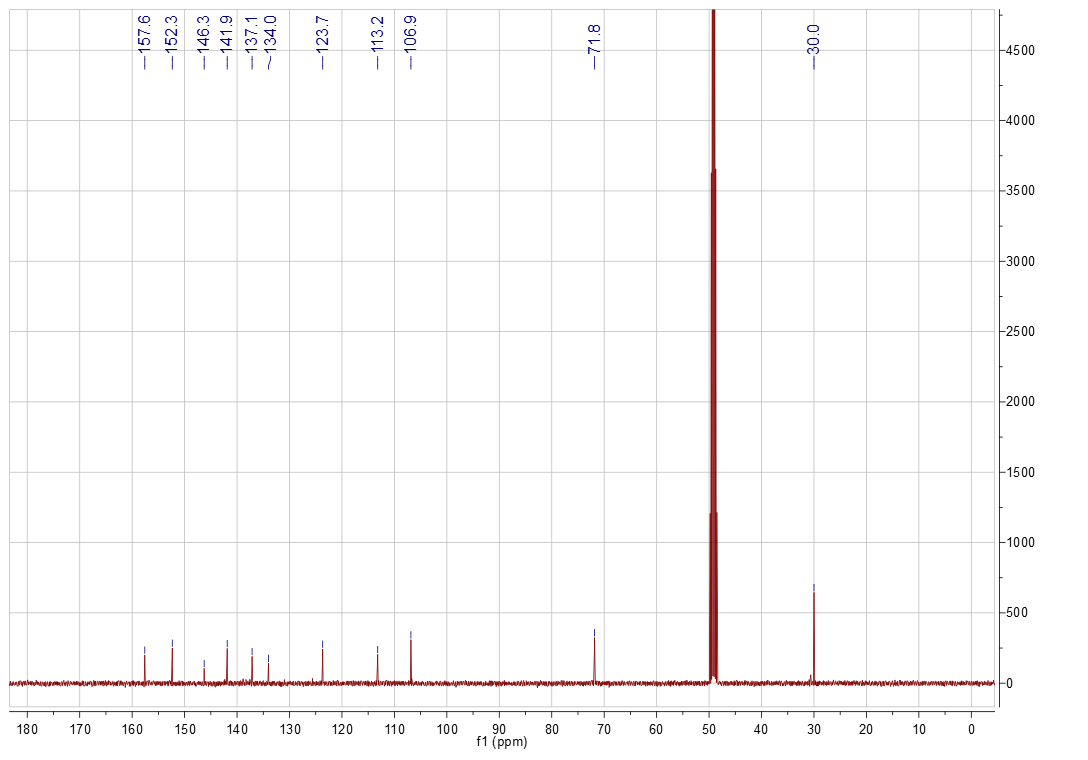


**Figure S17.** ^13^C NMR spectrum (101MHz, CD_3_OD) of **3**

**
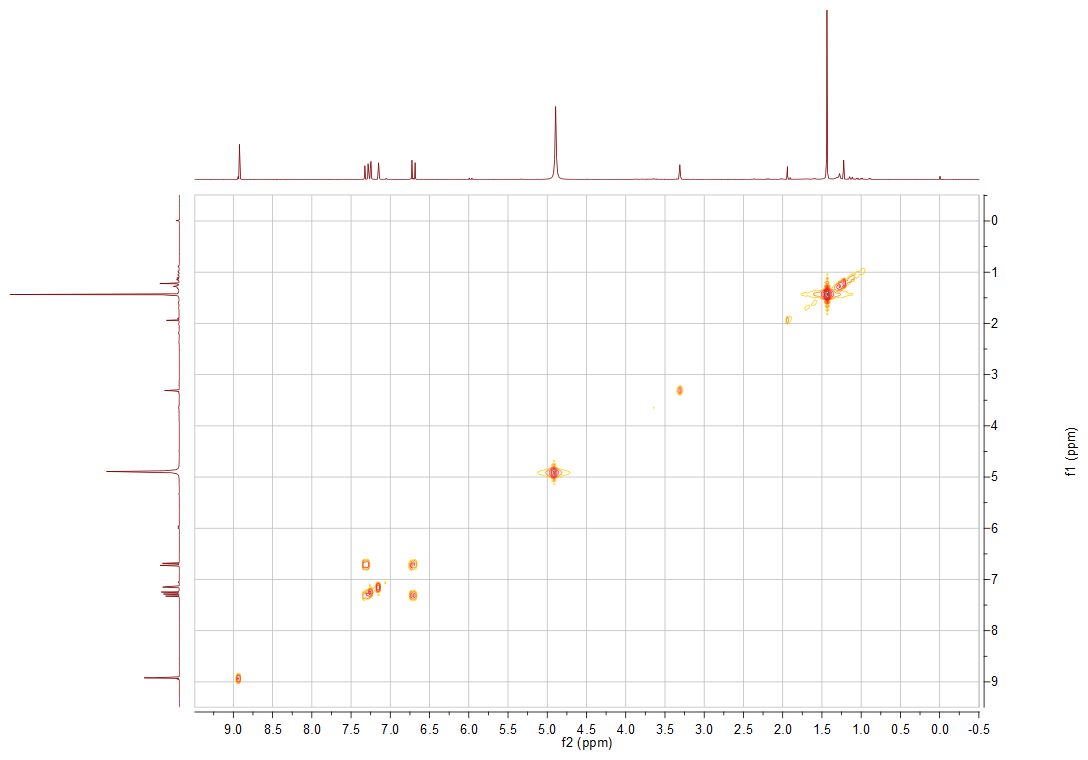
**

**Figure S18.** ^1^H-^1^H COSY spectrum of **3**


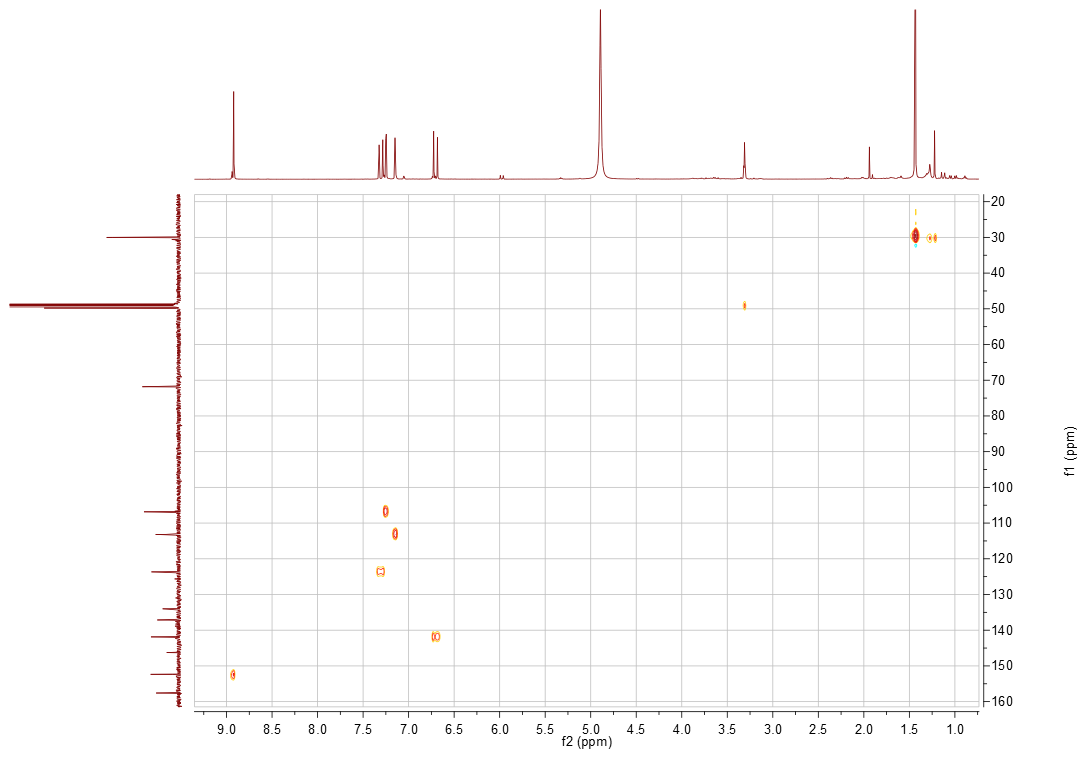


**Figure S19.** HSQC spectrum of **3**

**
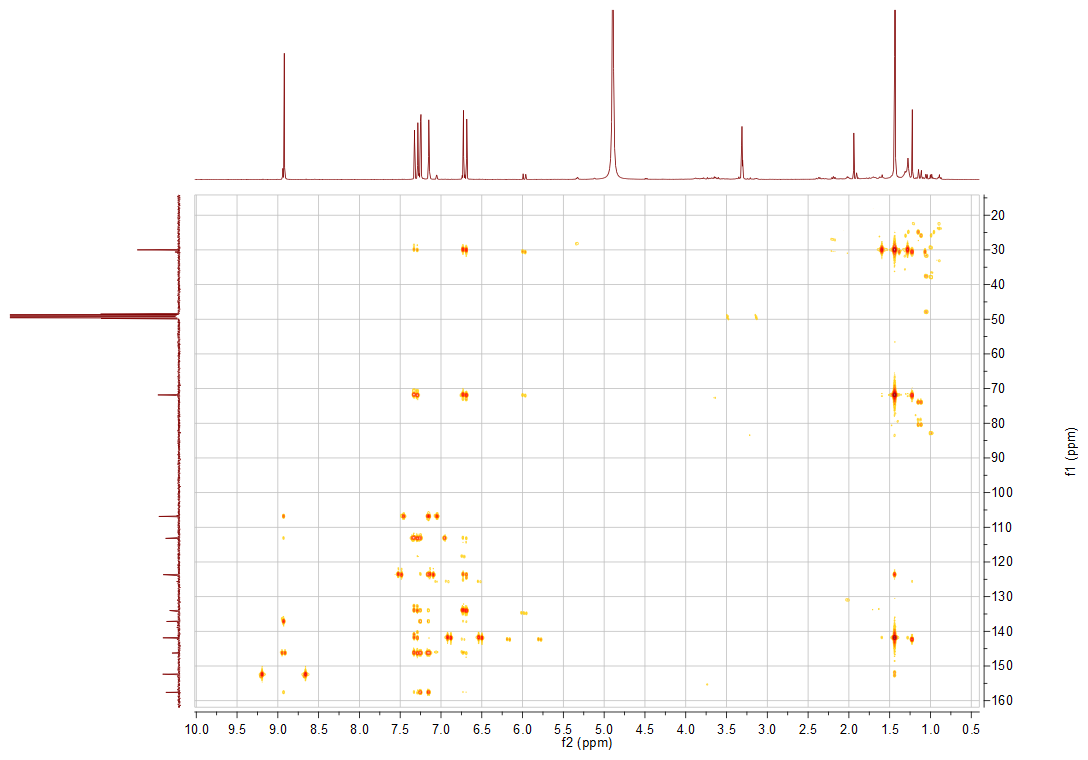
**

**Figure S20.** HMBC spectrum of **3**

**

**

**Figure S21.** HR-ESI-MS spectrum of **3**

**

**

**Figure S22.** HR-ESI-MS spectrum of **3**


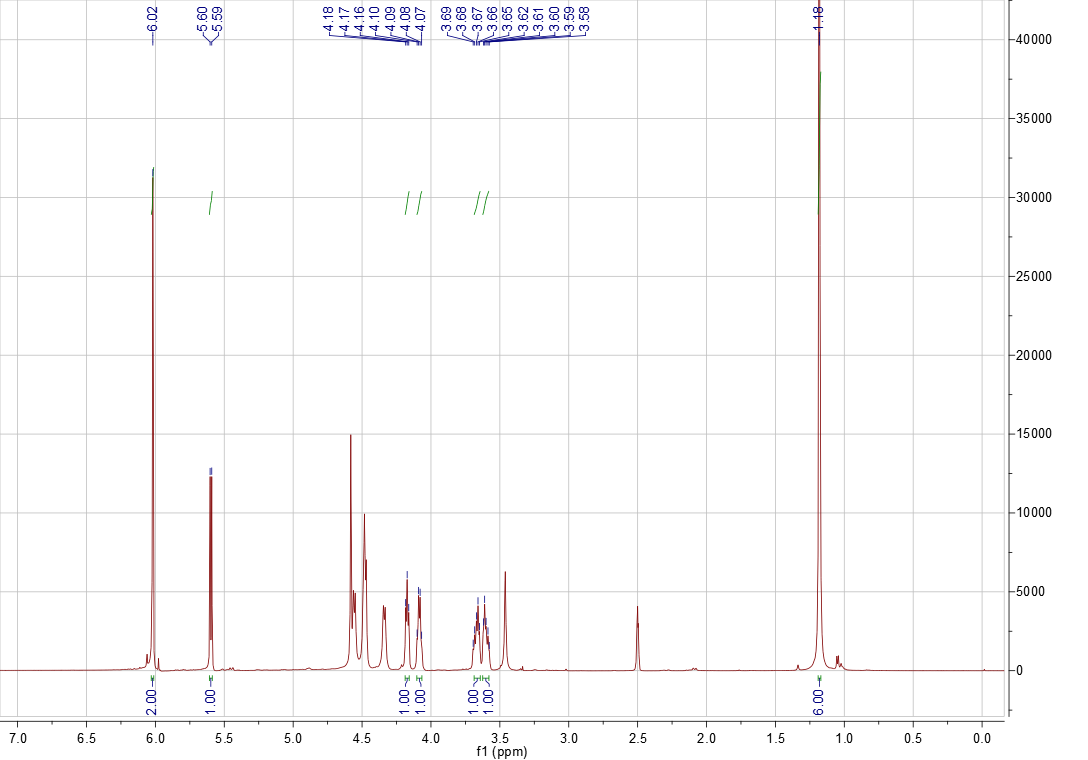


**Figure S23.** ^1^H NMR spectrum (400MHz, DMSO) of **4**


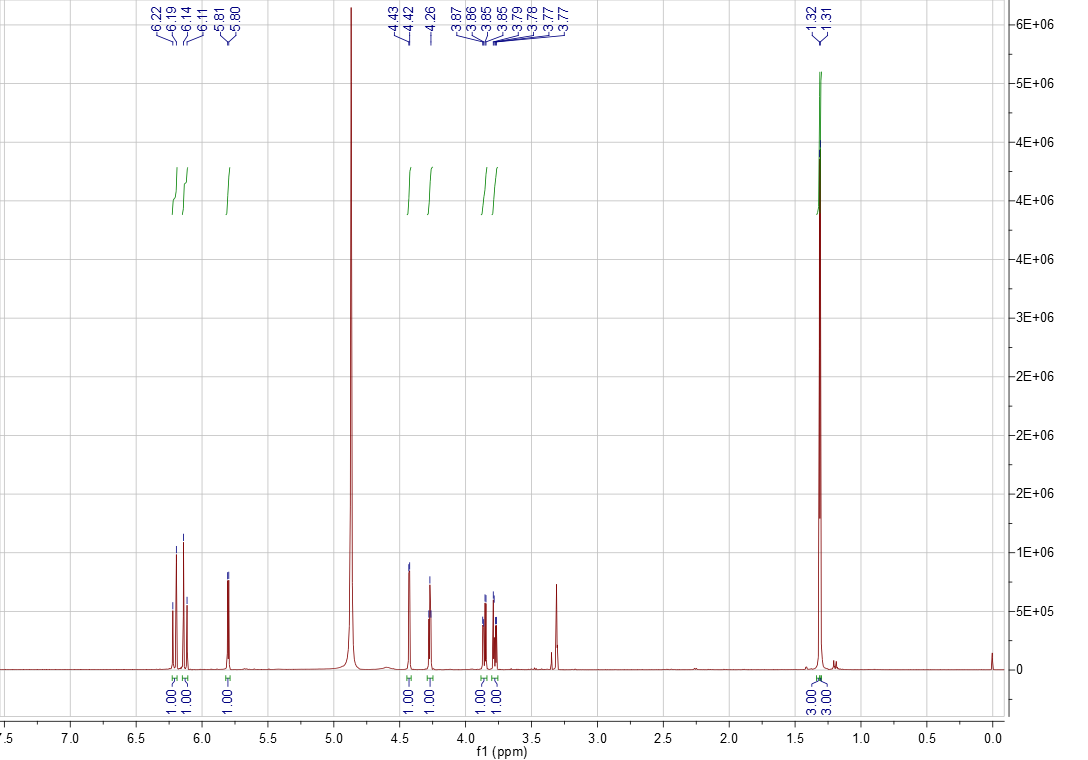


**Figure S24.** ^1^H NMR spectrum (600MHz, CD_3_OD) of **4**


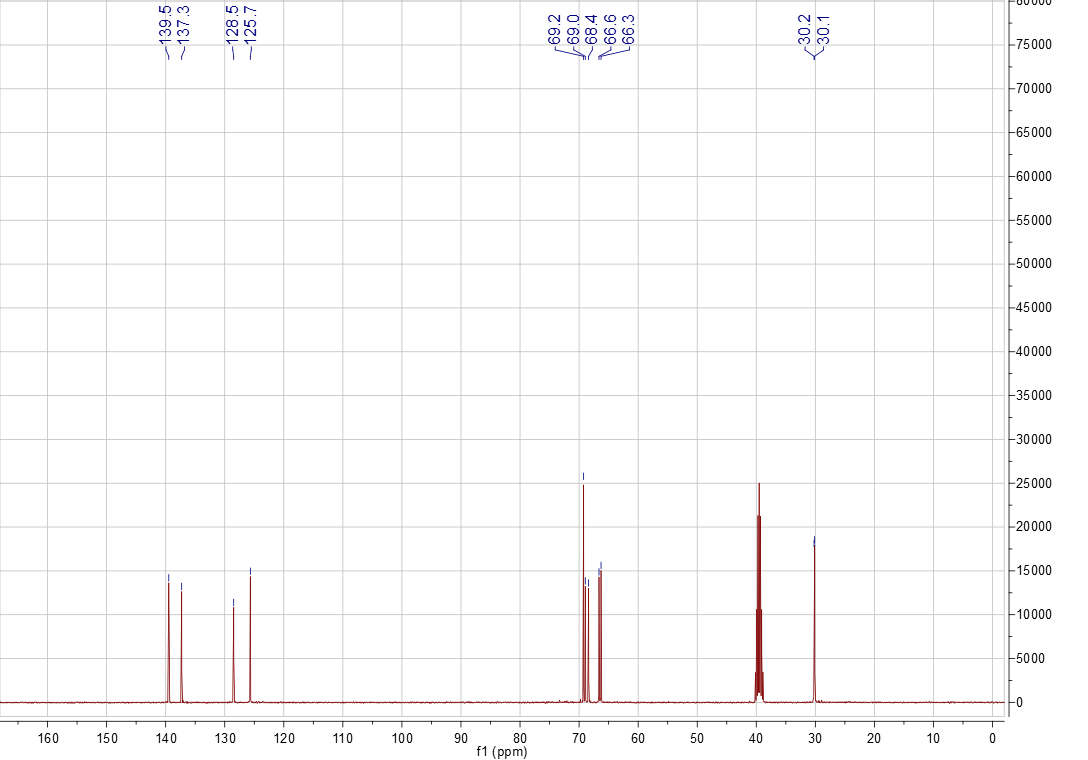


**Figure S25.** ^13^C NMR spectrum (101MHz, DMSO) of **4**


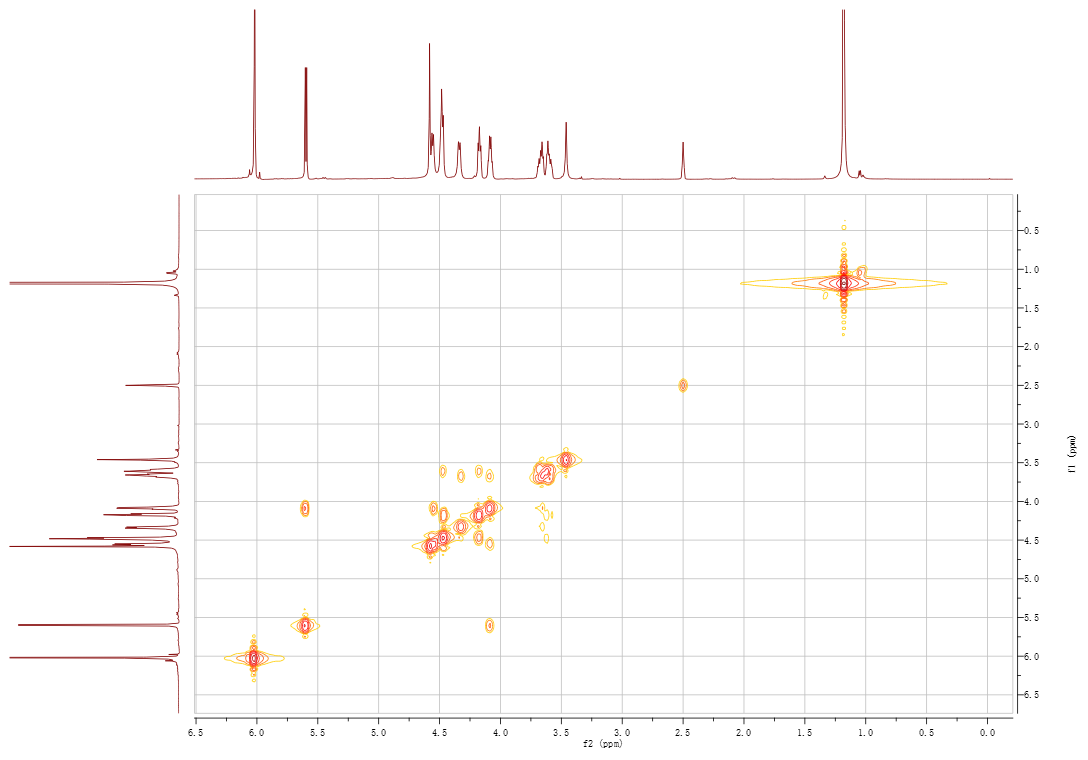


**Figure S26.** ^1^H-^1^H COSY spectrum of **4**


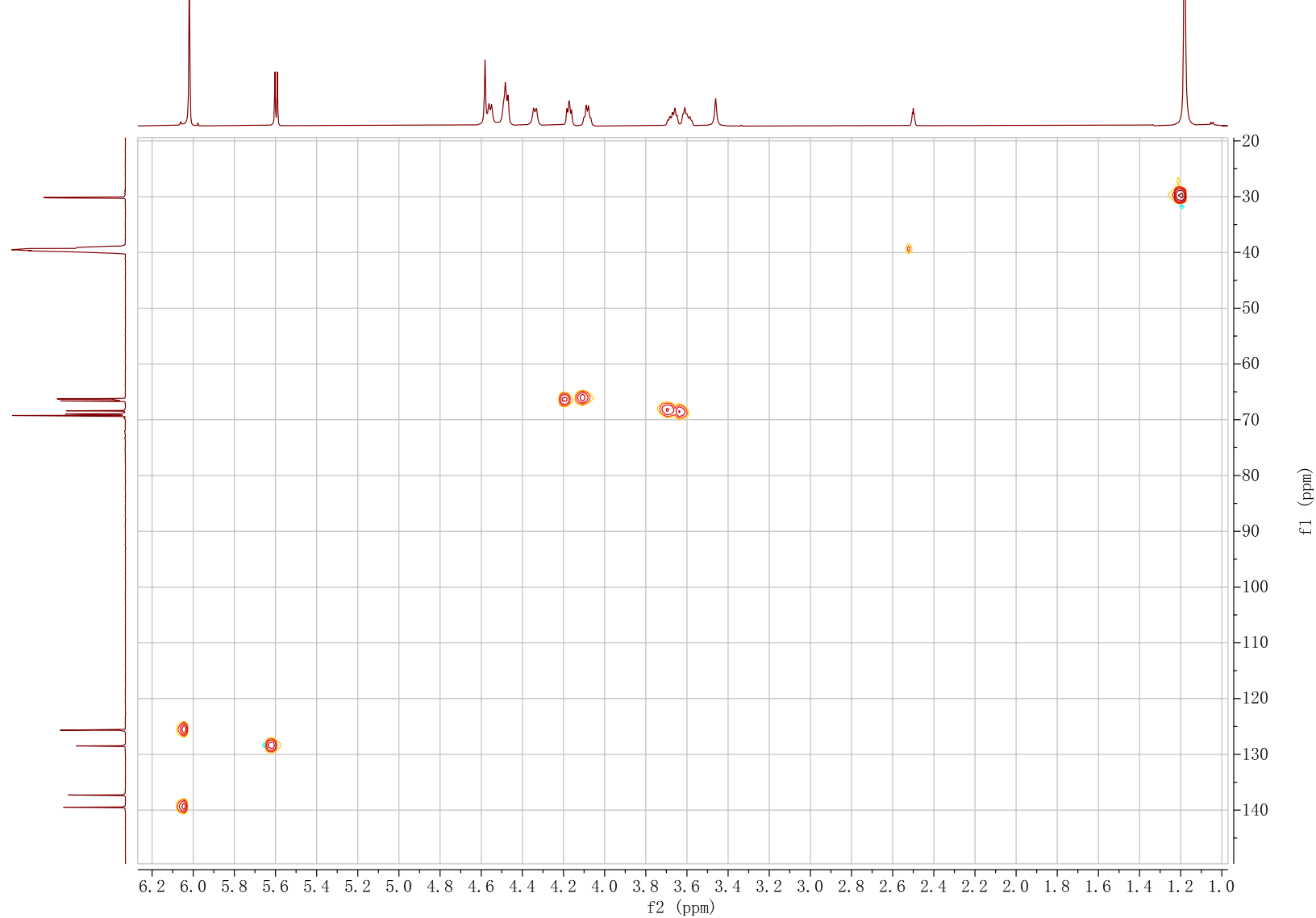


**Figure S27.** HSQC spectrum of **4**


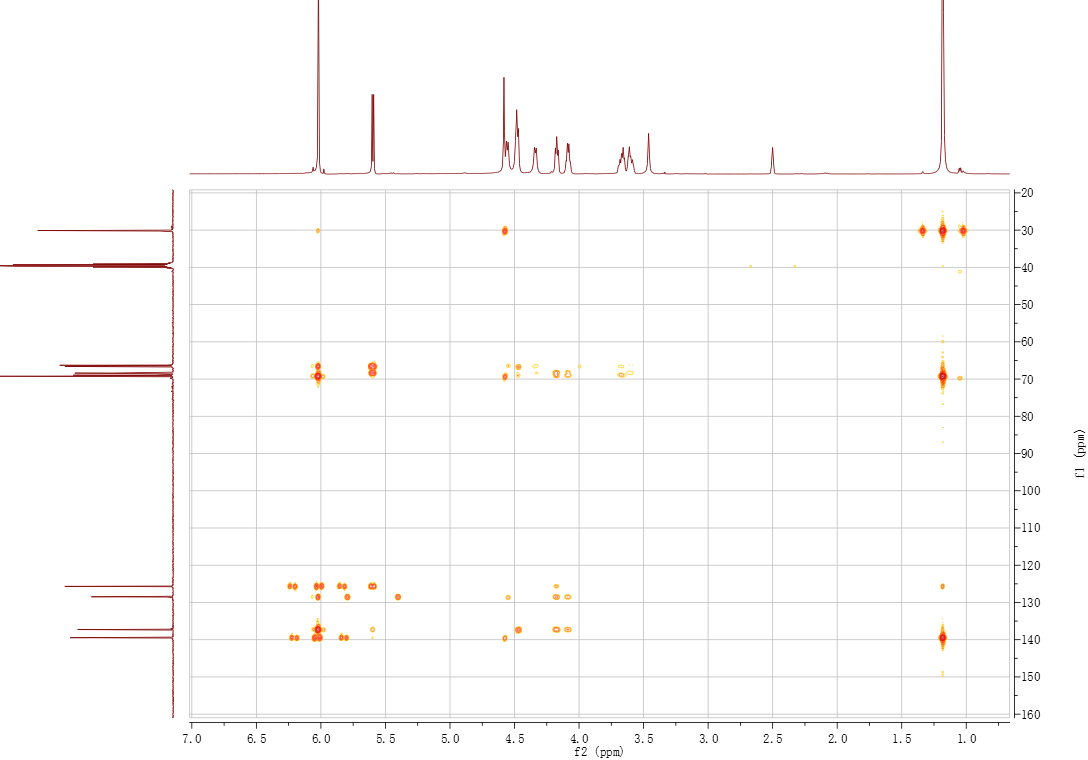


**Figure S28.** HMBC spectrum of **4**


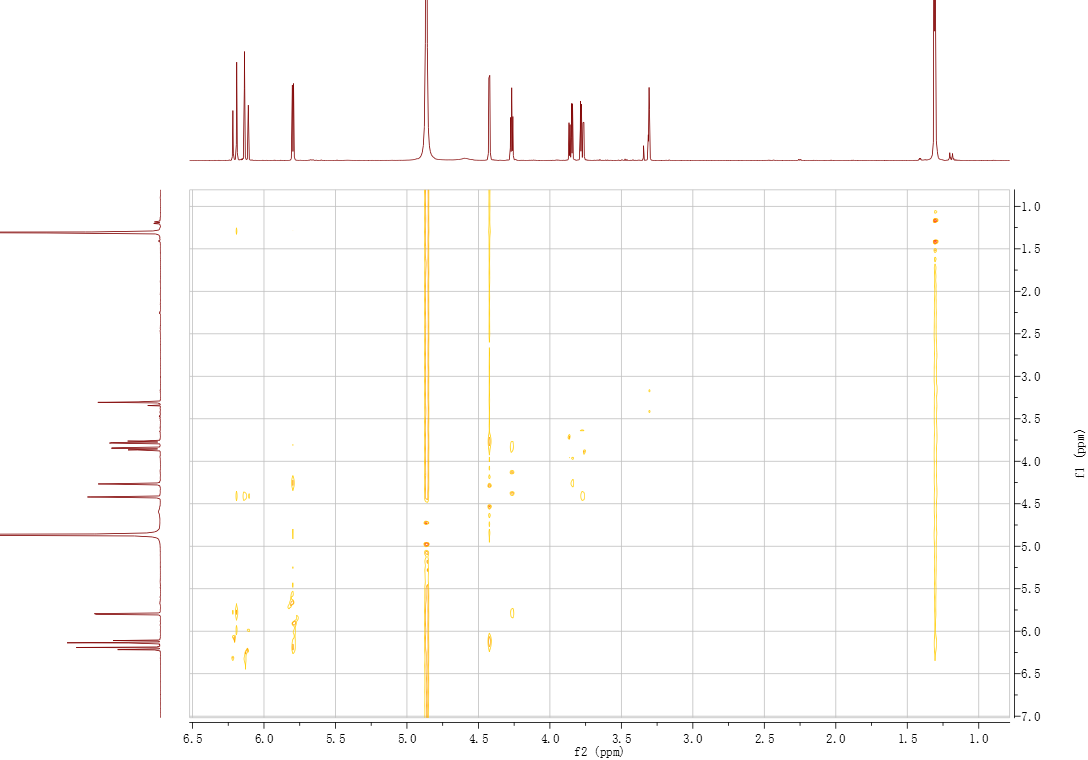


**Figure S29.** ROESY spectrum of **4**


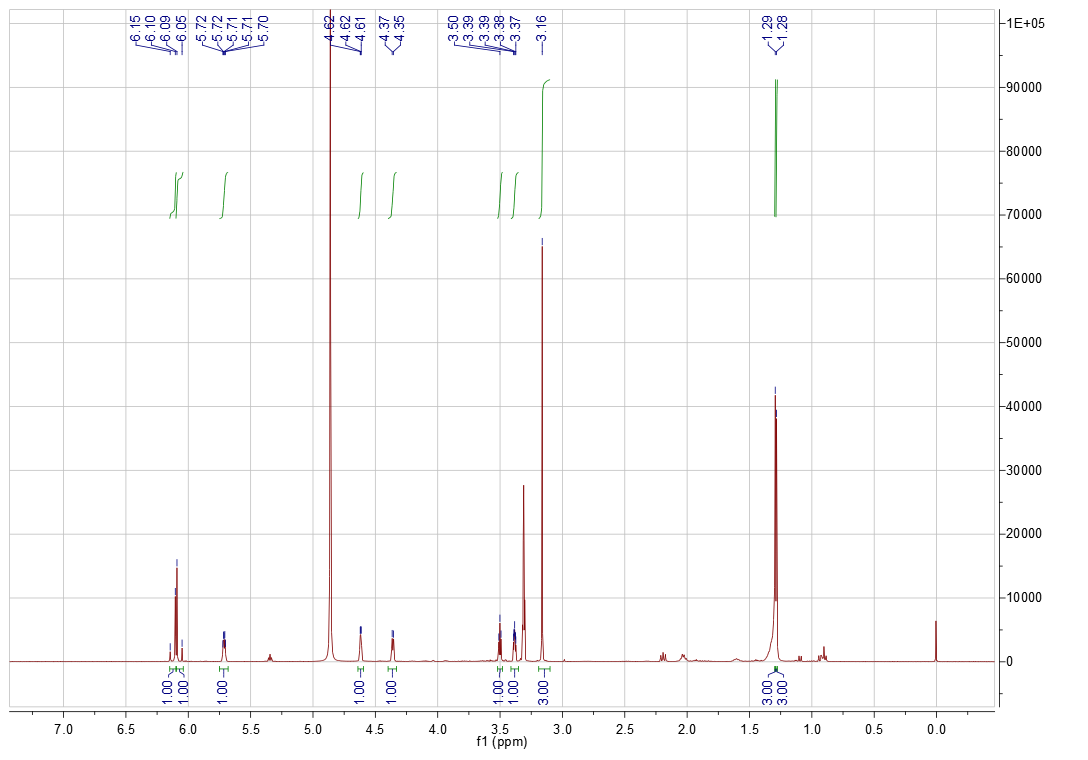


**Figure S30.** ^1^H NMR spectrum (400MHz, CD_3_OD) of **5**


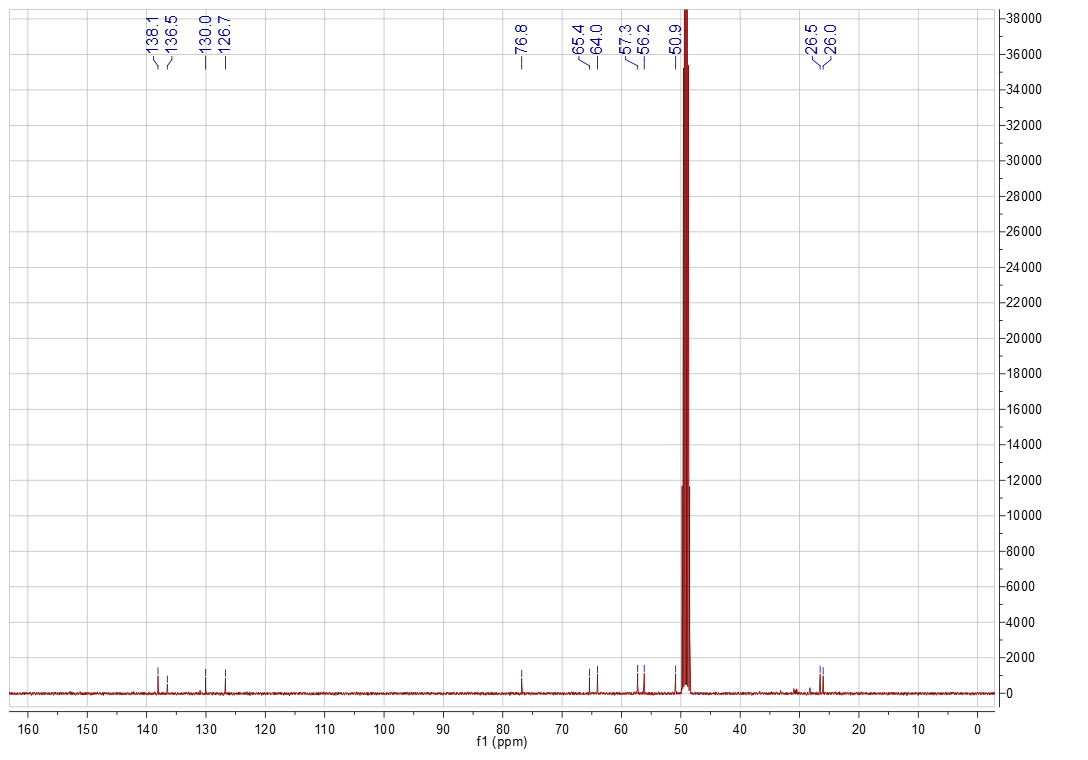


**Figure S31.** ^13^C NMR spectrum (101MHz, CD_3_OD) of **5**


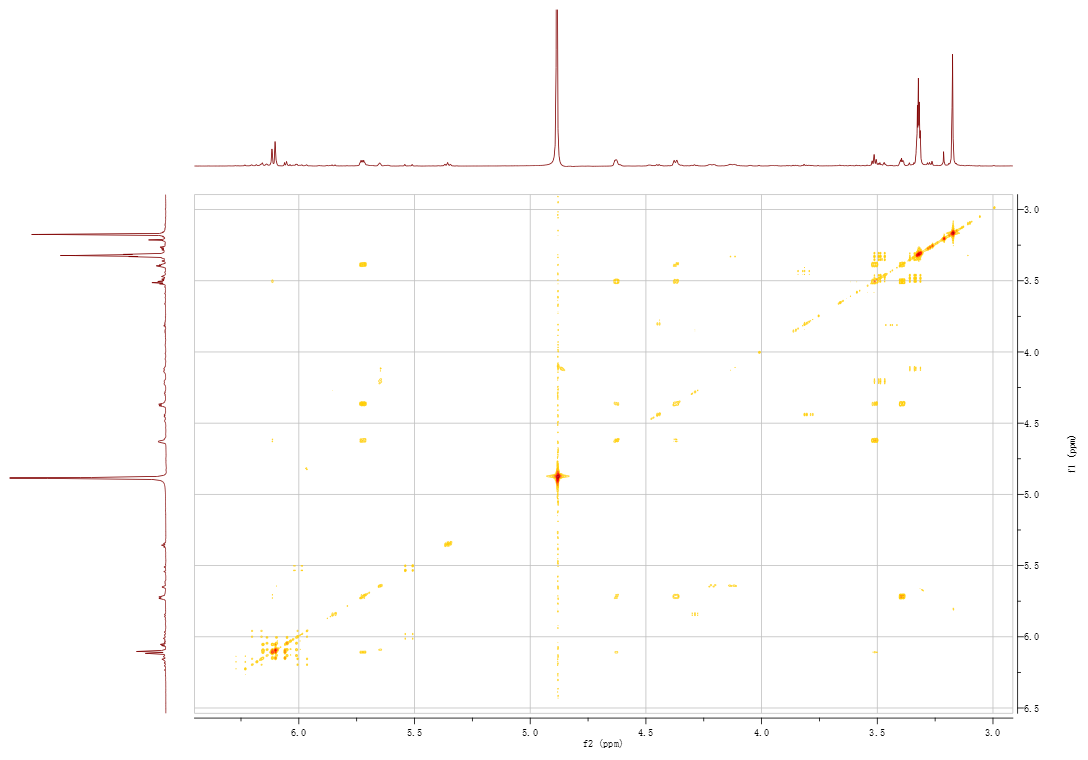


**Figure S32.** ^1^H-^1^H COSY spectrum of **5**


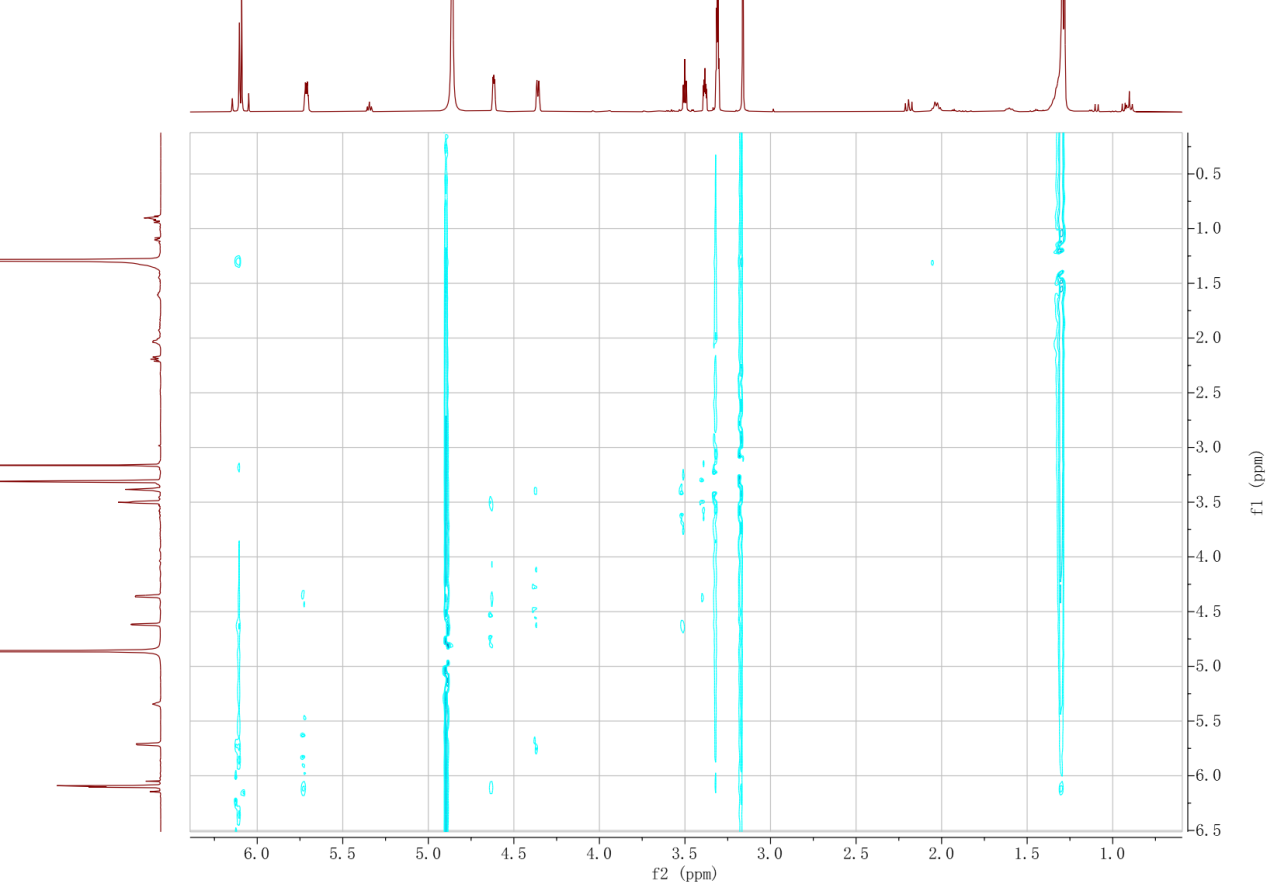


**Figure S33.** NOESY spectrum of **5**

**
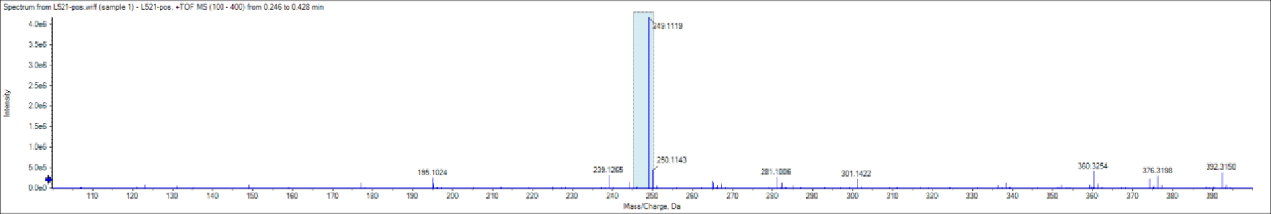
**

**Figure S34.** HR-ESI-MS spectrum of **5**


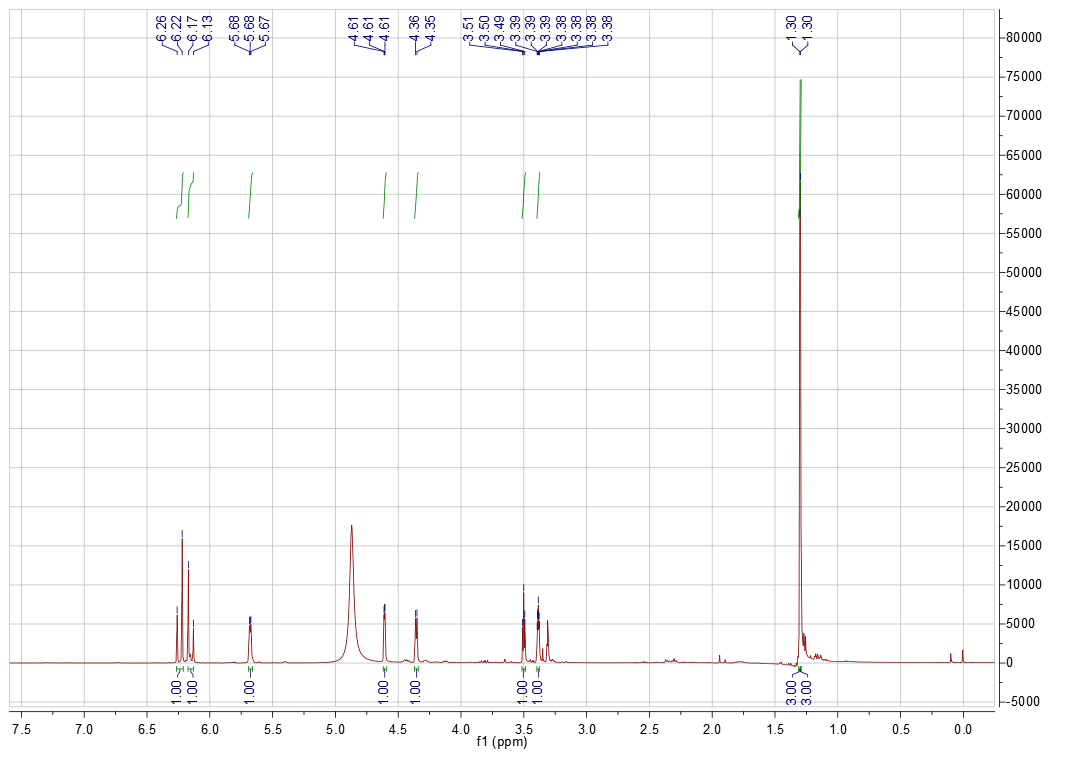


**Figure S35.** ^1^H NMR spectrum (400MHz, CD_3_OD) of **6**


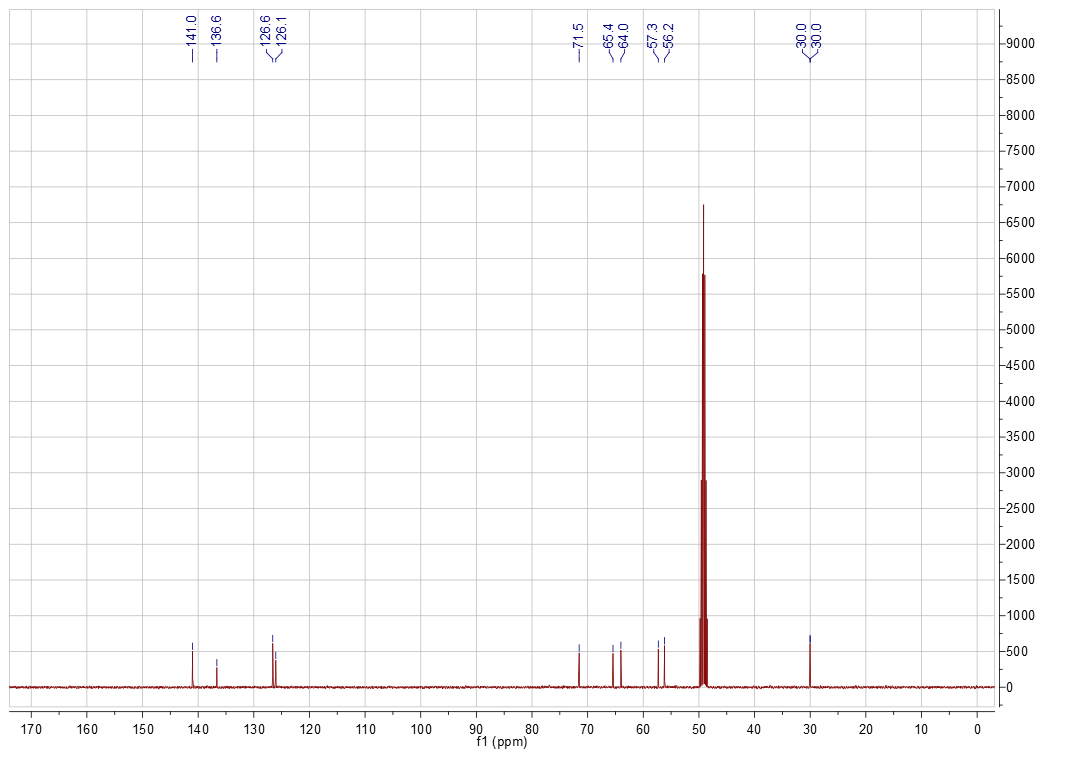


**Figure S36.** ^13^C NMR spectrum (101MHz, CD_3_OD) of **6**


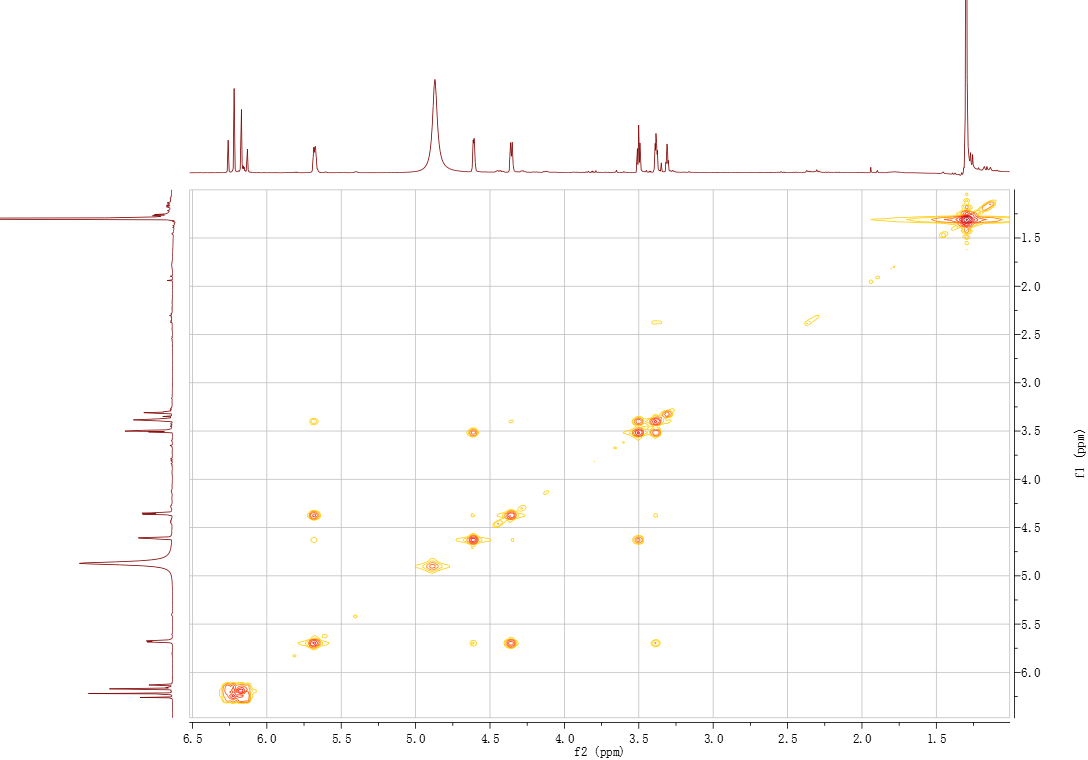


**Figure S37.** ^1^H-^1^H COSY spectrum of **6**


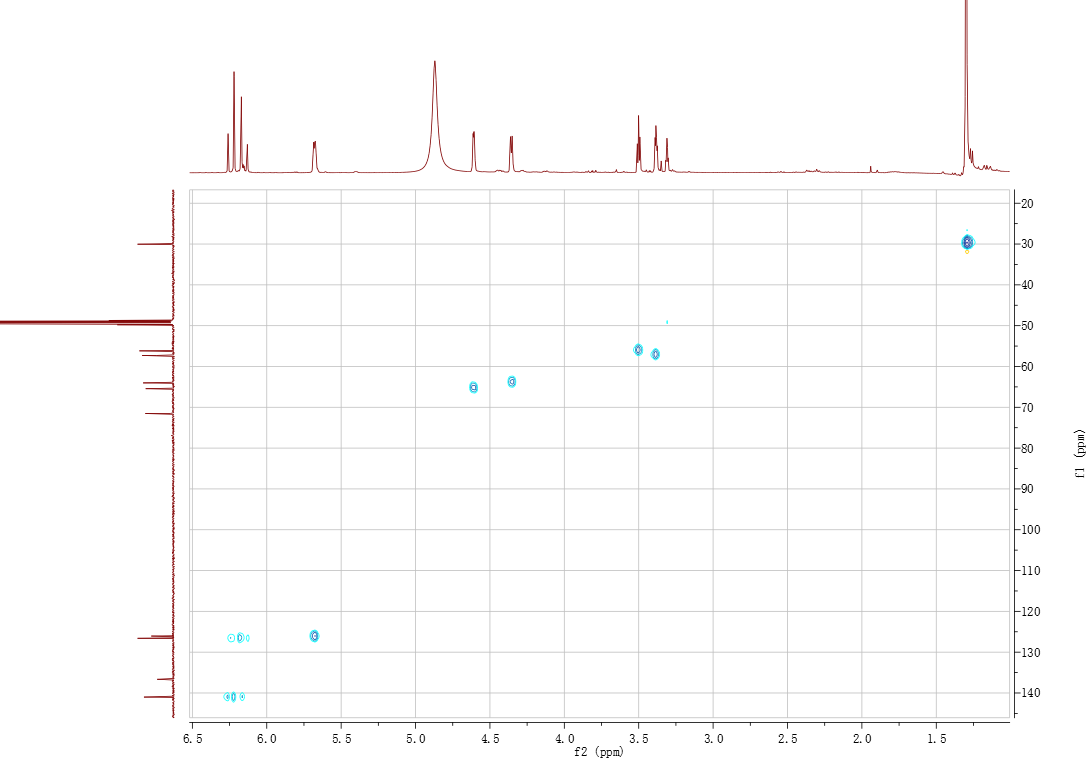


**Figure S38.** HSQC spectrum of **6**


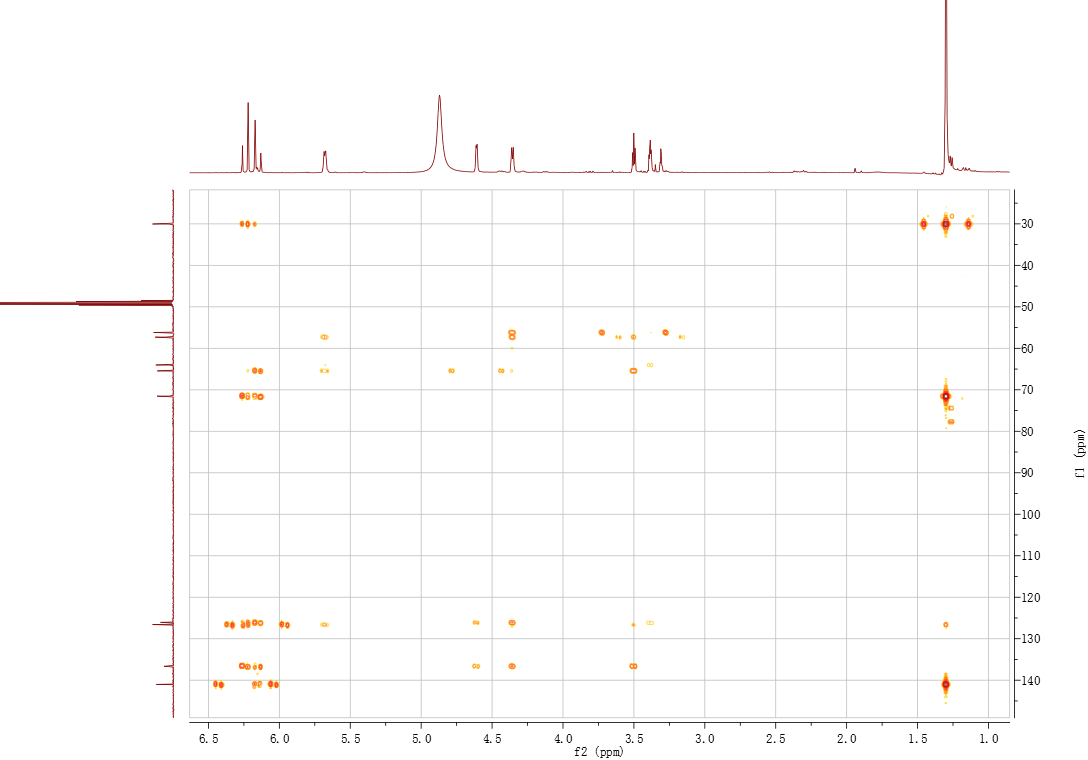


**Figure S39.** HMBC spectrum of **6**


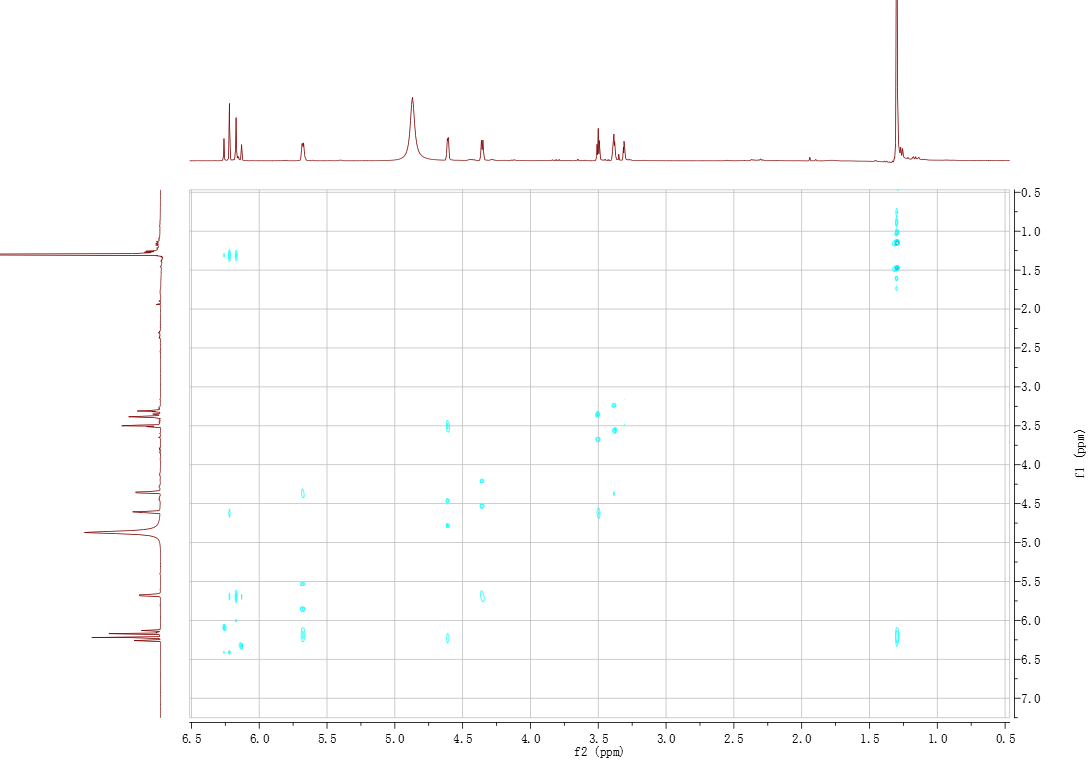


**Figure S40.** NOESY spectrum of **6**


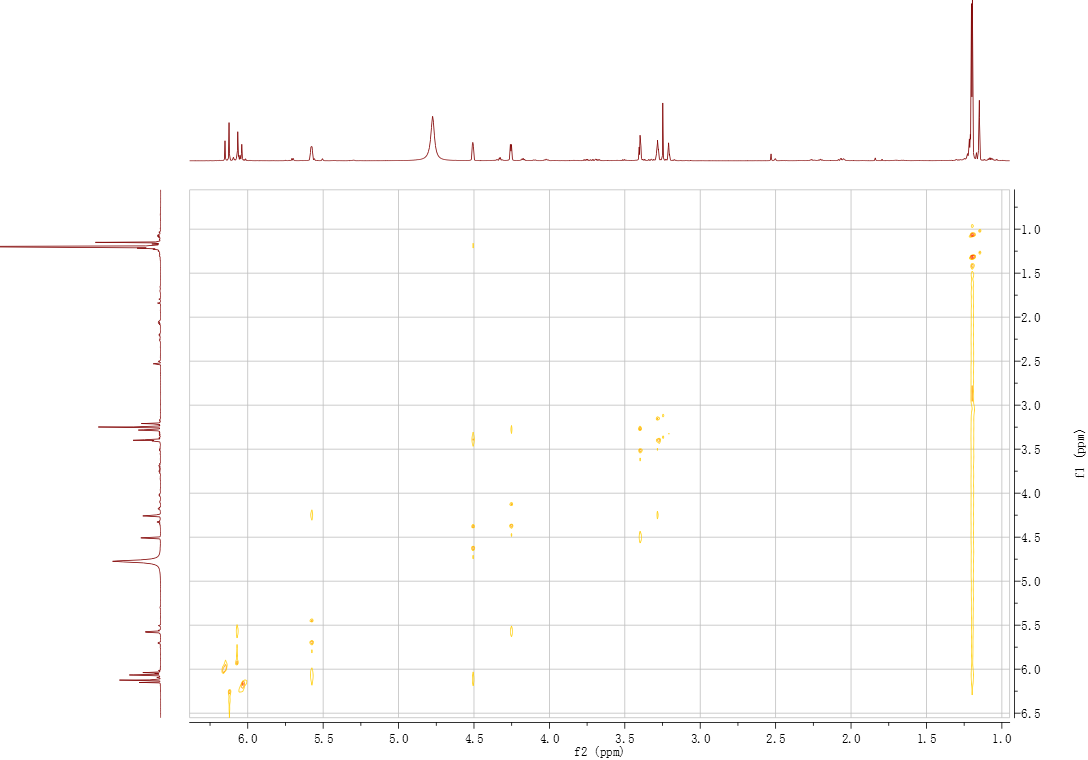


**Figure S41.** ROESY spectrum of **6**

**
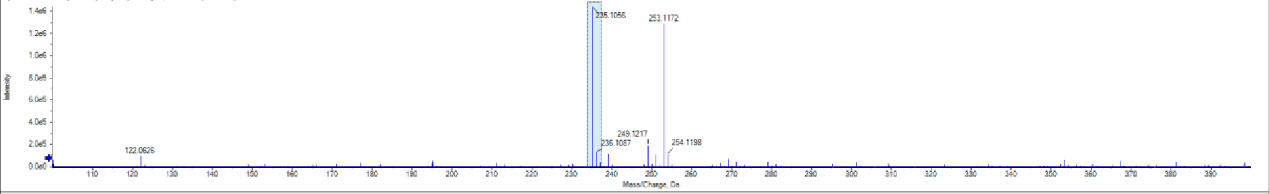
**

**Figure S42.** HR-ESI-MS spectrum of **6**

**
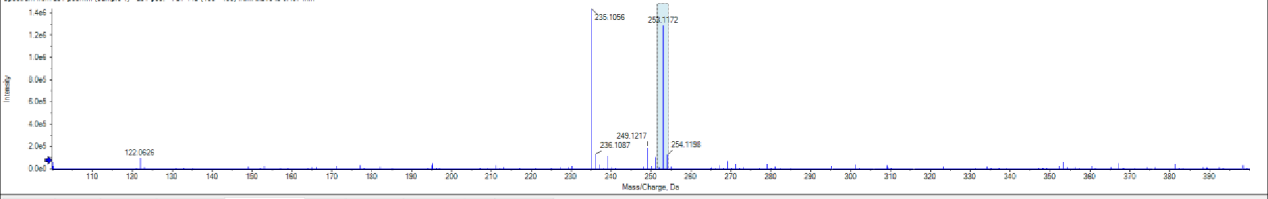
**

**Figure S43.** HR-ESI-MS spectrum of **6**


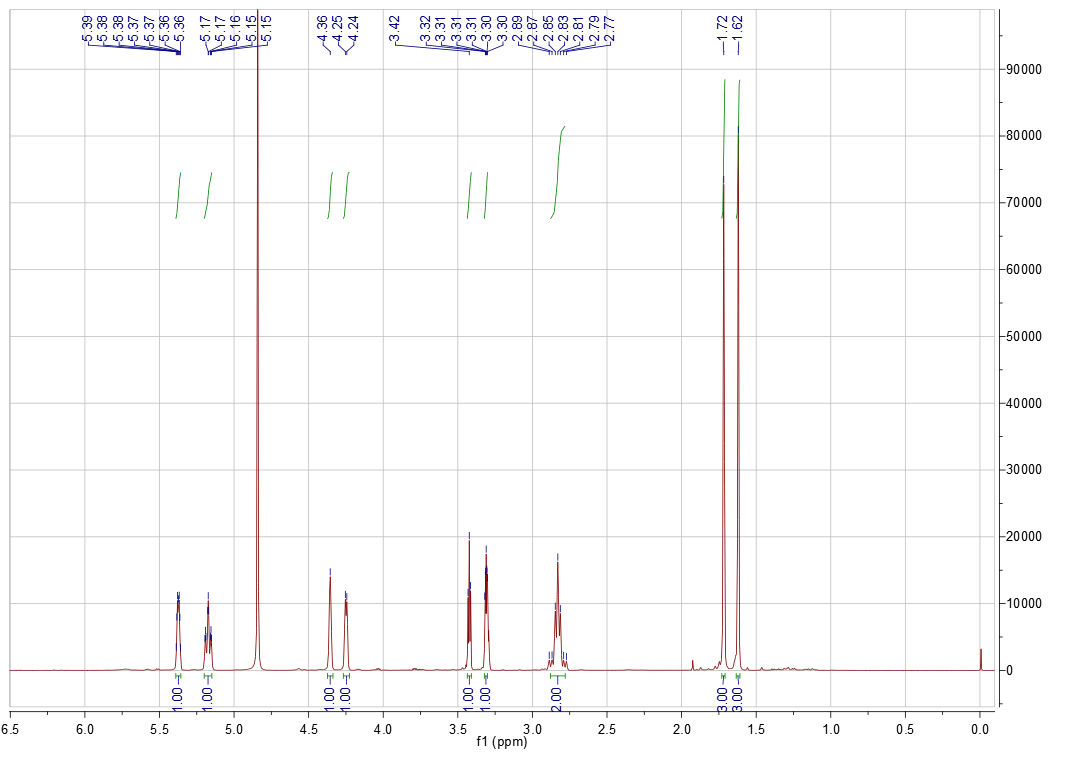


**Figure S44.** ^1^H NMR spectrum (400MHz, CD_3_OD) of **7**


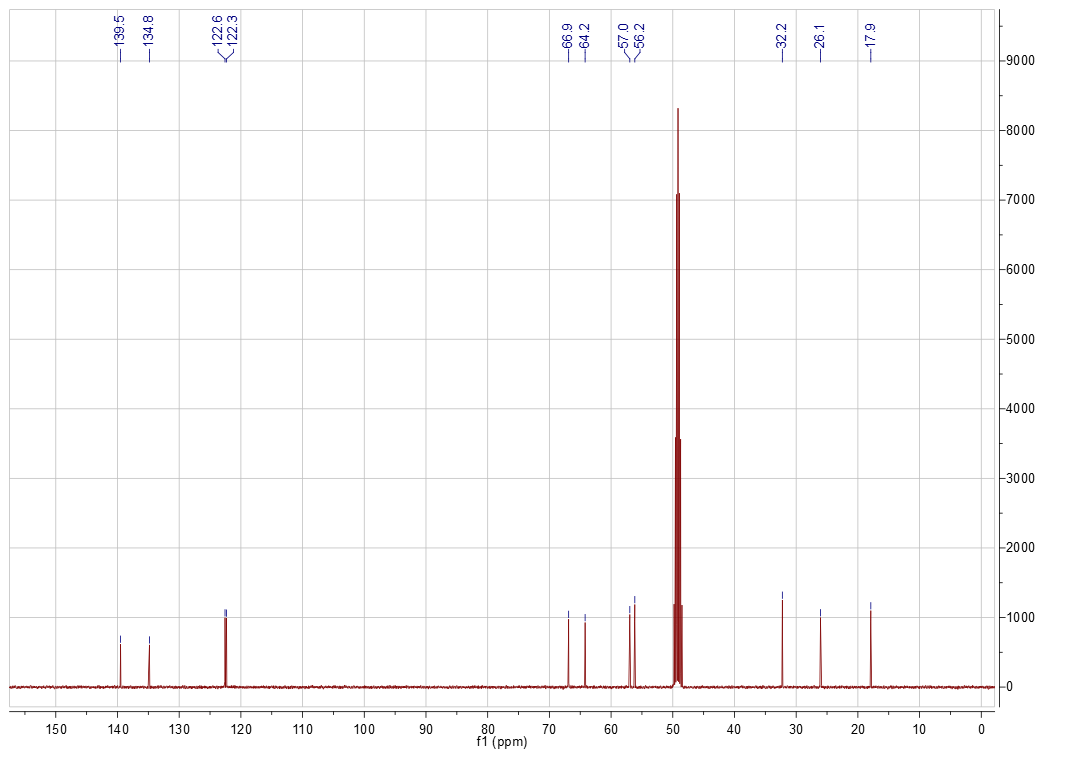


**Figure S45.** ^13^C NMR spectrum (101MHz, CD_3_OD) of **7**


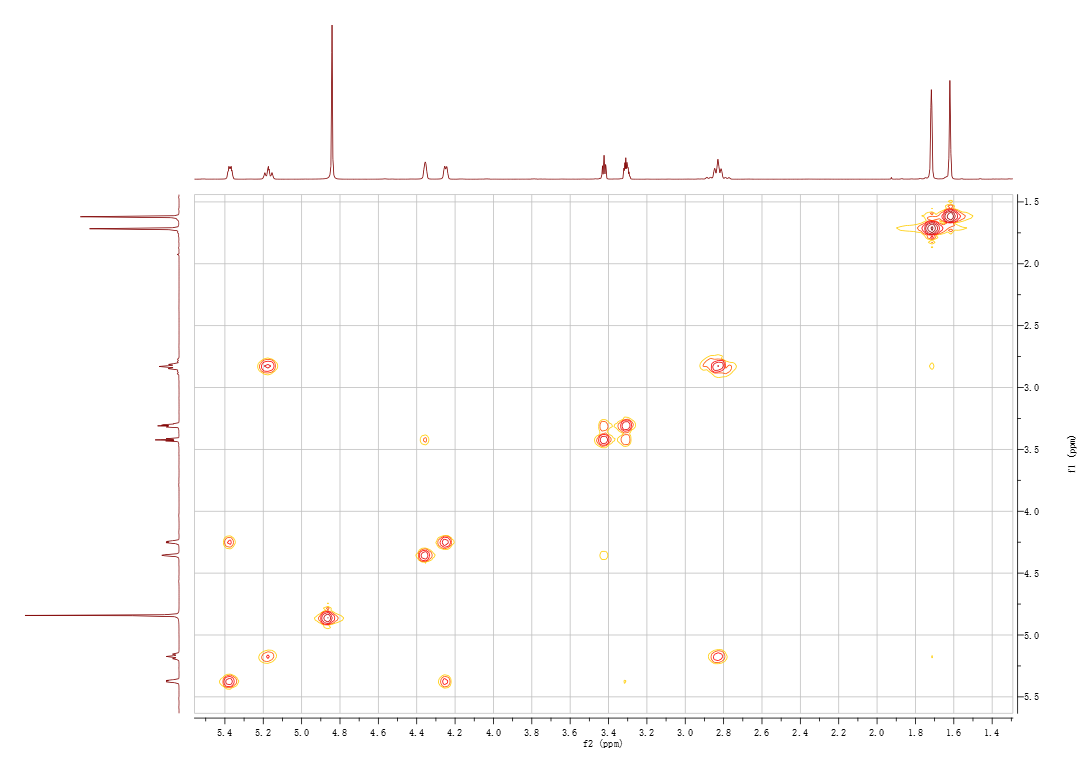


**Figure S46.** ^1^H-^1^H COSY spectrum of **7**


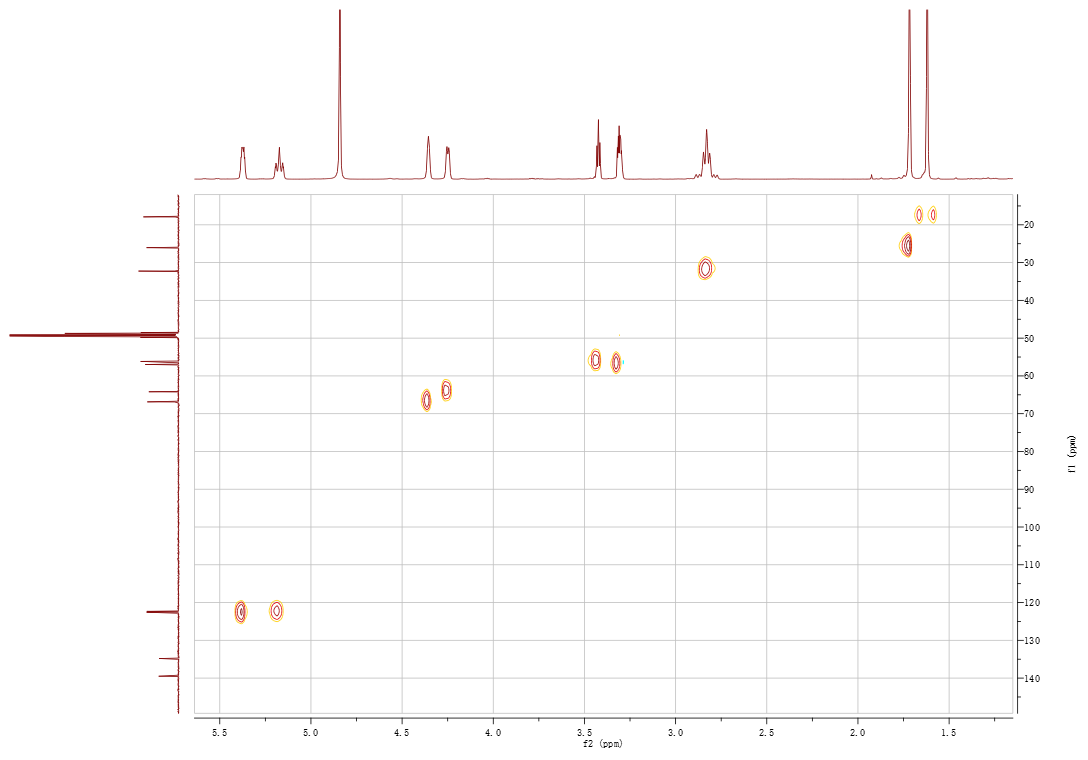


**Figure S47.** HSQC spectrum of **7**


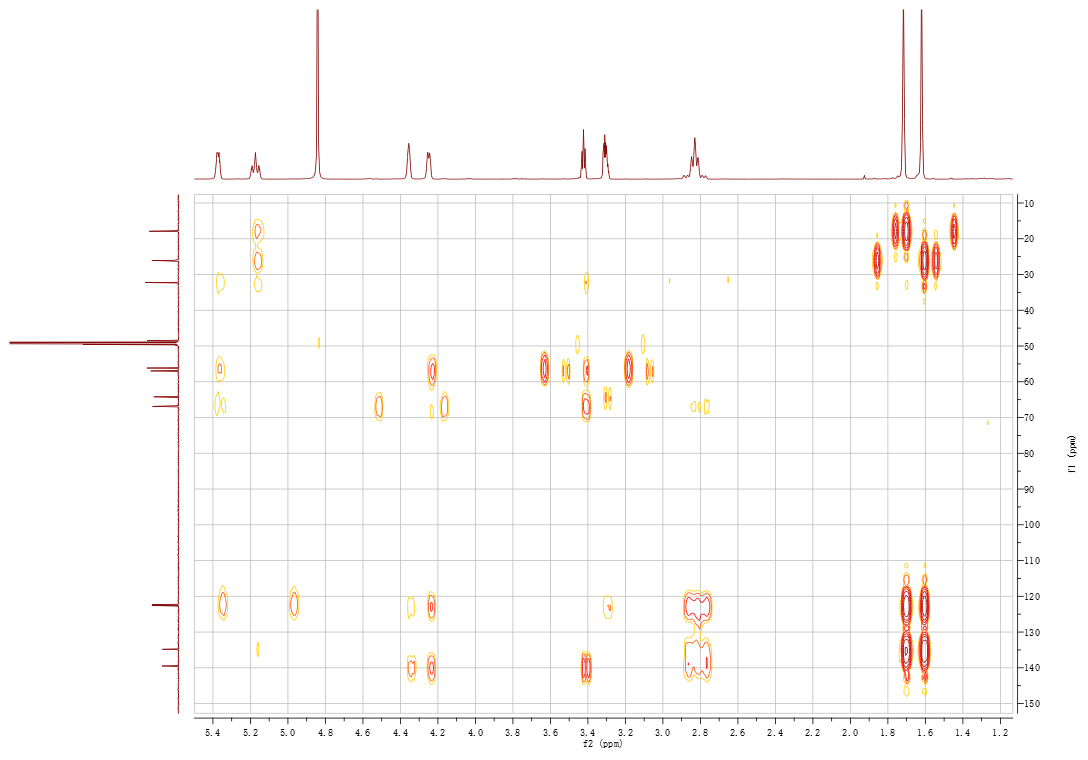


**Figure S48.** HMBC spectrum of **7**


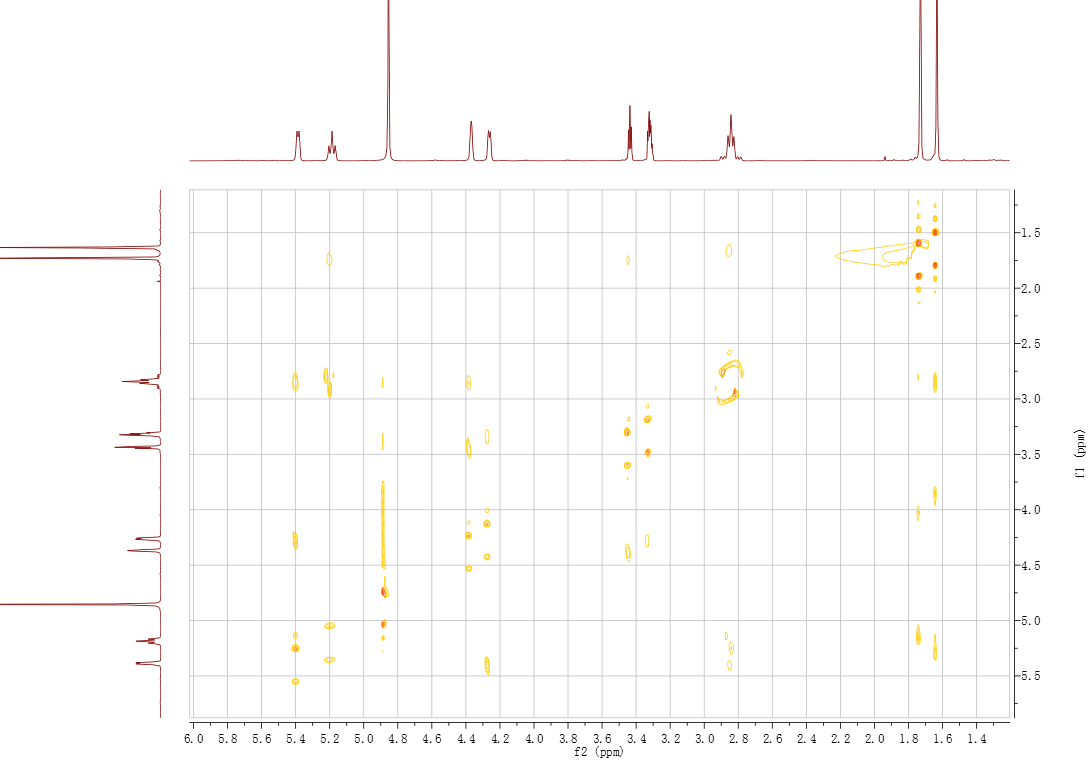


**Figure S49.** NOESY spectrum of **7**


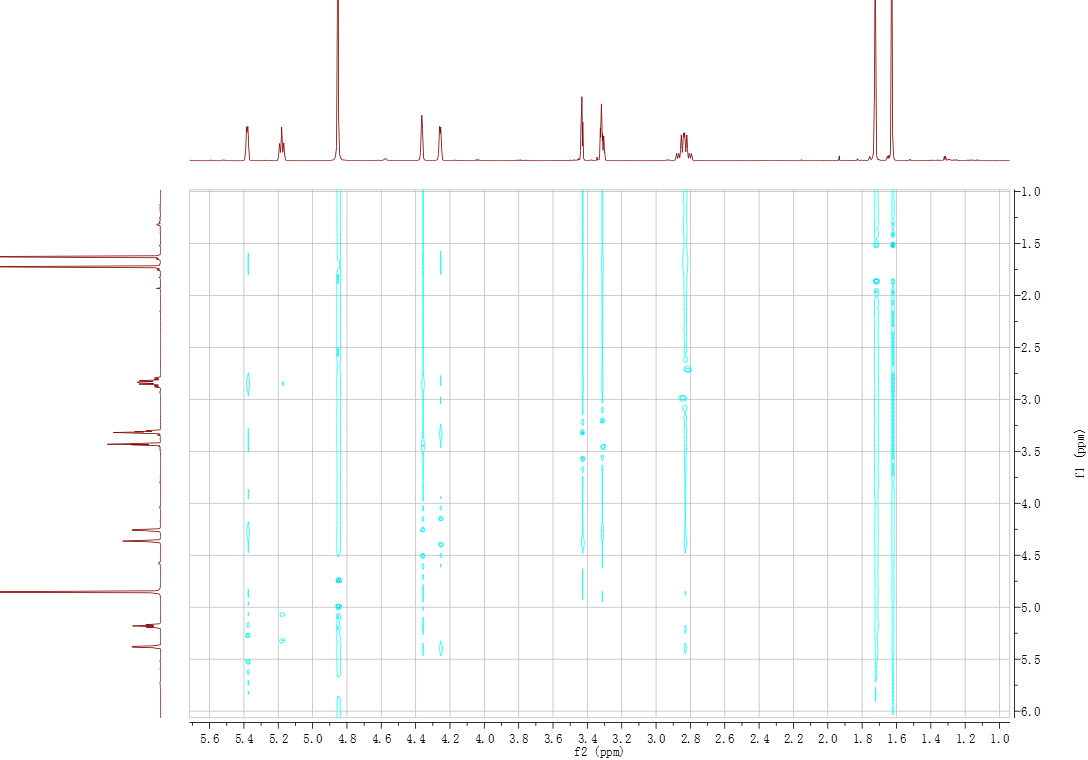


**Figure S50.** ROESY spectrum of **7**

**

**

**Figure S51.** HR-ESI-MS spectrum of **7**


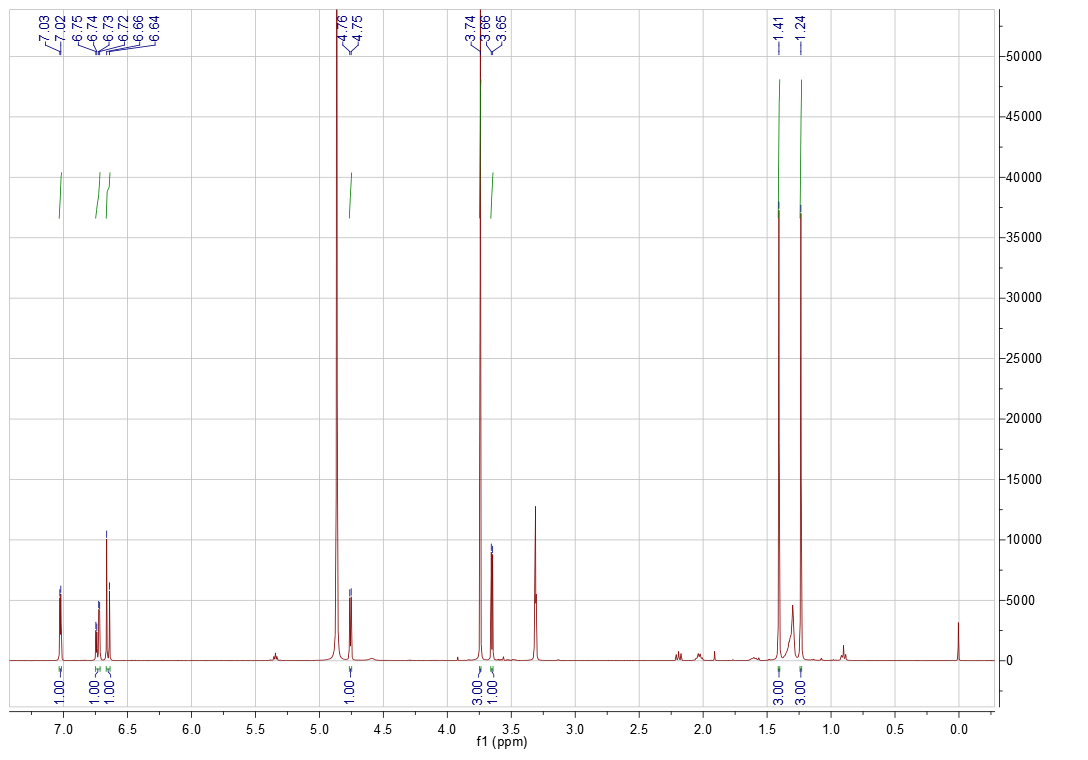


**Figure S52.** ^1^H NMR spectrum (400MHz, CD_3_OD) of **8**


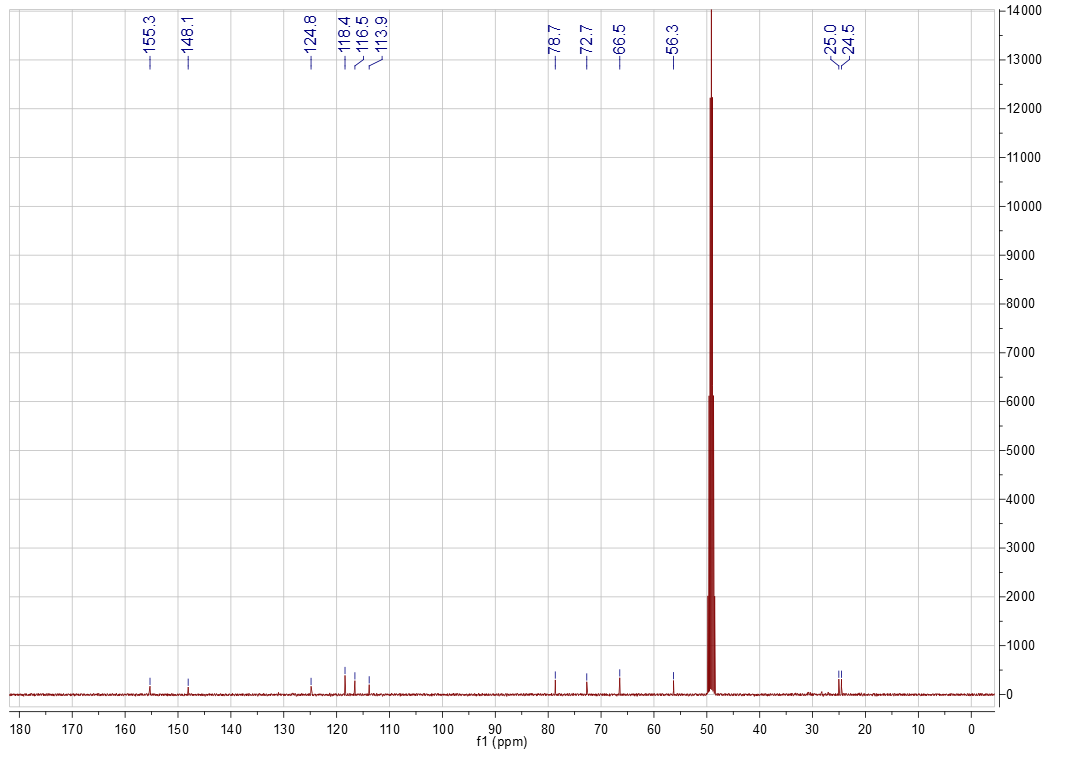


**Figure S53.** ^13^C NMR spectrum (101MHz, CD_3_OD) of **8**


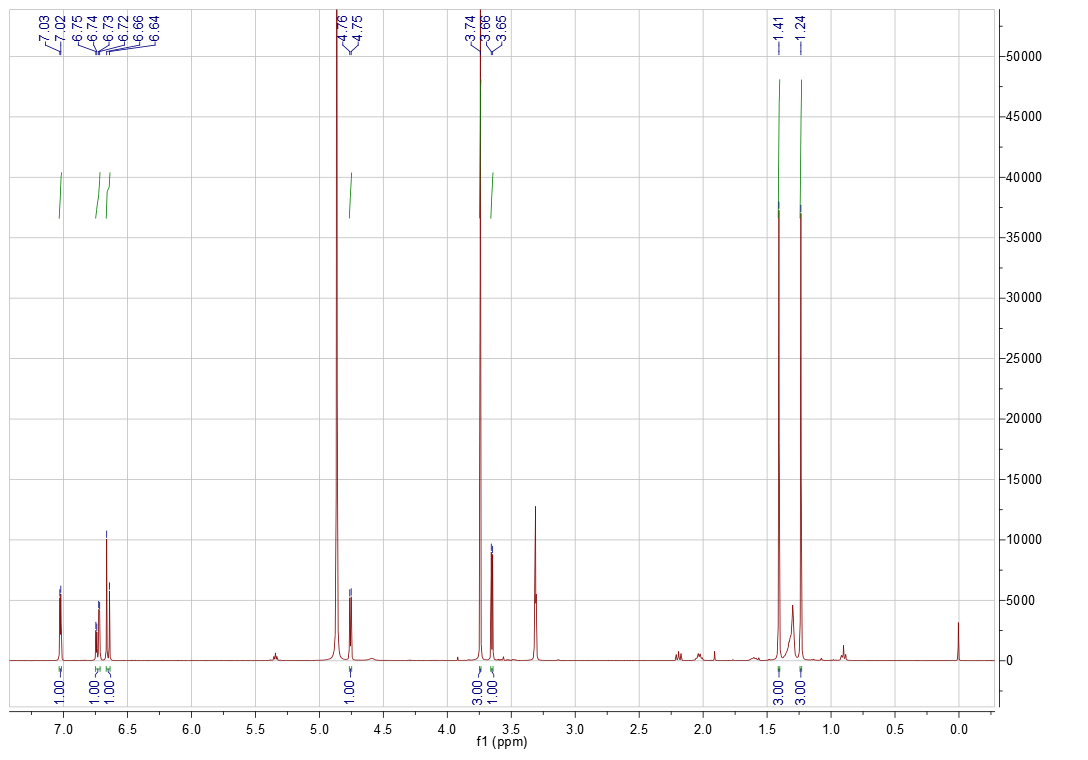


**Figure S54.** ^1^H NMR spectrum (400MHz, CD_3_OD) of **9**


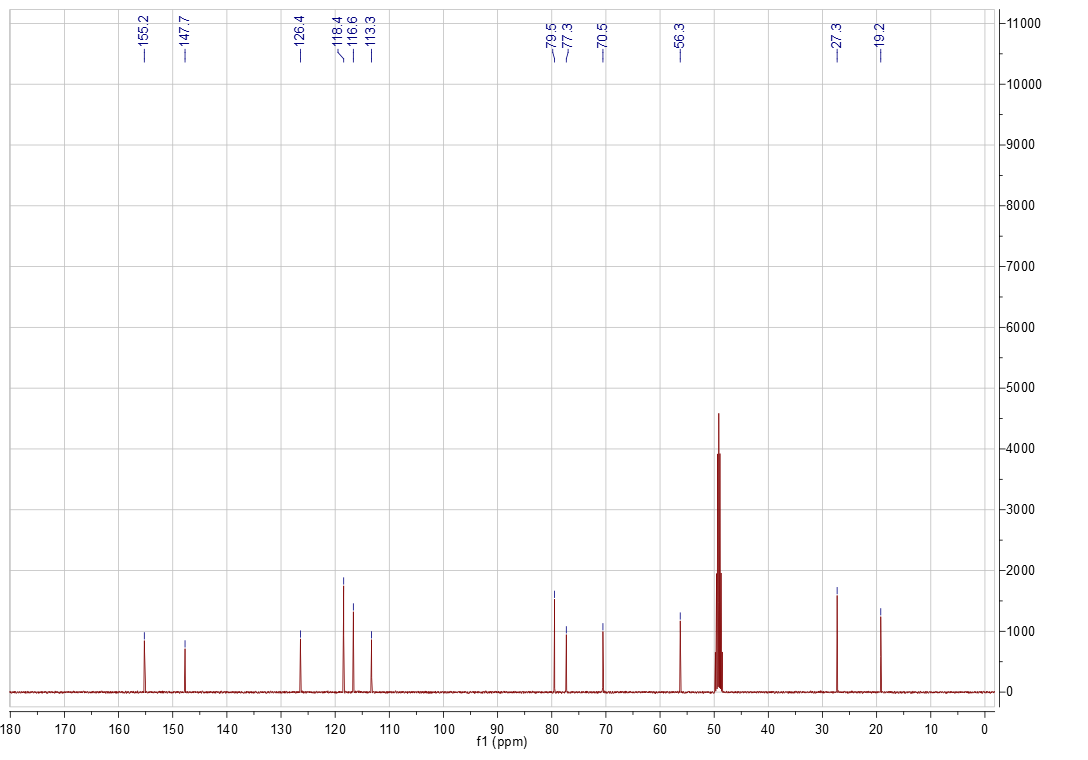


**Figure S55.** ^13^C NMR spectrum (101MHz, CD_3_OD) of **9**


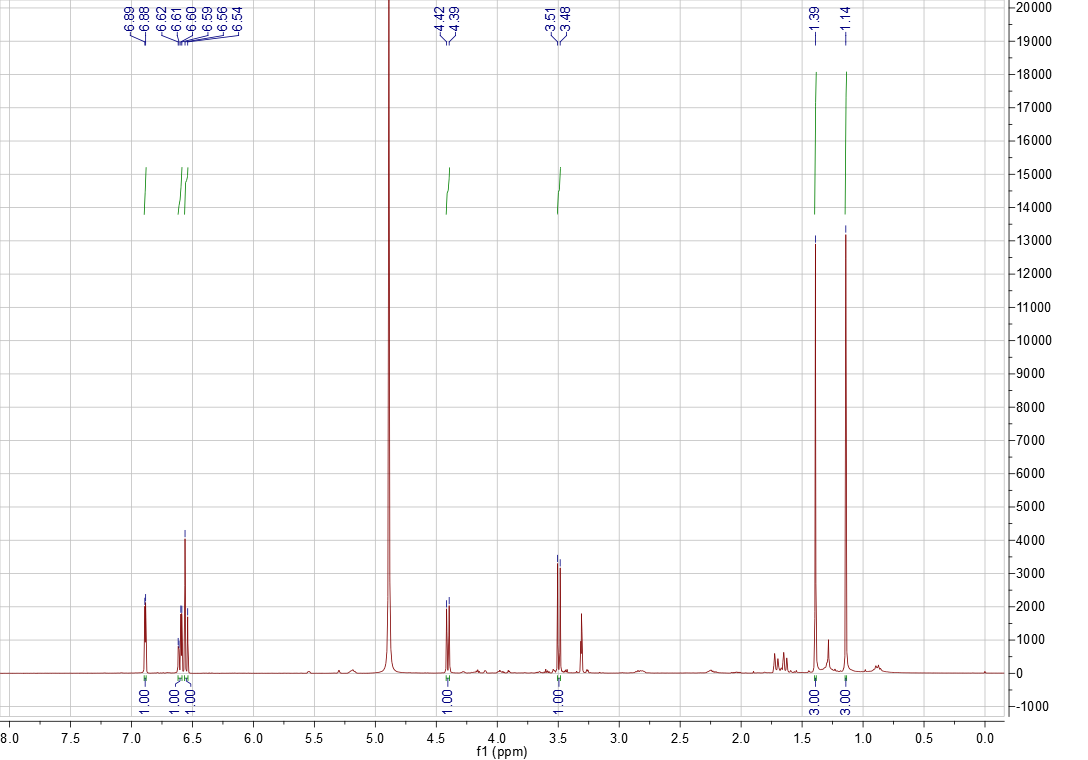


**Figure S56.** ^1^H NMR spectrum (400MHz, CD_3_OD) of **10**


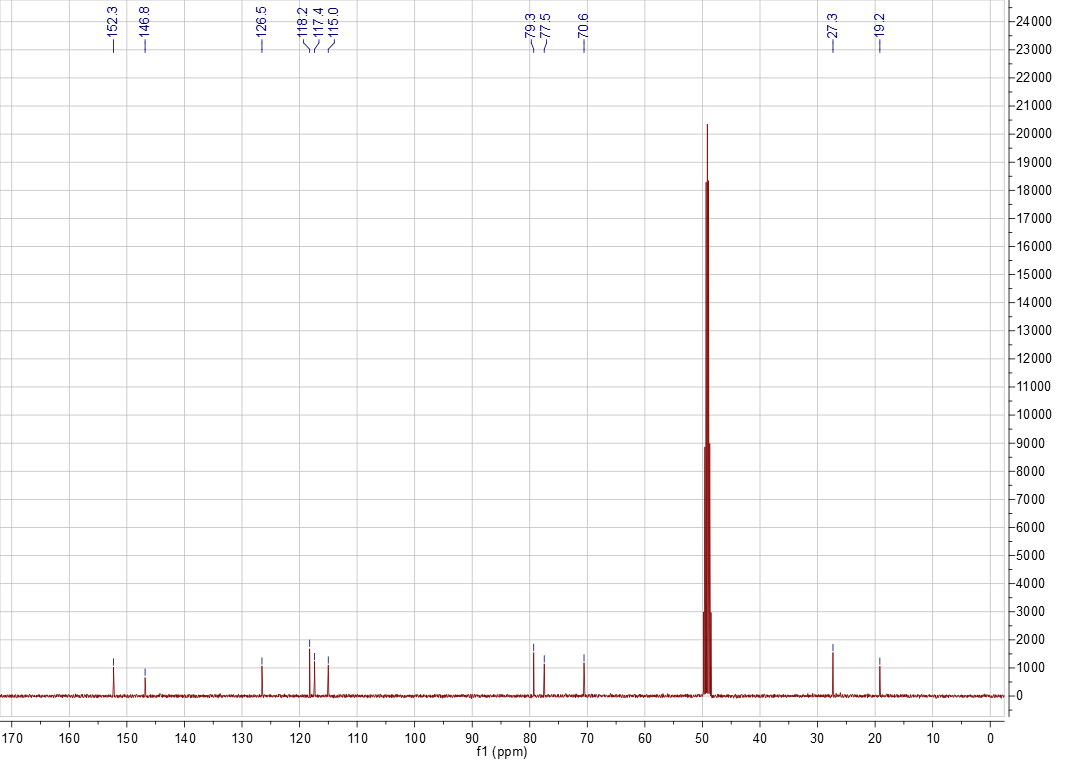


**Figure S57.** ^13^C NMR spectrum (101MHz, CD_3_OD) of **10**


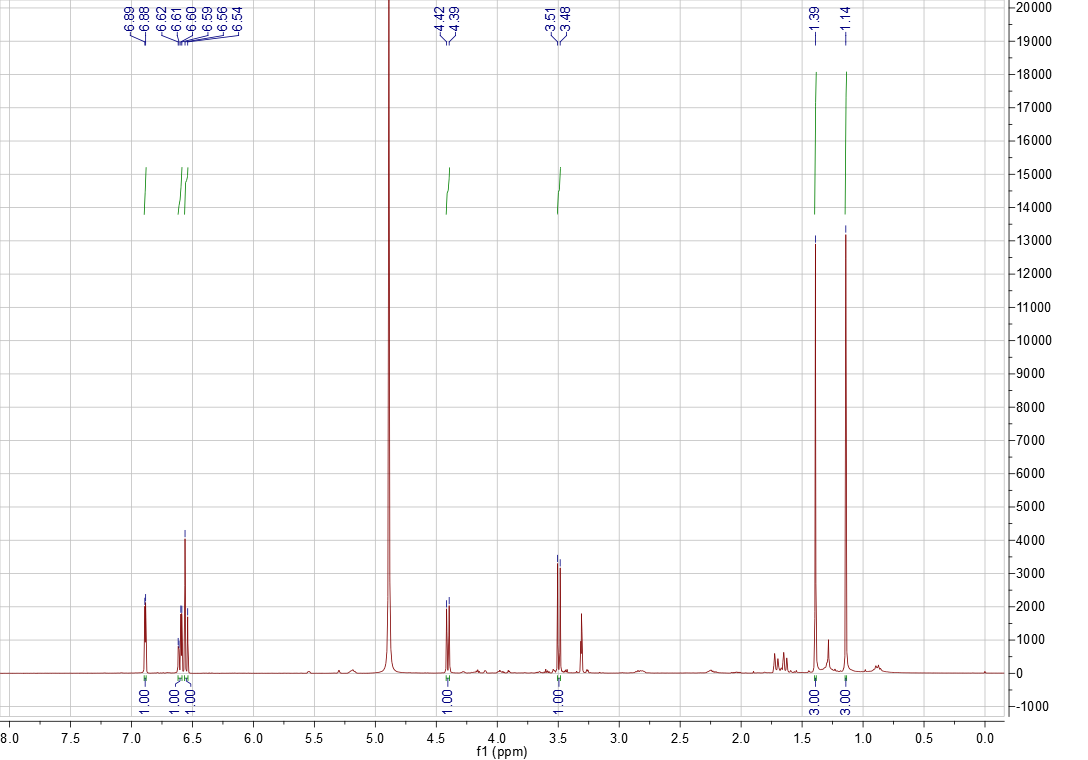


**Figure S58.** ^1^H NMR spectrum (400MHz, CD_3_OD) of **11**


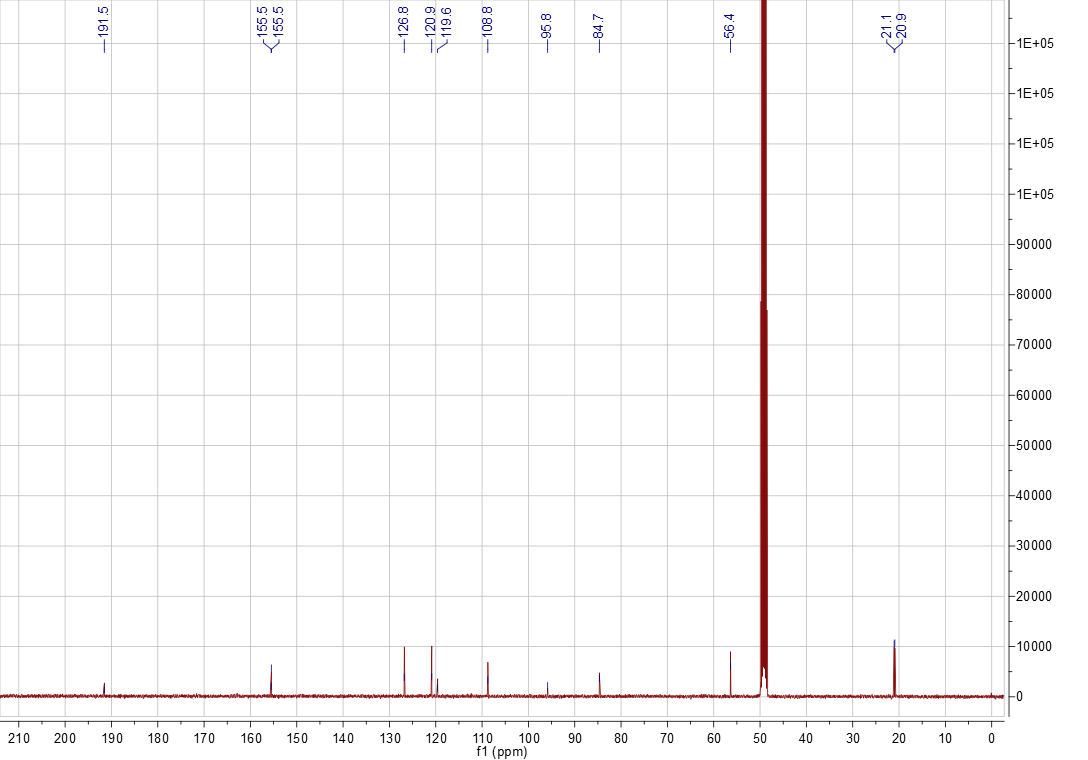


**Figure S59.** ^13^C NMR spectrum (101MHz, CD_3_OD) of **11**


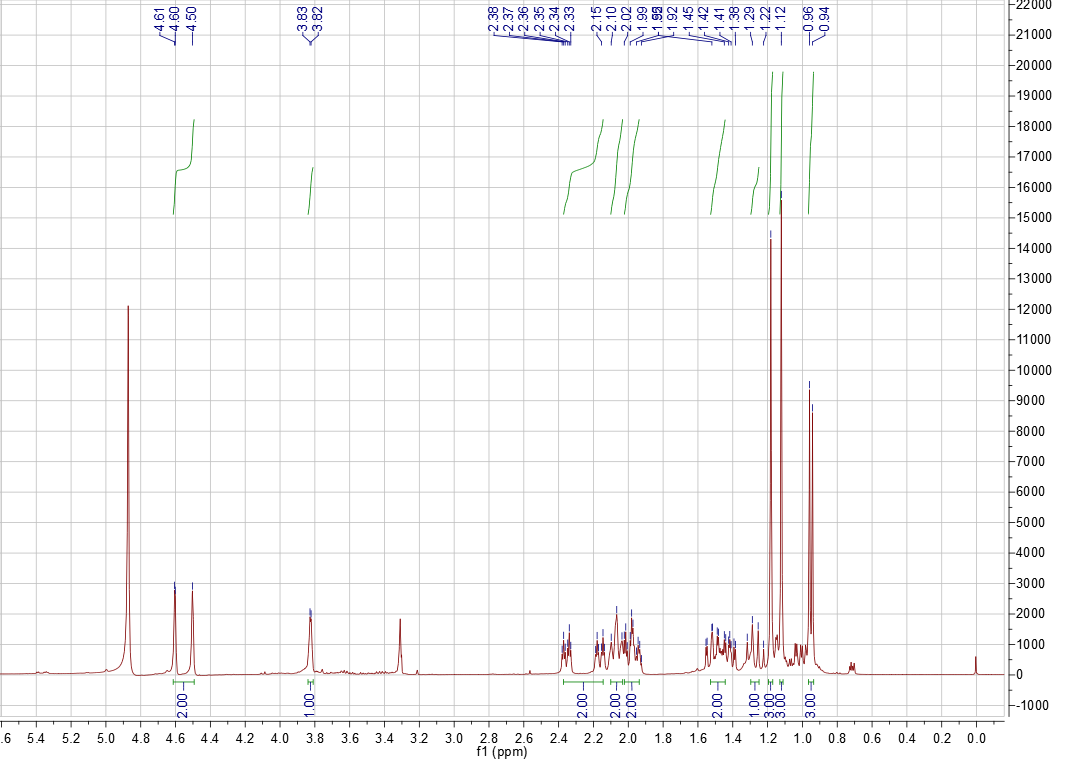


**Figure S60.** ^1^H NMR spectrum (400MHz, CD_3_OD) of **12**


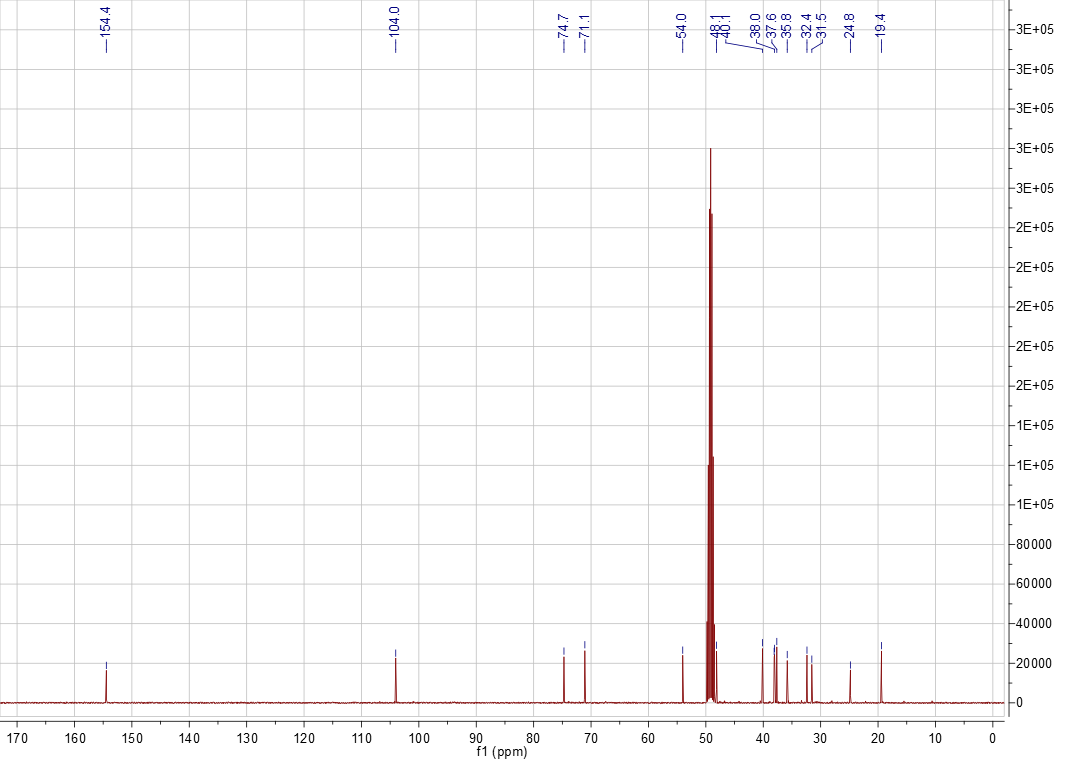


**Figure S61.** ^13^C NMR spectrum (101MHz, CD_3_OD) of **12**
